# In Silico Identification of Novel Immunogenic Secreted Proteins of *Mycoplasma bovis* from Secretome Data and Experimental Verification

**DOI:** 10.3390/pathogens9090770

**Published:** 2020-09-21

**Authors:** Ihsanullah Shirani, Hui Zhang, Gang Zhao, Siyi Lu, Marawan A Marawan, Ali Dawood, Yingyu Chen, Xi Chen, Jianguo Chen, Changmin Hu, Huanchun Chen, Aizhen Guo

**Affiliations:** 1 The State Key Laboratory of Agricultural Microbiology, Wuhan 430070, China; i.shirani786@yahoo.com (I.S.); dkyzhanghui@163.com (H.Z.); happygang@outlook.com (G.Z.); 18202776874@163.com (S.L.); marawan.adel@fvtm.bu.edu.eg (M.A.M.); ali.dawood@vet.usc.edu.eg (A.D.); chenyingyu@mail.hzau.edu.cn (Y.C.); hcm@mail.hzau.edu.cn (C.H.); chenhch@mail.hzau.edu.cn (H.C.); 2 College of Veterinary Medicine, Cooperative Innovation Centre of Substantial Pig Production, Huazhong Agricultural University, Wuhan 430070, China; chenxi@mail.hzau.edu.cn (X.C.); chenjg@mail.hzau.edu.cn (J.C.); 3 Para-Clinic Department, Faculty of Veterinary Medicine, Nangarhar University, Jalalabad 2601, Afghanistan; 4 Infectious diseases, Animal Medicine Department, Faculty of Veterinary Medicine, Benha University, Qualyobia 13511, Egypt; 5 Infectious Diseases, Animal Medicine Department, Faculty of Veterinary Medicine, Sadat City University, Sadat City 32511, Egypt; 6 National Animal Tuberculosis Para-Reference Laboratory (Wuhan) of Ministry of Agriculture and Rural Affairs, Huazhong Agricultural University, Wuhan 430070, China; 7 Hubei International Scientific and Technological Cooperation Base of Veterinary Epidemiology, Huazhong Agricultural University, Wuhan 430070, China

**Keywords:** *Mycoplasma bovis*, secretory proteins, immunoinformatics, antigenicity, epitopes, inflammatory response

## Abstract

*Mycoplasma bovis* is a major pathogen, responsible for bovine respiratory diseases worldwide. The present lack of effective control measures leaves cattle owners at considerable perpetual risk of *M. bovis* outbreaks. In this study, we identified *M. bovis* secreted immunogenic proteins in silico as potential candidates for novel diagnostic agents and vaccines. We used immunoinformatics to analyze 438 *M. bovis* proteins previously identified with a label-free proteomics analysis of virulent *M. bovis* HB0801 (P1) and its attenuated P150 strains. The subcellular localization of these proteins was preliminarily screened and 59 proteins were found to be secreted extracellular proteins. Twenty-seven of these proteins contained a large number of predictive T-cell epitopes presented by major histocompatibility complex (MHC) class I and II molecules. Twenty-two of these 27 proteins had a high number of conformational B-cell epitopes, predicted from the corresponding 3D structural templates, including one unique to P1, two unique to P150, and 19 common to both strains. Five proteins were selected for further validation, and two of these, MbovP274 and MbovP570, were successfully expressed and purified. Both were confirmed to be secretory and highly immunogenic proteins that induced a mouse antibody response, reacted with cattle serum positive for *M. bovis* infection, and significantly increased the production of interleukin 8 (IL-8), IL-12 and interferon *γ* (IFN-*γ*) during the secretion of these three cytokines by both *M. bovis* mutants of these genes. These results should be useful in the development of novel immunological agents against *M. bovis* infection.

## 1. Introduction

*Mycoplasma bovis* (*M. bovis*) belongs to the genus *Mycoplasma* in the class Mollicutes and is the smallest self-replicating organism that lacks a cell wall [1]. It is a major infectious agent of beef and dairy cattle, causing several clinical conditions, including pneumonia, mastitis, arthritis, etc. In China, *M. bovis* was first reported to be associated with pneumonia in cattle in 2008. Since then, *M. bovis* pneumonia has been reported widely in various parts of China, with dramatic economic losses [2,3].

Like other *Mycoplasma* species, *M. bovis* is intrinsically resistant to a wide variety of antimicrobial drugs because it lacks cell wall, a trait that confers tolerance of various effective chemotherapeutic agents commonly used in veterinary medicine. Therefore, antibiotics usually exert a poor chemotherapeutic effect. Consequently, the development of safe and effective vaccines is the best way to control mycoplasma-related illnesses, save animal lives, and minimize the losses attributable to these diseases. The examination of candidate immunogenic agents is essential to the development of effective vaccines or diagnostic agents against *M. bovis* [1,4].

Bacterial secreted proteins can be protective immunogens, toxins, virulence-related factors, etc. [5,6,7,8]. Some extracellular endonucleases from *Serratia marcescens* can degrade the DNA in sputum to clear the airway during respiratory infection. Several studies have suggested that secreted proteins are the best protective antigens and contribute to immunological protection by inducing innate and acquired immune response [7,8,9]. Secretory proteins, such as LptD and LptE of *Salmonella typhi* [5,10] and ORF01609, ORF01830, ORF01839, ORF02943, ORF03355, and ORF03641 of *Aeromonas hydrophila*, play significant roles in this protection and may be the best immunogenic agents in bacterial infections [5,10]. The importance and vital role of the bacterial secretome means that these proteins are potential new immunogenic agents, biomarkers, and drug targets. For example, four secreted *Aspergillus fumigatus* proteins, Afu5g14380, Afu8g01670, Afu1g09900, and Afu6g09740, that are upregulated during infection might be novel drug targets in the treatment of aspergillosis [11]. The secretory proteins of *Mycobacterium tuberculosis*, such as ESAT-6, MTSA-10, CFP-1, and Rv1860, have well-known immunomodulatory activity and are utilized for therapeutic, diagnostic, and vaccination purposes [9,12]. The secretory proteins of *Francisella tularensis* also play significant roles in the infection process, immune evasion, virulence, intracellular survival, and vaccine development [7]. However, to date, few secretory proteins of *M. bovis* have been identified. However, preliminary studies have demonstrated that they play significant roles in inducing immunity and as diagnostic biomarkers [13,14].

Various proteomics approaches have been used to identify the mycoplasmal secretome [13,15]. Because the growth of mycoplasma species is complicated by their nutritional requirements, their extracted secreted proteins usually include a high proportion of either proteins produced during culture in nutrient medium enriched with serum and yeast extract, or self-degraded -proteins produced during growth in poor nutrient medium or PBS. Therefore, it is necessary to efficiently differentiate real secreted proteins from those derived from the culture process.

Because the cytokines interleukin 12 (IL-12), interferon *γ* (IFN-*γ*), and IL-17 regulate the immune response during the course of infection, a study of specific cytokines may be an efficient way to define the immunological secreted proteins of *M. bovis* strains [16]. Immunoinformatic tools allow for the systemic identification of antigens, provide a basis for their experimental validation, and reduce the high cost of cloning and expressing genes encoding putative secreted proteins and the subsequent validation of individual proteins [17].

We previously isolated an attenuated strain of *M. bovis* HB0801-P150, by continuously growing the wild-type *M. bovis* HB0801 P1strain in vitro for 150 passages and determined the secretome data for both strains with a label-free proteomic approach. The aim of this study was to identify the secreted immunological proteins of both strains from their secretome data using an in silico immunoinformatic analysis. In this way, we identified 22 immunogenic secreted proteins and confirmed that MbovP274 (in P1) and MbovP570 (in both P1 and P150) are immunogenic secreted proteins. They also induced the expression of IL-8, IL-12 and IFN-*γ*. These findings clarify the pathogenesis *M. bovis* and the immune response it induces and provide potential candidate proteins for the development of diagnostic reagents and vaccines to control *M. bovis* associated diseases.

## 2. Results

**Figure 1:**
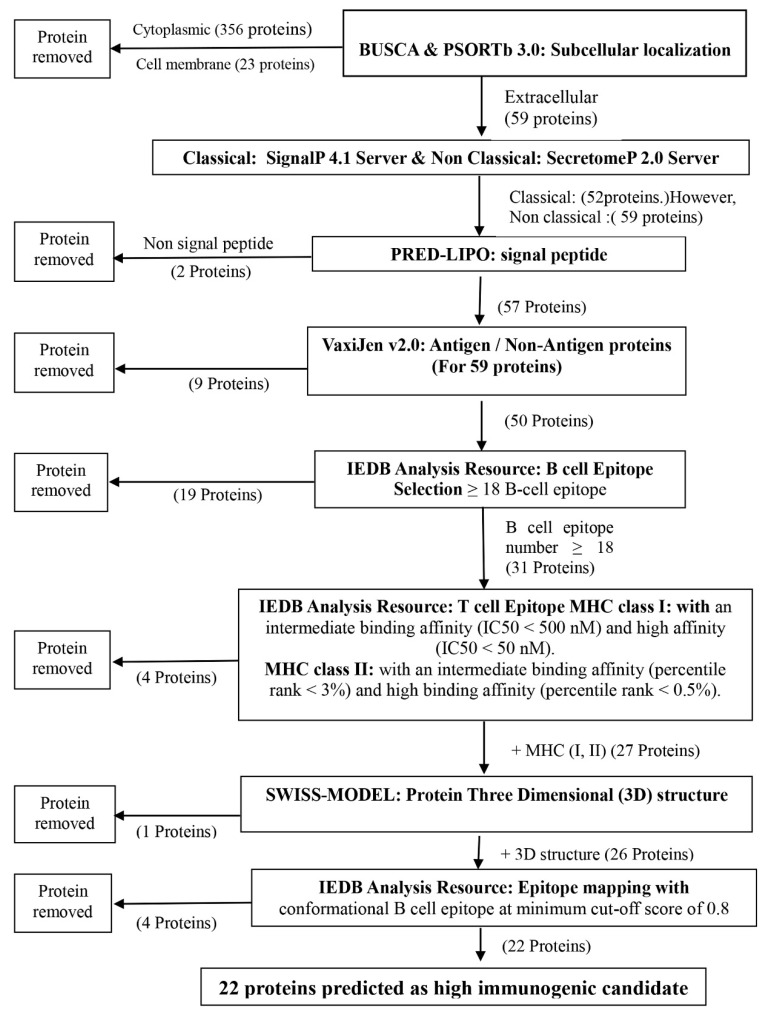
Flowchart summarizing the steps to complete the identification of high immunogenic secreted proteins of *M. bovis*.

A flowchart of the overall procedures in the in silico analysis and the identification of immunogenic secretory proteins of *M. bovis* is presented in Figure 1. Of the 438 proteins previously identified with label-free proteomic analysis, 59 were found to be extracellular secreted proteins. Among these 59 proteins, 27 contained a high number of predicted T-cell epitopes presented by major histocompatibility complex (MHC) class I and II molecules and B-cell epitopes. Furthermore, 22 of these 27 proteins contained a high number of conformational B-cell epitopes predicted from the corresponding three-dimensional (3D) structural templates.

### 2.1. Prediction of Protein Subcellular Localization

A label-free approach previously identified a total of 438 proteins from *M. bovis* HB0801 (P1) and P150 strains, with 426 proteins from P1 and 375 from P150. Among these, the BUSCA server predicted 357 cytoplasmic proteins, 58 extracellular proteins, and 23 plasma membrane proteins (Table S1).

However, based on predictive PSORTb 3.0 analysis, 263 proteins were cytoplasmic, six extracellular, and 38 were plasma membrane proteins, and the other 131 were classified as unknown (Table S2).

Among the six predicted extracellular proteins, (MbovP211, MbovP341, MbovP473, MbovP580, MbovP693, and MbovP732), only MbovP732 was not included in the BUSCA results. Based on the precision of the PSORTb server, we included it among the extracellular proteins. Ultimately, 59 proteins were predicted as extracellular. Among these, three were detected in *M. bovis* HB0801 (P1), 11 in P150, and 45 in both strains (Table 1).

### 2.2. Prediction of Secreted Proteins

The Signal P4.1 server identified 52 of these 59 predicted extracellular proteins as classical secretory proteins (two in P1, 10 in P150, and 40 in both) with scores above 0.45 (cut-off score). In contrast, seven proteins had a score <0.45 and no signal sequence (Table 1). Fifty-nine proteins were predicted to be non-classical secretory proteins (three in P1, 11 in P150, and 45 in both) by Secretome P2.0, with scores >0.5 (cut-off score). Moreover, PRED-LIPO predicted that 57 proteins (two in P1, nine in P150, and 46 in both) had a signal peptide (Table 1), whereas two proteins had no signal peptide. The overall results are shown in Table S3.

### 2.3. Analysis of Linear B-Cell and MHC Class I and Class II T-Cell Epitopes

The VaxiJen v2.0 server predicted 50 of these 59 proteins to be antigenic, with overall antigen prediction score >0.4. In contrast, nine proteins had scores below the default setting score and thus appeared to be non-antigenic. Of those 50 predicted antigenic proteins, one belonged to P1, seven to P150, and 42 to both strains (Table 1).

Based on the IEDB-AR output results, 31 antigenic proteins (one in P1, four in P150 and 26 in both) had linear epitopes (Table S4). Therefore, these 31 antigenic proteins were selected as the best immunogenic proteins (Table 2).

The affinity of the T-cell epitopes in each of the 31 antigenic proteins for a 9-mer MHC class I molecules was predicted (Table S5). Twenty-nine proteins were consistently predicted to have high or intermediate-affinity epitopes, whereas two proteins (MbovP538 and MbovP798) were identified as having no epitopes of either category. The affinity of the T-cell epitopes for a 15-mer MHC class II molecule was predicted for these proteins. All the proteins had intermediate binding affinity, whereas eight proteins (MbovP016, MbovP038, MbovP274, MbovP306, MbovP517, MbovP570, MbovP579, and MbovP675) had epitopes that bound with high affinity (Table S6).

### 2.4. Overlapping Residues of MHC Class I and II T-cell Epitopes

The MHC class I 9-mer T-cell epitopes were found to overlap with the MHC class II 15-mer T-cell epitopes in most proteins. Twenty-seven *M. bovis* proteins (one in P1, two in P150 and 24 in both) were shown to contain at least one T-cell epitope with binding affinity for both MHC class I and II molecules, whereas four *M. bovis* proteins (MbovP290, MbovP350, MbovP364, and MbovP374) did not contain at least one T-cell epitope with binding affinity for both MHC class I and II molecules (Table 2, Table S7).

### 2.5. 3D Structural Modeling and Conformational B-Cell Epitopes

SWISS-MODEL Workspace identified the 3D structural templates for 26 of the 27 antigenic secreted proteins for modeling. MbovP536 was exclude because SWISS-MODEL found no suitable template. A Protein Data Bank (PDB) ID for each 3D structural template was generated. After sequence alignment with the *M. bovis* proteins, all the modeled templates showed completely different lengths, identities, and PDB ID percentages. Finally, 26 *M. bovis* proteins with high-percentage PDB ID (20–40%) were selected for further analysis (Table S8).

Twenty-two of these proteins (one from P1, two from P150 and 19 from both) were further shown to contain a high number of conformational B-cell epitopes, which were defined according a more stringent cut-off value of 0.8. The 3D images of the predicted epitopes are represented as ball-and-stick models (viewed with Jmol) [17], which showed their spatial locations relative to the protein molecules. The details of their 3D structural templates and B-cell conformational epitopes are shown in Table S8.

### 2.6. Verification of Secretion and Immunogenicity of Recombinant Proteins

The sequences encoding five proteins (MbovP049, MbovP274, MbovP296, MbovP570, and MbovP585) were successfully cloned into the pET–30a (+) vector. However, only two proteins (rMbovP274 and rMbovP570) were successfully expressed in *Escherichia coli* BL21 (DE3).

**Figure 2:**
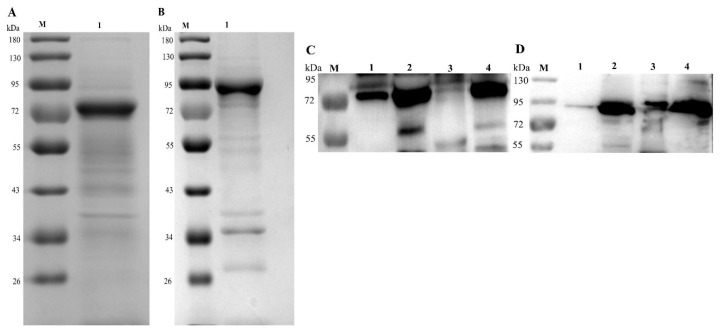
Confirmation of purified recombinant proteins and their secretion. SDS-PAGE (10%) was used to check the recombinant proteins (**A** and **B**). (**A**). Lane 1: rMbovP274, ~71 kDa. (**B**). Lane 1: rMbovP570 ~91 kDa. A Western blotting assay was used to verify the secretion of rMbovP274 (**C**) and rMbovP570 (**D**). Lanes 1 and 2 contain the extracted secretome (lane 1) and whole-cell proteins (lane 2) from the P1 strain, whereas lanes 3 and 4 contain the extracted secretome (lane 3) and whole-cell proteins (lane 4) from the P150 strain. Lane M: reference proteins, with molecular masses labeled on the left.

After their solubility was tested, the His-tagged recombinant Mbov274 (rMbov274) protein was found in the pellet, whereas the His-tagged rMbov570 protein was found in the supernatant. Both proteins are putative lipoproteins. They were purified with nickel chromatography and confirmed with 10% SDS-PAGE. The molecular sizes of rMbovP274 and rMbovP570 were approximately 66 kDa and 86 kDa, respectively, and therefore those of the His-tagged rMbovP274 and rMbovP570 were about 71 kDa and 91 kDa, respectively, and occurred as expected on an SDS-PAGE gel (Figure 2A,B).

The indirect enzyme-linked immunosorbint assay (iELISA) titers of the antisera against rMbovP274 and rMbovP570 were 1:8000, demonstrating the good immunogenicity of the proteins. Western blotting assays with these antisera demonstrated that rMbovP274 was only secreted by P1, whereas rMbovP570 was secreted by both P1 and P150, consistent with our prediction (Figure 2C,D).

**Figure 3:**
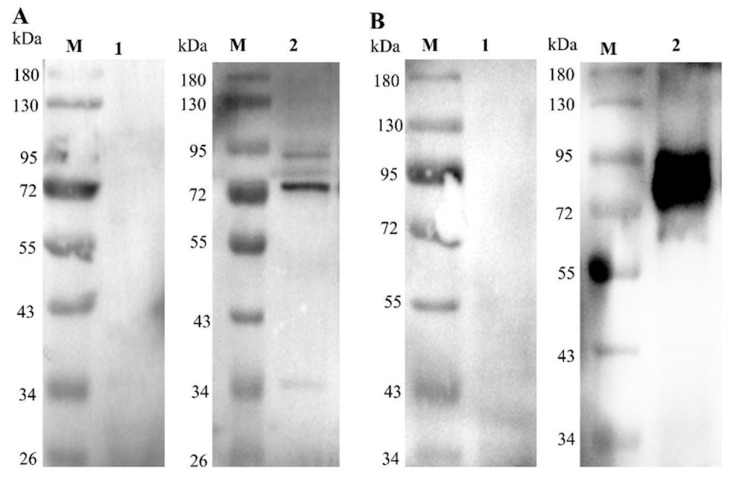
Verification of immunogenicity with cattle serum in a Western blotting assay. (**A**). rMbovP274, ~71 kDa secreted protein; (**B**). rMbovP570 ~91 kDa secreted protein. lane M: protein marker; Lane 1: *M. bovis*-negative cattle serum; lane 2: *M. bovis*-positive cattle serum.

The reactions of rMbovP274 and rMbovP570 with cattle sera either positive or negative for *M. bovis* infection were checked with a Western blotting assay. The results show that both rMbovP274 and rMbovP570 reacted very well with *M. bovis*-positive cattle serum, but not with *M. bovis*-negative serum, and that rMbovP570 reacted more strongly than rMbovP274 (Figure 3).

### 2.7. Inflammatory Cytokines Induced by Secreted rMbovP274 and rMbovP570

**Figure 4:**
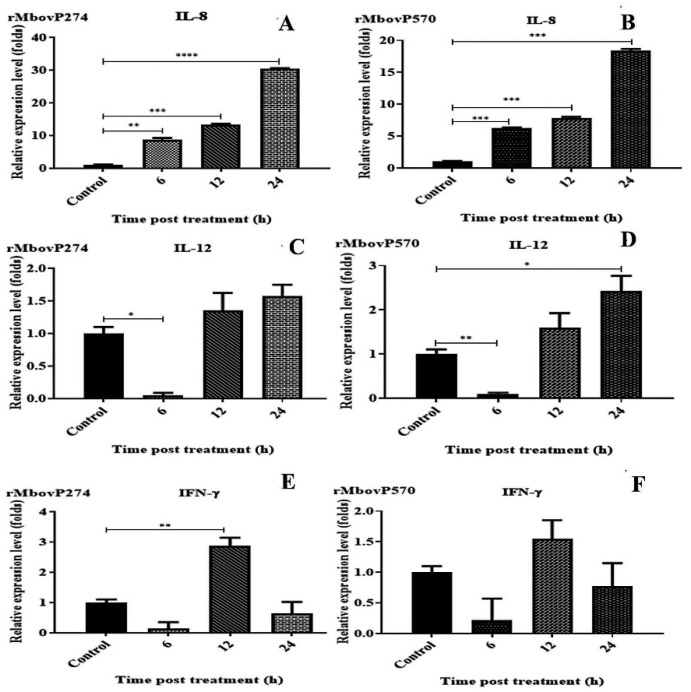
Expressions of cytokines in bovine macrophage cells (BoMac) stimulated with purified *M. bovis* proteins. BoMac cells were incubated with purified rMbovP274 and rMbovP570 for 6, 12, and 24 h, and expression of cytokines was determined with real-time PCR. (**A**,**B**): Expression of interleukin 8 (IL-8) was detected at three time points after treatment with rMbovP274 (**A**) and rMbovP570 (**B**). Expression of IL-8 was significantly higher at all-time points after treatment than in the control cells. (**C**,**D**): Expression of IL-12 was detected at three time points after treatment with rMbovP274 (**C**) and rMbovP570 (**D**). (**E**,**F**): Expression of IFN-*γ* was detected at three time points after treatment with rMbovP274 (**E**) and rMbovP570 (**F**). Significant differences between the treatment and control groups are expressed as * *p* < 0.05, ** *p* < 0.01, *** *p* < 0.001 and **** *p* < 0.0001.

BoMac cells (viability >90%) were stimulated with rMbovP274 and rMbovP570 at a dose of 0.375 μM/2 × 10^5^ cells in 1 mL and the relative expression of IL-1, IL-6, IL-8, IL-12, IFN-*γ*, and TNF-α were determined in terms of the fold change relative to the housekeeping gene *β*-actin at 6, 12, and 24 h after treatment. Among the six cytokines tested, only IL-8 was significantly increased by stimulation with rMbovP274 and rMbovP570 (Figure 4). No significant change was observed in the expression of IL-1*β*, TNF-*α*, and IL-6 by either proteins at any time points (data not shown).

The MbovP274 mutant T9.202 (Mut274) and the MbovP570 mutant T9.17 (Mut570) were identified in mutant *M. bovis* library previously constructed in this laboratory. The mutations are located at the genomic positions 315,328 (+) and 672,416 (+), respectively, and at 0.941 and 0.18 of the relative coding sequence (CDS) positions in MbovP274 and MbovP57, respectively.

**Figure 5:**
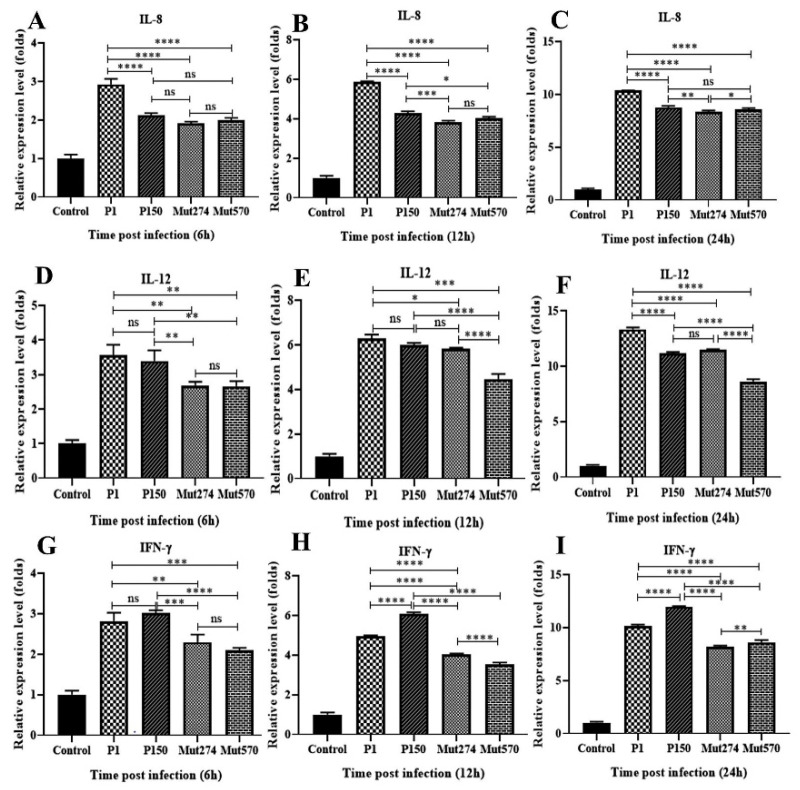
Expression of cytokines in bovine macrophage cells (BoMac) stimulated with *M. bovis* HB0801 (P1) and P150, and mutants Mut274 and Mut570. BoMac cells were infected with one of the four strains or mock infected, and cytokine expression was determined with real-time PCR at 6, 12 and 24 h after infection. IL-8 expression (**A**–**C**), IL-12 expression (**D**–**F**), and interferon *γ* (IFN-*γ*) expression (**G**–**I**) were detected in the infected and uninfected BoMac. The data are expressed as the means ± SE of four independent experiments. Significant differences between the treatment and control groups are expressed as * *p* < 0.05, ** *p* < 0.01, *** *p* < 0.001, and **** *p* < 0.0001.

BoMac cells 2 × 10^5^ cells/mL; viability >90% were infected with the four strains Mut274, Mut570, P1 and P150 at a multiplicity of infection (MOI) of 1000 for 6, 12, and 24 h, and the relative expression of the six cytokines was determined, taking the expression of actin as 1. Compared with the uninfected control, the expression of IL-8, IL-12, and IFN-*γ* was significantly increased in all strains at all three time points. The expression of these three cytokines also differed significantly between the wild-type P1 and the mutants (Mut274 and Mut570) at all three time points. The expression of these three cytokines differed significantly at least one time point after infection with either Mut274 or Mut570 compared with their expression after infection with P150 (Figure 5). There were no significant differences in the expression of IL-1β, TNF-α and IL-6 between the wild types (P1 and P150) and the mutants (Mut274 and Mut570) (data not shown).

## 3. Discussion

Researchers have not only focused on diagnostic tests and treatments for *M. bovis*-associated diseases in the cattle industry but also on developing and producing immunogenic agents to reduce the costs and time lost to the industry. Various strategies have been used to design and develop sufficiently protective new-generation immunogenic agents based on immunoinformatic techniques [18]. Secreted proteins, particularly lipoproteins, are considered to be extremely immunogenic because they are present on the bacterial surface, in the extracellular environment, or on amino-terminal lipoid structures.

We compared the immunogenic proteins differentially secreted in a previously confirmed virulent strain *M. bovis* HB0801-P1, and a derived attenuated and protective strain *M. bovis* P150 [3], using an in silico analysis and experimental validation in vitro.

### 3.1. Twenty-Two Secreted Immunogenic Proteins Identified

From the 438 proteins previously detected with a label-free proteomic analysis, 59 were identified as extracellular and secreted proteins. Based on the high numbers of T-cell epitopes displayed by MHC class I and II molecules and linear and conformational B-cell epitopes predicted, 22 proteins were deemed to be both highly immunogenic and secreted. To confirm the reliability of these results, we examined the corresponding mouse alleles. Although the bovine MHC profile is more complex than the mouse MHC profile, the MHC haplotype sequence homologies in the NCBI database indicate that there is more than 80% identity among the MHC alleles of mice, sheep, and cattle [17]. These results support the strong reliability of our prediction.

Several *M. bovis* HB0801 proteins have previously been shown to be secreted proteins with significant functions. For example, MbovNase induces apoptosis [14]; P27 (MBOV_RS03440) is an immunogenic protein [19]; MbovP579 can be used as a diagnostic biomarker for *M. bovis* [13]; and NADH oxidase is an active enzyme that also functions as an adhesin [20].

Interestingly, 13 previously reported secreted proteins were included in the 59 secreted proteins predicted in the present study with immunoinformatic tools and were classified as either classical or non-classical secreted proteins [21]. Among the final 22 immunogenic secreted proteins selected, MbovP579 [13], MbovNase [14], MbovP217, MbovP674, MbovP739 (confirmed by our laboratory but data not yet reported), MbovP274 and MbovP570 were confirmed in this study, lending support to the reliability of the immunogenic secreted proteins predicted. Most of these proteins (18/22) are lipoproteins, except MbovP580 (nuclease), MbovP471 and MbovP658 (periplasmic protease), and MbovP674 (5′nucleotidase). These data also support the reliability of our predictions.

We demonstrated experimentally that rMbovP274 and rMbovP570 induce high levels of polyclonal antibodies in mouse serum, react very well with the *M. bovis*-positive serum of infected cattle, and induce to the production of cytokines IL-8, IL-12, and IFN-*γ* in BoMac cells. These results confirm that the predicted secreted immunogenic proteins are reliable and should have great utility in the development of novel immunological agents against *M. bovis* infection.

### 3.2. Secreted Proteins Differentially Expressed in Virulent and Attenuated M. bovis Strains

To explain the virulence of wild-type strain *M. bovis* HB0801 and the protective effect afforded by the attenuated vaccine strain *M. bovis* HB0801-P150, we compared the secreted and immunogenic proteins differentially expressed in these two strains.

Among the 59 secreted proteins identified (three in P1, 11 in P150 and 45 in both P1 and P150), the proteins unique to P1 were *VSP* and lipoproteins. The proteins of the *VSP* family are believed to have virulence-associated properties [22]. The proteins unique to P150 were predominantly lipoproteins, but rarely transmembrane lipoproteins. As discussed above, lipoproteins are usually highly antigenic because they occur on the cell surface and contain amino-terminal lipoylated structures [23]. However, among the final 22 immunogenic proteins, only MbovP274 was unique to P1. Although it is highly immunogenic, the conserve domain database (CDD) prediction showed that this protein contains a highly conserved LysR transcriptional regulatory protein family domain that is involved in virulence and biofilm formation [24]. MbovP296 and MbovP518 are unique to P150. MbovP296 contains an S15/NS1/EPRS_RNA-binding domain, which is a homologue of influenza A virus nonstructural immunogenic proteins, and MbovP518 contains an S7 domain, which encodes a viral protease that is responsible for the immunogenicity of viral poly-proteins and their hydrolysis to precursor proteins. The DUF31 putative peptidase domain of this protein is extremely similar to many conserved immunogenic lipoproteins of *M. bovis*. As is well known, lipoproteins can trigger both an innate and acquired immune response. Moreover, lipoprotein-based subunit vaccines can induce an extended memory immune response and high titer of neutralizing antibodies [23]. Therefore, our identification of these immunogenic secreted proteins of *M. bovis* largely explains the differences in virulence of the wild-type (P1) and attenuated vaccine (P150) strains.

### 3.3. MbovP274 and MbovP570 Are Potential Protective Antigens

MbovP274 contains the conserved periplasmic binding protein type-2(PBPT2) domain, which encodes an ion channel domain involved in ion uptake and transportation across cellular compartments and membrane. Its binding ability and topology suggest that this protein is involved in the transportation of immunogenic proteins across the cell membrane. However, MbovP570 has a hypothetical conserved domain of as-yet unknown function. Using recombinant proteins and their mutants, we showed that both proteins induced mouse antibodies, reacted with *M. bovis*-positive cattle serum from infected cattle, and induced six common inflammatory cytokines, including IL-8, IL-12 and IFN-*γ*.

IL-8 is a potent chemoattractant and activator of monocytes, T lymphocytes and neutrophils. It also deploys neutrophils from the bloodstream to the mammary gland [25].

IL-12 is an important immunoregulatory cytokine, which is essentially produced by antigen-presenting cells. The combined expression of IL-12 and IFN-*γ* during infection regulates the innate response, characterizes the Th1 type adaptive immune responses through the IL-12, IFN-*γ* axis, and participates in the clearance of intracellular pathogens. In particular, IFN-*γ* and the acquired immune response increase the Th1 response and thereby play a protective role against *M. bovis* infection. The strong induction of IFN-*γ* by P150 was demonstrated in this study and a previous report [26]. Therefore, MbovP274 and MbovP570 should be valuable in development of novel diagnostic agents and potential vaccines for the control of *M. bovis* infection.

## 4. Materials and Methods

### 4.1. Ethics Statement

The protocols of the mouse experiments in this study were approved by the Experimental Animal Ethics Committee of the Huazhong Agricultural University, Wuhan, China (permit number: HZAUMO-2018-027) and were designed according to the Hubei Regulations for the Administration of Affairs Concerning Experimental Animals.

### 4.2. Secretome Data for M. bovis Strains Used in this Study

*Mycoplasma bovis* HB0801 (China Center for Type Culture Collection CCTCC# M2010040), abbreviated ‘P1’, and its attenuated strain *M. bovis* HB0801-P150 (CCTCC# M2011102), abbreviated ‘P150’, were previously isolated by this laboratory and are kept at the China Center for Type Culture Collection (Wuhan, China). Briefly, both strains were first cultured in PPLO broth for 48 h at 37 °C under 5% CO_2_, harvested by centrifugation [2], suspended in the same volume of sterile phosphate-buffered saline (PBS), and incubated at 37 °C for a further 2 h. The culture supernatants were then collected by centrifugation. After cell debris was removed with a 0.22 μm filter, the supernatant was concentrated 10-folds by ultrafiltration and subjected to a label-free proteomics analysis. The mass spectrometric proteomic data for both strains have been deposited with the ProteomeXchange Consortium via the PRIDE partner repository, under the dataset identifier PXD017700 and then retrieved for this study.

### 4.3. Prediction of Subcellular Localization of the Secretomes

The subcellular localizations of the proteins of the secretomes were predicted with the Bologna Unified Subcellular Component Annotator (BUSCA) server (http://busca.biocomp.unibo.it) and PSORTb 3.0 (http://www.psort.org/psortb/). BUSCA predicts four different compartments for Gram-negative bacteria and three for Gram-positive bacteria, as described previously [27]. PSORTb is the version most frequently used to predict the subcellular localization of proteins [28]. When a signal sequence was detected, the sequence was classified as localized to the extracellular space (GO: 0005615). Alternatively, the protein was processed with ENSEMBLE3.0 to specify whether it was inserted into the plasma membrane (GO: 0005886) or localized in the cytoplasm (GO: 0005737).

### 4.4. Prediction of Protein Secretion and Antigenic Proteins

The secreted or non-secreted nature of *M. bovis* proteins and any signal peptides were predicted. Both classical and non-classical categories were predicted.

SignalP 4.1 (http://www.cbs.dtu.dk/services/SignalP/) was used to identified protein secreted by the classical pathway and presence of signal sequences as described previously [15,29].

Non-classical secretion was predicted with SecretomeP 2.0 (http://www.cbs.dtu.dk/services/SecretomeP/). The classical and non-classical proteins were predicted with default setting score of 0.45 and 0.5, respectively. Therefore, those with scores ≥0.45 and ≥0.5, respectively, were considered secreted proteins [15,30].

PRED-LIPO (http://www.compgen.org/tools/PRED-LIPO) was used to predict the signal peptides in the different datasets, including the experimentally characterized lipoproteins, secretory proteins, proteins with an N-terminal transmembrane domain segment, and cytoplasmic proteins, as described previously [15,31].

The predicted secretory proteins were further analyzed in silico for their antigenicity, to identify the most highly antigenic proteins. The amino acid sequences of all the proteins were submitted to the VaxiJen v2.0 server (http://www.ddg-pharmfac.net/vaxijen/VaxiJen/VaxiJen.html) to distinguish the antigenic and non-antigenic proteins, as described previously [32,33]. Antigenic prediction was adjusted with a default setting score of 0.4, and those proteins with a score ≥0.4 were considered antigenic proteins. This approach was used to select highly antigenic proteins for further analysis.

### 4.5. Prediction of B and T-Cell Epitopes

The B and T-cell epitopes of the antigenic proteins were identified at the IEDB-AR server (https://www.iedb.org/) using the IEDB database [34]. The amino acid sequences of all the proteins were submitted to the IEDB-AR server (https://www.iedb.org/) to predict the linear B-cell epitopes (http://tools.iedb.org/bcell/) with a scoring threshold of ≥18 epitope numbers.

To predict the T-cell epitopes, MHC class I proteins were predicted with the online tools http://tools.iedb.org/main/tcell/ and http://tools.iedb.org/mhci/result/, whereas MHC class II proteins were predicted with the online tool http://tools.iedb.org/mhcii/result/.

T-cell epitopes have been classified according to their binding affinity for mouse MHC alleles using the half-maximal inhibitory concentration (IC_50_) of the biological substance as the unit of measure, with the following criteria: high binding affinity (IC_50_ ˂ 50 nM) or intermediate binding affinity (IC_50_ ˂ 500 nM). The mouse MHC alleles that are used to test antigen peptide binding for MHC class I epitopes are H-2Db, H-2Dd, H-2Kb, H-2Kd, H-2Kk and H-2Ld, using the consensus percentile rank of the top 10% (corresponding to 1000nM) as the unit of measure for high binding affinity (percentile rank ˂ 0.5%) and intermediate binding affinity (percentile rank ˂ 3%). The mouse MHC alleles used to test antigen peptide binding for MHC class II epitopes are H-2IAb, H2IAd and H-2IEd. The binding of the T-cell epitopes of the protein sequences of *M. bovis* P1 and P150 to the mouse MHC molecules was determined with submitting to IEDB-AR in the FASTA format. The nine-mer MHC class I T-cell epitopes were predicted with the artificial neural network method, as described previously [35,36], whereas the 15-mer MHC class II T-cell epitopes were predicted with the consensus method. A combination of the average relative binding (ARB) matrix method and the stabilization matrix alignment method (SMM_align) was finally used [17].

### 4.6. Prediction of Conformational B-Cell Epitopes Based on 3D Structure

The SWISS-MODEL Workspace (https://swissmodel.expasy.org/) was used to 3D model the structural templates and scaffolds based on high-percentage (20–40%) PDB IDs. A template model was obtained for each *M. bovis* proteins by submitting the FASTA-format protein sequences and modeling them.

ElliPro (http://tools.iedb.org/ellipro/) was used to predict the conformational B-cell epitopes of *M. bovis* proteins, using a modeled PDB of the 3D structural templates of each protein. The 3D structural template was chosen based on best-fitting scaffold criteria. The best-fitting template was defined as having a long alignment length, a large number of detection and a small number of gaps. Prediction at a minimum level of 0.5 Å was considered the most moderate, 1.0 Å as the most accurate, and 6.0 Å as the maximum distance for residue clustering. The selection of *M. bovis* proteins carrying candidate B-cell epitopes was based on the number of epitopes predicted with a minimum cut-off score of 0.8 [17].

### 4.7. Cloning and Expression of Immunogenic Candidates Proteins for Validation

From the list of predicted immunogenic proteins, five proteins with 100% sequence identity to those of *M. bovis* HB0801 (GenBank accession number: CP002058.1) and P150 (GenBank accession number: CP007590.1), together with a large number of predicted T- and B-cell epitopes and conserved domains, were selected for experimental validation (Table 3).

The complete genes encoding the five proteins described above were amplified with overlap extension PCR from the genome of *M. bovis* HB0801. All the genes were commercially synthesized after single-nucleotide changes were made at the TGA codons (TGA to TGG) to maintain the correct translation of *M. bovis* tryptophan in *E. coli*. They were then cloned into the pET-30a (+) vector and confirmed commercially with nucleotide sequencing (TIANYI HUIYUAN Company, Wuhan, China). Competent *E. coli* BL21 (DE3) cells (Novagen, USA) were transformed with the recombinant plasmids to express the encoded proteins. Luria–Bertani (LB) broth (5 mL) containing 120 μg/mL kanamycin was inoculated with a single colony of each recombinant strain and incubated in a shaker (37 °C, 180 rpm) until the optical density at a wavelength of 600 nm (OD_600_) reached 0.6. The expression of the recombinant protein was induced with 0.8 mM IPTG in culture for 4 h at 37 °C.

To purify the soluble recombinant proteins, each bacterial lysate was loaded directly onto a nickel affinity chromatography column (GE Healthcare, Sweden) under native conditions, and was eluted with lysis buffer containing 500mM imidazole. The buffer was changed to PBS in a 30 kDa Amicon^®^ Ultra-15 Centrifugal Filter Unit (Millipore), as described previously [14]. The purified proteins were verified with 10% SDS-PAGE.

To purify the insoluble recombinant proteins, we first broke each pellet with inclusion-broken buffer (8 M urea, 0.1 M NaH_2_Po_4_, 0.01 M Tris-Base, pH 8.0). The recombinant proteins were then purified with nickel affinity chromatography, dialyzed against lysis buffer for 24 h, and then treated with the procedures described above.

Lipopolysaccharide (LPS) was removed from the recombinant proteins with polymyxin B resin (Pierce, Sigma), according to the manufacturer’s instructions. Then concentrations of rMbovP274 and rMbovP570 were then determined with a BCA kit (Thermo, Cellchip Biotechnology Company, Waltham, MA, USA).

### 4.8. Production of Polyclonal Antibodies against Recombinant Proteins

Female BLAB/c mice (5 weeks old) were used to produce polyclonal antibodies against the expressed recombinant proteins described above, as described previously [13]. Briefly, 100 μg of purified protein was mixed with an equal volume of Freund’s complete adjuvant (Sigma, Saint Louis, MO, USA) per mouse and used to subcutaneously inoculate two mice. Two subsequent boosters were administered at intervals of 2 weeks, each containing the same amount of protein and an equal volume of Freund’s incomplete adjuvant (Sigma, Saint Louis, MO, USA). One mouse was mock infected as the negative control for each protein. The mice were euthanized and bled when the titer of antibodies directed against each protein reached its peak. The sera were separated and their titers tested with homemade iELISA by coating recombinant proteins, as described previously [13].

Cattle sera were collected previously by this laboratory from nine calves experimentally infected with *M. bovis* (positive) and from the same calves before infection (negative) [26]. These were used to check whether the recombinant proteins in the present study reacted like the native proteins. Pools of sera were prepared for a Western blot assay in this study by mixing the positive and negative serum samples.

### 4.9. Validation of Secretion and Immunogenicity of the Recombinant Proteins

To confirm the secreted proteins with a Western blotting analysis, the secretome of *M. bovis* P1 and P150 and their whole-cell proteins (WCPs) were extracted, as described previously [13]. The secretomes (16 µg/10 µL) and WCPs (8 µg/10 µL) of both strains were separated with 10% SDS-PAGE, and then blotted onto ployvinylidene difluoride (PVDF) membranes. After the membrances were blocked with 5% skimmed milk powder overnight at 4 °C, they were washed with Tris-buffered saline with Tween 20 (TBST). They were then incubated with mouse antiserum (1:2000) against the blotted protein for 2 h at room temperature. After the membranes were washed, they were incubated for 1 h at room temperature with horseradish peroxidase (HRP)-conjugated goat anti-mouse IgG antibody (1:3000) (Southern Biotech, Birmingham, MI, USA). Finally, the protein bands were visualized with SuperSignal^TM^ West Femto Maximum Sensitivity Substrate (Pierce, Thermo Fisher, Waltham, MA, USA), according to the manufacturer’s protocol [20].

Proteins immunogenicity was confirmed with cattle sera. Briefly, each recombinant protein (8 µg in 10 µL) was separated with 10% SDS-PAGE and blotted as described above. The proteins on the membranes were probed with positive and negative pooled cattle sera (1:200) and detected with HRP-conjugated goat anti-bovine IgG antibody (1:5000) (Southern Biotech, Birmingham, MI, USA) for 1 h at room temperature. Finally, the protein bands were visualized with the SuperSignal^TM^ West Femto Maximum Sensitivity Substrate (Pierce, Thermo Fisher, USA), according to the manufacturer’s protocol.

### 4.10. Quantitative PCR (qPCR) Detection of Inflammatory Cytokines Induced by Proteins

The BoMac cell line was kindly supplied by Dr. Judith R. Stable from the Johne’s Disease Research Project, Department of Agriculture in Ames, Iowa, USA, and grown as described previously [37] in RPMI 1640 complete medium with 10% fetal bovine serum (Gibco, Carlsbad, CA, USA). The cells were plated at a concentration of 2 × 10^5^ cells per well in six-well tissue culture plates and incubated for 24 h at 37 °C in a humidified atmosphere with 5% CO_2_. The BoMac cells were treated with purified 0.375 µM rMbovP274 and rMbovP570 for 6, 12, and 24 h and PBS-treated cells were used as the negative control.

BoMac cells were also infected with the mutant MbovP274 and MbovP570 strains, which were identified in a random mutant library constructed and stored in our laboratory, and with *M. bovis* HB0801 -P1 and P150 strains (2 × 10^8^ cells) at an MOI of 1000 at for 6, 12, and 24 h. PBS infected cells were used as the negative control.

Total RNA was extracted from the treated and control cells at the indicated time points using the RNeasy Mini Kit (Qiagen, Valencia, CA, USA), according to the manufacturer’s instructions. Briefly, after the medium was removed entirely from each culture plate, 800 µL of Trizol Reagent (Ambion, Carlsbad, CA, USA) was added to each well to lyse the cells. The lysate was transferred to a 1.5 mL collection tube. The RNA was precipitated and finally dissolved in 30 µL of DNase- and RNase-free water. The concentrations of the RNA samples were determined with a Nano Drop 200 spectrophotometer (Nano Drop, Thermo Fisher, Waltham, MA, USA) and the RNA purity was determined with the absorbance (A) ratio A_260_/A_280_, with values ≥1.8 deemed acceptable.

cDNA was synthesized from the total RNA with reverse transcription using HiScript II Q Select RT SuperMix for qPCR (gDNA Wiper) (Vazyme Biotech Co., Ltd., Nanjing, China), according to the manufacturer’s instructions.

Real-time PCR (qPCR) was used to quantitatively measurer the expression of six gene encoding inflammatory cytokines (IL-1β, 1L-6, 1L-8, 1L-12, TNF-α,and IFN-*γ*), and the β-actin gene (as the internal reference), using the cDNA described above as the template and specific primers (Table 4).

The reaction system had a total volume of 10 µL and contained 8.5 µL of Target Assay Mix (5 µL of 50× Target Assay Mix, 0.2 µL of 50× of Rox II [1× ROX Dye 2], 0.2 µL of 50× both the forward and reverse primers [2 OD], 2.9 µL of 50× the DNase- and RNase-free water), and 1.5 µL of cDNA template (diluted 1:10). The thermal cycling program was 50 °C for 2 min, 95 °C for 5 min, 95 °C for 10 s, and 60 °C for 30 s, followed by 40 cycles of 95 °C for 15 s and 60 °C for 1 min.

The comparative Ct method was applied to the qPCR data. The values were normalized to β-actin by subtracting the mean β-actin Ct from the mean target Ct for each sample, to give the ΔCt. Relative transcription quantification uses the 2ΔΔCt generated by subtracting the mean ΔCt of untreated samples from those of the treated samples. The 2ΔΔCt equation calculates the fold increase in gene expression in the treated samples relative to the untreated samples (calibrator). The data obtained from the qPCR reactions were log-transformed and compared using one-way ANOVA with GraphPad Prism version 8.2 (GraphPad Software, La Jolla, CA, USA). * *p* < 0.05, ** *p* < 0.01, *** *p* < 0.001, and **** *p* < 0.0001 were considered significant in figures.

## 5. Conclusions

Using various immunoinformatic strategies, 22 secreted immunological *M. bovis* proteins were identified from 438 proteins that had been identified previously with a label-free proteomic approach, including one unique to P1, two unique to P150 and 19 common to both. MbovP274 (in P1) and MbovP570 (in both) were experimentally confirmed to be secreted immunological proteins that induced IL-8, IL-12 and IFN-*γ*. These results will have great utility in the development of novel immunological agents against *M. bovis* infection.

## Figures and Tables

**Table 1:** In silico identification of subcellular localization, secretion and antigenicity of *M. bovis* proteins.

No.	Proteins ^1^					Signal Peptide ^2^	Classical ^3^	Non Classical ^4^	Antigenic/Non-Antigenic ^5^	
	Mnemonic	Accession No.	GO Ids	GO Terms	Score				Overall Protective Antigen Prediction Score	Antigen/Non-Antigen
1	Mbov_0016 ^a,b^	AFM51394_1	GO:0005615	Extracellular	0.92	L	Y	Y	0.5093	A
2	Mbov_0037 ^a,b^	AFM51415_2	GO:0005615	Extracellular	0.87	L	Y	Y	0.3970	NA
3	Mbov_0038 ^a,b^	AFM51416_1	GO:0005615	Extracellular	0.73	T	N	Y	0.4219	A
4	Mbov_0049 ^a,b^	AFM51424_1	GO:0005615	Extracellular	0.88	L	Y	Y	0.5480	A
5	Mbov_0111 ^a,b^	AFM51486_1	GO:0005615	Extracellular	0.77	L	Y	Y	0.5117	A
6	Mbov_0154 ^a,b^	AFM51527_1	GO:0005615	Extracellular	0.96	T	N	Y	0.5622	A
7	Mbov_0156 ^a,b^	AFM51529_1	GO:0005615	Extracellular	0.92	L	Y	Y	0.5078	A
8	Mbov_0211 ^b^	AFM51580_1	GO:0005615	Extracellular	0.96	L	Y	Y	0.3222	NA
9	Mbov_0217 ^a,b^	AFM51586_1	GO:0005615	Extracellular	0.96	L	Y	Y	0.4783	A
10	Mbov_0274 ^a^	AFM51642_1	GO:0005615	Extracellular	0.91	L	Y	Y	0.5003	A
11	Mbov_0283 ^b^	AFM51651_1	GO:0005615	Extracellular	0.96	S	Y	Y	0.9083	A
12	Mbov_0290 ^b^	AFM51658_1	GO:0005615	Extracellular	0.91	L	Y	Y	0.4128	A
13	Mbov_0296 ^b^	AFM51664_1	GO:0005615	Extracellular	0.96	S	Y	Y	0.4878	A
14	Mbov_0326 ^b^	AFM51694_1	GO:0005615	Extracellular	0.96	L	Y	Y	0.3961	NA
15	Mbov_0339 ^b^	AFM51707_1	GO:0005615	Extracellular	0.91	L	Y	Y	1.2671	A
16	Mbov_0341 ^b^	AFM51709_1	GO:0005615	Extracellular	0.91	S	Y	Y	0.2139	NA
17	Mbov_0350 ^a,b^	AFM51716_1	GO:0005615	Extracellular	0.97	S	Y	Y	0.4786	A
18	Mbov_0364 ^a,b^	AFM51726_1	GO:0005615	Extracellular	0.96	S	Y	Y	0.4827	A
19	Mbov_0368 ^b^	AFM51730_1	GO:0005615	Extracellular	0.97	S	Y	Y	0.5245	A
20	Mbov_0374 ^b^	AFM51736_1	GO:0005615	Extracellular	0.97	L	Y	Y	0.4516	A
21	Mbov_0393 ^a,b^	AFM51750_1	GO:0005615	Extracellular	0.96	T	Y	Y	0.3484	NA
22	Mbov_0449 ^a,b^	AFM51806_2	GO:0005615	Extracellular	0.95	S	Y	Y	1.0254	A
23	Mbov_0458 ^a,b^	AFM51815_1	GO:0005615	Extracellular	0.96	L	Y	Y	1.0635	A
24	Mbov_0461 ^a,b^	AFM51818_1	GO:0005615	Extracellular	0.96	S	Y	Y	0.5130	A
25	Mbov_0462 ^a,b^	AFM51819_1	GO:0005615	Extracellular	0.97	L	Y	Y	1.1146	A
26	Mbov_0467 ^b^	AFM51823_1	GO:0005615	Extracellular	0.93	T	N	Y	0.3328	NA
27	Mbov_0468 ^a,b^	AFM51824_1	GO:0005615	Extracellular	0.97	L	Y	Y	0.4890	A
28	Mbov_0469 ^a,b^	AFM51825_1	GO:0005615	Extracellular	0.97	L	Y	Y	1.0196	A
29	Mbov_0471 ^a,b^	AFM51827_1	GO:0005615	Extracellular	0.92	S	Y	Y	0.5445	A
30	Mbov_0473 ^a,b^	AFM51829_1	GO:0005615	Extracellular	0.91	L	Y	Y	0.6593	A
31	Mbov_0505 ^a,b^	AFM51861_1	GO:0005615	Extracellular	0.78	S	Y	Y	0.6022	A
32	Mbov_0515 ^a,b^	AFM51869_1	GO:0005615	Extracellular	0.97	L	Y	Y	0.5203	A
33	Mbov_0516 ^a,b^	AFM51870_1	GO:0005615	Extracellular	0.96	S	Y	Y	0.4863	A
34	Mbov_0517 ^a,b^	AFM51871_1	GO:0005615	Extracellular	0.95	S	Y	Y	0.4955	A
35	Mbov_0518 ^b^	AFM51872_1	GO:0005615	Extracellular	0.96	S	Y	Y	0.5014	A
36	Mbov_0519 ^a,b^	AFM51873_1	GO:0005615	Extracellular	0.79	T	Y	Y	0.4463	A
37	Mbov_0536 ^a,b^	AFM51890_1	GO:0005615	Extracellular	0.87	L	Y	Y	0.4537	A
38	Mbov_0537 ^a,b^	AFM51891_1	GO:0005615	Extracellular	0.93	L	Y	Y	0.4844	A
39	Mbov_0570 ^a,b^	AFM51924_1	GO:0005615	Extracellular	0.9	L	Y	Y	0.5094	A
40	Mbov_0579 ^a,b^	AFM51933_1	GO:0005615	Extracellular	0.96	L	Y	Y	0.4661	A
41	Mbov_0580 ^a,b^	AFM51934_1	GO:0005615	Extracellular	0.97	S	Y	Y	0.4848	A
42	Mbov_0585 ^a,b^	AFM51939_1	GO:0005615	Extracellular	0.96	S	Y	Y	0.4742	A
43	Mbov_0654 ^a,b^	AFM52005_1	GO:0005615	Extracellular	0.8	L	Y	Y	0.8413	A
44	Mbov_0656 ^a,b^	AFM52007_1	GO:0005615	Extracellular	0.8	NS	N	Y	1.0586	A
45	Mbov_0658 ^a,b^	AFM52009_1	GO:0005615	Extracellular	0.92	S	Y	Y	0.4564	A
46	Mbov_0674 ^a,b^	AFM52024_1	GO:0005615	Extracellular	0.87	T	Y	Y	0.5553	A
47	Mbov_0675 ^a,b^	AFM52025_1	GO:0005615	Extracellular	0.97	L	Y	Y	0.5009	A
48	Mbov_0693 ^a,b^	AFM52042_1	GO:0005615	Extracellular	0.95	T	N	Y	0.2052	NA
49	Mbov_0696 ^a,b^	AFM52045_1	GO:0005615	Extracellular	0.96	S	Y	Y	0.8248	A
50	Mbov_0732 ^a^	AFM52081_1	GO:0005737	Extracellular	8.91	NS	N	Y	0.2937	NA
51	Mbov_0739 ^a,b^	AFM52087_1	GO:0005615	Extracellular	0.96	L	Y	Y	0.5234	A
52	Mbov_0743 ^a,b^	AFM52091_1	GO:0005615	Extracellular	0.82	T	N	Y	0.5336	A
53	Mbov_0768 ^a,b^	AFM52116_1	GO:0005615	Extracellular	0.97	S	Y	Y	0.5745	A
54	Mbov_0793 ^a,b^	AFM52141_1	GO:0005615	Extracellular	0.9	L	Y	Y	1.1056	A
55	Mbov_0794 ^a,b^	AFM52142_1	GO:0005615	Extracellular	0.9	L	Y	Y	0.8247	A
56	Mbov_0795 ^a^	AFM52143_1	GO:0005615	Extracellular	0.93	L	Y	Y	0.3425	NA
57	Mbov_0796 ^a,b^	AFM52144_1	GO:0005615	Extracellular	0.95	L	Y	Y	0.8931	A
58	Mbov_0797 ^a,b^	AFM52145_1	GO:0005615	Extracellular	0.96	L	Y	Y	1.1257	A
59	Mbov_0798 ^a,b^	AFM52146_1	GO:0005615	Extracellular	0.96	L	Y	Y	0.8682	A

^1^ Protein subcellular localization predicted by BUACA and PSORTb 3.0. ^2^ Protein signal peptide predicted by PRED-LIPO, L: Lipo signal peptide, S: Sec signal peptide, T: TM segment, and NS: Non-Signal peptide. ^3^ Prediction of Classical proteins, Y: Yes, it is classical and N: No, it is not classical. ^4^ Prediction of Non-Classical proteins, Y: Yes, it is Non- Classical proteins. ^5^ Prediction of Antigenic/Non-Antigenic proteins, A: Antigenic and NA: Non-Antigenic. ^a^: present in *M. bovis* HB0801-P1, ^b^: present in P150 and ^a,b^: present in both (*M. bovis* HB0801-P1and P150).

**Table 2:** Identification of linear B-cell epitopes and overlapping residues in MHC class I and II T-cell epitopes.

No.	Proteins			Epitopes		
	Mnemonic	Accession No.	Protein	Over All B Cell Epitopes Numbers	B-Cells	T-Cell (MHC Class I & II)
1	Mbov_0016 ^a,b^	AFM51394_1	P48-like surface lipoprotein	22	+	+(I,II)
2	Mbov_0038 ^a,b^	AFM51416_1	putative transmembrane protein	161	+	+(I,II)
3	Mbov_0049 ^a,b^	AFM51424_1	putative lipoprotein	34	+	+(I,II)
4	Mbov_0111 ^a,b^	AFM51486_1	putative lipoprotein	53	+	+(I,II)
5	Mbov_0154 ^a,b^	AFM51527_1	putative transmembrane protein	24	+	+(I,II)
6	Mbov_0217 ^a,b^	AFM51586_1	putative lipoprotein	21	+	+(I,II)
7	Mbov_0274 ^a^	AFM51642_1	putative lipoprotein	35	+	+(I,II)
8	Mbov_0290 ^b^	AFM51658_1	putative lipoprotein	26	+	- (I,II)
9	Mbov_0296 ^b^	AFM51664_1	putative lipoprotein	30	+	+(I,II)
10	Mbov_0350 ^a,b^	AFM51716_1	putative lipoprotein	36	+	- (I,II)
11	Mbov_0364 ^a,b^	AFM51726_1	putative membrane lipoprotein	25	+	- (I,II)
12	Mbov_0374 ^b^	AFM51736_1	putative lipoprotein	38	+	- (I,II)
13	Mbov_0468 ^a,b^	AFM51824_1	putative lipoprotein	24	+	+(I,II)
14	Mbov_0471 ^a,b^	AFM51827_1	Periplasmic protease	30	+	+(I,II)
15	Mbov_0505 ^a,b^	AFM51861_1	putative lipoprotein	38	+	+(I,II)
16	Mbov_0515 ^a,b^	AFM51869_1	putative lipoprotein	52	+	+(I,II)
17	Mbov_0516 ^a,b^	AFM51870_1	Putative transmembrane protein	46	+	+(I,II)
18	Mbov_0517 ^a,b^	AFM51871_1	Putative transmembrane protein	42	+	+(I,II)
19	Mbov_0518 ^b^	AFM51872_1	putative lipoprotein	45	+	+(I,II)
20	Mbov_0519 ^a,b^	AFM51873_1	Putative transmembrane protein	41	+	+(I,II)
21	Mbov_0536 ^a,b^	AFM51890_1	putative lipoprotein	19	+	+(I,II)
22	Mbov_0570 ^a,b^	AFM51924_1	putative lipoprotein	34	+	+(I,II)
23	Mbov_0579 ^a,b^	AFM51933_1	membrane lipoprotein P81	39	+	+(I,II)
24	Mbov_0580 ^a,b^	AFM51934_1	nuclease	18	+	+(I,II)
25	Mbov_0585 ^a,b^	AFM51939_1	putative lipoprotein	18	+	+(I,II)
26	Mbov_0658 ^a,b^	AFM52009_1	Periplasmic protease	29	+	+(I,II)
27	Mbov_0674 ^a,b^	AFM52024_1	putative lipoprotein	18	+	+(I,II)
28	Mbov_0675 ^a,b^	AFM52025_1	5′nucleotidase	36	+	+(I,II)
29	Mbov_0739 ^a,b^	AFM52087_1	putative lipoprotein	38	+	+(I,II)
30	Mbov_0743 ^a,b^	AFM52091_1	putative transmembrane protein	48	+	+(I,II)
31	Mbov_0798 ^a,b^	AFM52146_1	variable surface lipoprotein VspHB0801-6	22	+	+(I,II)

^a^: present in *M. bovis* HB0801-P1, ^b^: present in P150 and ^a,b^: present in both (*M. bovis* HB0801-P1 and P150).

**Table 3:** Selection of immunogenic secreted *M. bovis* proteins with conserved domains.

Antigenic Proteins of *M. bovis* HB0801 with NCBI Protein ID	Protein Name	Domains	Name	Accession No.
Putative lipoprotein (MbovP049 ^a,b^) AFM51424_1	Putative lipoprotein	330-636	SMC_N super family	cl25732
Putative lipoprotein (MbovP274 ^a^) AFM51642_1	Putative lipoprotein	1-267	UgpB super family	cl25886
Putative lipoprotein (MbovP296 ^b^) AFM51664_1	Putative lipoprotein	450-576	Peptidase_S41 super family	cl02526
Putative lipoprotein (MbovP570 ^a,b^) AFM51924_1	Putative lipoprotein	458-624	HemL super family	cl28400
putative lipoprotein (MbovP585 ^a,b^) AFM51939_1	Putative lipoprotein	364-499	SMC_N super family	cl25732

^a^: present in *M. bovis* HB0801-P1, ^b^: present in P150 and ^a,b^: present in both (*M. bovis* HB0801-P1 and P150).

**Table 4:** Sequences of oligonucleotide primers used in this study.

Gene Name	Primer Sequence (5′-3′)	Amplicon Size (bp)	Accession Number	Annealing Temperature (°C)	References
IL-1β	F: GTCATCTTCGAAACGTCCTCC R: TCCTCTCCTTGCACAAAGCTC	191	M37211	60	[14]
IL-6	F: ACCCCAGGCAGACTACTTCT R: CCCAGATTGGAAGCATCCGT	195	NM173923.2	60	[14]
IL-12	F: GCTTGGAGCACAGGGAGTAT R: AGTTGCAGGTTCTTGGGTGG	151	NM174356.1	60	[14]
TNF-∝	F: CTCCATCAACAGCCCTCTGG R: GAGGGCATTGGCATACGAGT	136	NM173966	60	[14]
IFN-*γ*	F: TCAAATTCCGGTGGATGATCTGC R: GACCATTACGTTGATGCTCTCCG	150	NM 174086.1	60	[38]
IL-8	F: GAAGAGAGCTGAGAAGCAAGATCC R: ACCCACACAGAACATGAGGC	142	NM173925.2	60	[14]
β-Actin	F: AGCAAGCAGGAGTACGATGAG R: ATCCAACCGACTGCTGTCA	241	NM 173979.3	60	[14]

F: Forward, R: Reverse.

## Supplementary Materials

The following are available online at https://www.mdpi.com/2076-0817/9/9/770/s1, Table S1: Subcellular localization of *M. bovis* proteins identified by using BUSCA server, Table S2: Subcellular localization of *M. bovis* proteins identified by using PSORTb 3.0 server, Table S3: A total of 59 extracellular secreted *M. bovis* proteins identified by using PRID-LIPO signal peptide, Table S4: Identification of linear B-cell epitopes of *M. bovis* secreted proteins, Table S5: Identification the affinity of T-cell epitopes to MHC class I (nine-mers) of *M. bovis* secreted proteins by using IEDB-AR, Table S6: Identification the affinity of T-cell epitopes to MHC class II (15-mers) of *M. bovis* secreted proteins by using IEDB-AR, Table S7: Overlapped MHC class I and II T-cell epitopes of *M. bovis* secreted antigenic proteins identified by using IEDB-AR, Table S8: The 3D structure templates used for recognition of conformational B-cell epitopes at minimum cut-off 0.8 for *M. bovis* secreted proteins.
