# Supplementary material for: In Silico Identification of Novel Immunogenic Secreted Proteins of Mycoplasma bovis from Secretome Data and Experimental Verification
Source: Pathogens. 2020 Sep 21;9(9):770. doi: 10.3390/pathogens9090770 (PMC7559824; doi:10.3390/pathogens9090770)
Supplement: Supplementary file 1 [file pathogens-09-00770-s001.pdf]

Table S1: Subcellular localization of *M. bovis* proteins predicted by using BUSCA server.

No	Protein ID	Accession No	GO ids	GO terms	Score
1	Mbov_0001[a]	AFM51379.1	GO:0005737	C:cytoplasm	0.7
2	Mbov_0002[a,b]	AFM51380.1	GO:0005737	C:cytoplasm	0.7
3	Mbov_0003[a]	AFM51381.1	GO:0005737	C:cytoplasm	0.7
4	Mbov_0004[a,b]	AFM51382.1	GO:0005737	C:cytoplasm	0.7
5	Mbov_0006[a,b]	AFM51384.1	GO:0005737	C:cytoplasm	0.7
6	Mbov_0007[a,b]	AFM51385.1	GO:0005737	C:cytoplasm	0.7
7	Mbov_0008[a,b]	AFM51386.1	GO:0005737	C:cytoplasm	0.7
8	Mbov_0009[a,b]	AFM51387.1	GO:0005737	C:cytoplasm	0.7
9	Mbov_0010[a,b]	AFM51388.1	GO:0005737	C:cytoplasm	0.7
10	Mbov_0011[a,b]	AFM51389.1	GO:0005737	C:cytoplasm	0.7
11	Mbov_0012[a,b]	AFM51390.1	GO:0005737	C:cytoplasm	0.7
12	Mbov_0016[a,b]	AFM51394.1	GO:0005615	C:extracellular space	0.92
13	Mbov_0018[a,b]	AFM51396.1	GO:0005737	C:cytoplasm	0.7
14	Mbov_0022[a,b]	AFM51400.1	GO:0005737	C:cytoplasm	0.7
15	Mbov_0023[a,b]	AFM51401.1	GO:0005737	C:cytoplasm	0.7
16	Mbov_0025[a,b]	AFM51403.1	GO:0005737	C:cytoplasm	0.7
17	Mbov_0026[a,b]	AFM51404.1	GO:0005737	C:cytoplasm	0.7
18	Mbov_0027[a]	AFM51405.1	GO:0005737	C:cytoplasm	0.7
19	Mbov_0037[a,b]	AFM51415.2	GO:0005615	C:extracellular space	0.87
20	Mbov_0038[a,b]	AFM51416.1	GO:0005615	C:extracellular space	0.73
21	Mbov_0044[a]	AFM51419.1	GO:0005737	C:cytoplasm	0.7
22	Mbov_0048[a,b]	AFM51423.1	GO:0005737	C:cytoplasm	0.7
23	Mbov_0049[a,b]	AFM51424.1	GO:0005615	C:extracellular space	0.88
24	Mbov_0051[a,b]	AFM51426.1	GO:0005737	C:cytoplasm	0.7
25	Mbov_0052[a,b]	AFM51427.1	GO:0005737	C:cytoplasm	0.7
26	Mbov_0056[a,b]	AFM51431.1	GO:0005737	C:cytoplasm	0.7
27	Mbov_0058[a]	AFM51433.1	GO:0005737	C:cytoplasm	0.7
28	Mbov_0059[a,b]	AFM51434.1	GO:0005737	C:cytoplasm	0.7
29	Mbov_0062[a,b]	AFM51437.1	GO:0005737	C:cytoplasm	0.7
30	Mbov_0063[a,b]	AFM51438.1	GO:0005737	C:cytoplasm	0.7
31	Mbov_0068[a,b]	AFM51443.1	GO:0005737	C:cytoplasm	0.7

32	Mbov_0069[a,b]	AFM51444.1	GO:0005737	C:cytoplasm	0.7
33	Mbov_0071[a,b]	AFM51446.1	GO:0005737	C:cytoplasm	0.7
34	Mbov_0072[a,b]	AFM51447.1	GO:0005886	C:plasma membrane	0.63
35	Mbov_0074[a,b]	AFM51449.1	GO:0005737	C:cytoplasm	0.7
36	Mbov_0075[a]	AFM51450.1	GO:0005737	C:cytoplasm	0.7
37	Mbov_0076[a,b]	AFM51451.1	GO:0005737	C:cytoplasm	0.7
38	Mbov_0077[a,b]	AFM51452.1	GO:0005737	C:cytoplasm	0.7
39	Mbov_0078[a,b]	AFM51453.1	GO:0005737	C:cytoplasm	0.7
40	Mbov_0079[a]	AFM51454.1	GO:0005737	C:cytoplasm	0.7
41	Mbov_0080[a,b]	AFM51455.1	GO:0005737	C:cytoplasm	0.7
42	Mbov_0082[a,b]	AFM51457.1	GO:0005886	C:plasma membrane	0.48
43	Mbov_0083[a,b]	AFM51458.1	GO:0005737	C:cytoplasm	0.7
44	Mbov_0085[a,b]	AFM51460.1	GO:0005737	C:cytoplasm	0.7
45	Mbov_0086[a,b]	AFM51461.1	GO:0005737	C:cytoplasm	0.7
46	Mbov_0088[a,b]	AFM51463.1	GO:0005737	C:cytoplasm	0.7
47	Mbov_0089[a,b]	AFM51464.1	GO:0005737	C:cytoplasm	0.7
48	Mbov_0093[a]	AFM51468.1	GO:0005737	C:cytoplasm	0.7
49	Mbov_0094[a,b]	AFM51469.1	GO:0005737	C:cytoplasm	0.7
50	Mbov_0095[a]	AFM51470.1	GO:0005737	C:cytoplasm	0.7
51	Mbov_0096[a]	AFM51471.1	GO:0005737	C:cytoplasm	0.7
52	Mbov_0098[a,b]	AFM51473.1	GO:0005737	C:cytoplasm	0.7
53	Mbov_0100[a,b]	AFM51475.1	GO:0005737	C:cytoplasm	0.7
54	Mbov_0102[a,b]	AFM51477.1	GO:0005737	C:cytoplasm	0.7
55	Mbov_0103[a,b]	AFM51478.1	GO:0005737	C:cytoplasm	0.7
56	Mbov_0104[a,b]	AFM51479.1	GO:0005737	C:cytoplasm	0.7
57	Mbov_0105[a,b]	AFM51480.1	GO:0005737	C:cytoplasm	0.7
58	Mbov_0106[a,b]	AFM51481.1	GO:0005737	C:cytoplasm	0.7
59	Mbov_0107[a,b]	AFM51482.1	GO:0005737	C:cytoplasm	0.7
60	Mbov_0109[a,b]	AFM51484.1	GO:0005737	C:cytoplasm	0.7
61	Mbov_0111[a,b]	AFM51486.1	GO:0005615	C:extracellular space	0.77
62	Mbov_0114[a,b]	AFM51489.1	GO:0005737	C:cytoplasm	0.7
63	Mbov_0115[a,b]	AFM51490.1	GO:0005737	C:cytoplasm	0.7
64	Mbov_0120[a,b]	AFM51495.2	GO:0005737	C:cytoplasm	0.7
65	Mbov_0123[a,b]	AFM51498.2	GO:0005737	C:cytoplasm	0.7

66	Mbov_0124[a,b]	AFM51499.2	GO:0005737	C:cytoplasm	0.7
67	Mbov_0125[a,b]	AFM51500.1	GO:0005737	C:cytoplasm	0.7
68	Mbov_0126[a,b]	AFM51501.1	GO:0005737	C:cytoplasm	0.7
69	Mbov_0127[a,b]	AFM51502.1	GO:0005737	C:cytoplasm	0.7
70	Mbov_0131[a,b]	AFM51506.1	GO:0005737	C:cytoplasm	0.7
71	Mbov_0133[a,b]	AFM51508.1	GO:0005737	C:cytoplasm	0.7
72	Mbov_0139[a]	AFM51514.1	GO:0005737	C:cytoplasm	0.7
73	Mbov_0141[a]	AFM51516.1	GO:0005737	C:cytoplasm	0.7
74	Mbov_0145[a]	AFM51518.1	GO:0005737	C:cytoplasm	0.7
75	Mbov_0148[a,b]	AFM51521.1	GO:0005886	C:plasma membrane	0.62
76	Mbov_0149[a,b]	AFM51522.1	GO:0005737	C:cytoplasm	0.7
77	Mbov_0150[a,b]	AFM51523.1	GO:0005737	C:cytoplasm	0.7
78	Mbov_0151[a,b]	AFM51524.1	GO:0005737	C:cytoplasm	0.7
79	Mbov_0152[a,b]	AFM51525.1	GO:0005737	C:cytoplasm	0.7
80	Mbov_0153[a,b]	AFM51526.1	GO:0005737	C:cytoplasm	0.7
81	Mbov_0154[a,b]	AFM51527.1	GO:0005615	C:extracellular space	0.96
82	Mbov_0155[a,b]	AFM51528.1	GO:0005737	C:cytoplasm	0.7
83	Mbov_0156[a,b]	AFM51529.1	GO:0005615	C:extracellular space	0.92
84	Mbov_0157[a,b]	AFM51530.1	GO:0005737	C:cytoplasm	0.7
85	Mbov_0158[a]	AFM51531.1	GO:0005737	C:cytoplasm	0.7
86	Mbov_0160[a,b]	AFM51533.1	GO:0005737	C:cytoplasm	0.7
87	Mbov_0165[a,b]	AFM51536.1	GO:0005737	C:cytoplasm	0.7
88	Mbov_0168[a,b]	AFM51537.1	GO:0005737	C:cytoplasm	0.7
89	Mbov_0174[a,b]	AFM51543.1	GO:0005737	C:cytoplasm	0.7
90	Mbov_0175[a,b]	AFM51544.1	GO:0005737	C:cytoplasm	0.7
91	Mbov_0176[a,b]	AFM51545.1	GO:0005737	C:cytoplasm	0.7
92	Mbov_0190[a,b]	AFM51559.2	GO:0005886	C:plasma membrane	0.84
93	Mbov_0191[a,b]	AFM51560.1	GO:0005737	C:cytoplasm	0.7
94	Mbov_0192[a,b]	AFM51561.1	GO:0005737	C:cytoplasm	0.7
95	Mbov_0196[a,b]	AFM51565.1	GO:0005737	C:cytoplasm	0.7
96	Mbov_0197[a,b]	AFM51566.1	GO:0005886	C:plasma membrane	0.8
97	Mbov_0198[a,b]	AFM51567.1	GO:0005737	C:cytoplasm	0.7
98	Mbov_0199[a]	AFM51568.1	GO:0005737	C:cytoplasm	0.7
99	Mbov_0200[a,b]	AFM51569.1	GO:0005737	C:cytoplasm	0.7

100	Mbov_0202[a,b]	AFM51571.1	GO:0005737	C:cytoplasm	0.7
101	Mbov_0203[a,b]	AFM51572.1	GO:0005737	C:cytoplasm	0.7
102	Mbov_0205[a,b]	AFM51574.1	GO:0005737	C:cytoplasm	0.7
103	Mbov_0206[a,b]	AFM51575.1	GO:0005737	C:cytoplasm	0.7
104	Mbov_0207[a]	AFM51576.1	GO:0005737	C:cytoplasm	0.7
105	Mbov_0210[a]	AFM51579.1	GO:0005737	C:cytoplasm	0.7
106	Mbov_0211[b]	AFM51580.1	GO:0005615	C:extracellular space	0.96
107	Mbov_0212[a,b]	AFM51581.1	GO:0005737	C:cytoplasm	0.7
108	Mbov_0213[a,b]	AFM51582.1	GO:0005737	C:cytoplasm	0.7
109	Mbov_0214[a,b]	AFM51583.1	GO:0005737	C:cytoplasm	0.7
110	Mbov_0216[a,b]	AFM51585.1	GO:0005737	C:cytoplasm	0.7
111	Mbov_0217[a,b]	AFM51586.1	GO:0005615	C:extracellular space	0.96
112	Mbov_0218[a,b]	AFM51587.1	GO:0005737	C:cytoplasm	0.7
113	Mbov_0220[a,b]	AFM51589.1	GO:0005737	C:cytoplasm	0.7
114	Mbov_0222[a,b]	AFM51591.1	GO:0005737	C:cytoplasm	0.7
115	Mbov_0223[a]	AFM51592.2	GO:0005737	C:cytoplasm	0.7
116	Mbov_0224[a,b]	AFM51593.1	GO:0005737	C:cytoplasm	0.7
117	Mbov_0225[a,b]	AFM51594.1	GO:0005737	C:cytoplasm	0.7
118	Mbov_0226[a,b]	AFM51595.1	GO:0005737	C:cytoplasm	0.7
119	Mbov_0227[a,b]	AFM51596.1	GO:0005737	C:cytoplasm	0.7
120	Mbov_0228[a,b]	AFM51597.1	GO:0005737	C:cytoplasm	0.7
121	Mbov_0231[a,b]	AFM51600.1	GO:0005737	C:cytoplasm	0.7
122	Mbov_0232[a]	AFM51601.1	GO:0005737	C:cytoplasm	0.7
123	Mbov_0235[a,b]	AFM51604.1	GO:0005737	C:cytoplasm	0.7
124	Mbov_0237[a,b]	AFM51606.1	GO:0005737	C:cytoplasm	0.7
125	Mbov_0239[a]	AFM51608.1	GO:0005737	C:cytoplasm	0.7
126	Mbov_0242[a,b]	AFM51611.1	GO:0005737	C:cytoplasm	0.7
127	Mbov_0243[a,b]	AFM51612.1	GO:0005737	C:cytoplasm	0.7
128	Mbov_0244[a,b]	AFM51613.1	GO:0005737	C:cytoplasm	0.7
129	Mbov_0247[a,b]	AFM51616.1	GO:0005737	C:cytoplasm	0.7
130	Mbov_0255[a,b]	AFM51624.1	GO:0005737	C:cytoplasm	0.7
131	Mbov_0256[a,b]	AFM51625.1	GO:0005737	C:cytoplasm	0.7
132	Mbov_0266[a,b]	AFM51635.1	GO:0005737	C:cytoplasm	0.7
133	Mbov_0267[a,b]	AFM51636.1	GO:0005737	C:cytoplasm	0.7

134	Mbov_0268[a,b]	AFM51637.1	GO:0005737	C:cytoplasm	0.7
135	Mbov_0269[a]	AFM51638.1	GO:0005737	C:cytoplasm	0.7
136	Mbov_0270[a,b]	AFM51639.1	GO:0005737	C:cytoplasm	0.7
137	Mbov_0274 [a]	AFM51642.1	GO:0005615	C:extracellular space	0.91
138	Mbov_0277[a,b]	AFM51645.1	GO:0005737	C:cytoplasm	0.7
139	Mbov_0278[a,b]	AFM51646.1	GO:0005737	C:cytoplasm	0.7
140	Mbov_0283[b]	AFM51651.1	GO:0005615	C:extracellular space	0.96
141	Mbov_0286[a,b]	AFM51654.1	GO:0005737	C:cytoplasm	0.7
142	Mbov_0290[b]	AFM51658.1	GO:0005615	C:extracellular space	0.91
143	Mbov_0294[a,b]	AFM51662.1	GO:0005737	C:cytoplasm	0.7
144	Mbov_0295[a,b]	AFM51663.1	GO:0005737	C:cytoplasm	0.7
145	Mbov_0296[b]	AFM51664.1	GO:0005615	C:extracellular space	0.96
146	Mbov_0297[a,b]	AFM51665.2	GO:0005737	C:cytoplasm	0.7
147	Mbov_0299[a,b]	AFM51667.1	GO:0005737	C:cytoplasm	0.7
148	Mbov_0301[a,b]	AFM51669.1	GO:0005737	C:cytoplasm	0.7
149	Mbov_0302[a,b]	AFM51670.1	GO:0005737	C:cytoplasm	0.7
150	Mbov_0303[a,b]	AFM51671.1	GO:0005737	C:cytoplasm	0.7
151	Mbov_0304[a,b]	AFM51672.1	GO:0005737	C:cytoplasm	0.7
152	Mbov_0305[a,b]	AFM51673.1	GO:0005886	C:plasma membrane	0.8
153	Mbov_0309[a,b]	AFM51677.1	GO:0005737	C:cytoplasm	0.7
154	Mbov_0310[a,b]	AFM51678.1	GO:0005737	C:cytoplasm	0.7
155	Mbov_0312[a,b]	AFM51680.1	GO:0005737	C:cytoplasm	0.7
156	Mbov_0313[a,b]	AFM51681.1	GO:0005737	C:cytoplasm	0.7
157	Mbov_0314[a,b]	AFM51682.1	GO:0005737	C:cytoplasm	0.7
158	Mbov_0315[a,b]	AFM51683.1	GO:0005737	C:cytoplasm	0.7
159	Mbov_0316[a]	AFM51684.1	GO:0005737	C:cytoplasm	0.7
160	Mbov_0319[a,b]	AFM51687.1	GO:0005737	C:cytoplasm	0.7
161	Mbov_0321[a,b]	AFM51689.1	GO:0005737	C:cytoplasm	0.7
162	Mbov_0322[a,b]	AFM51690.1	GO:0005737	C:cytoplasm	0.7
163	Mbov_0325[a,b]	AFM51693.1	GO:0005737	C:cytoplasm	0.7
164	Mbov_0326[b]	AFM51694.1	GO:0005615	C:extracellular space	0.96
165	Mbov_0327[a,b]	AFM51695.1	GO:0005886	C:plasma membrane	0.56
166	Mbov_0328[a,b]	AFM51696.1	GO:0005737	C:cytoplasm	0.7
167	Mbov_0330[a,b]	AFM51698.1	GO:0005737	C:cytoplasm	0.7

168	Mbov_0331[a,b]	AFM51699.1	GO:0005737	C:cytoplasm	0.7
169	Mbov_0332[a,b]	AFM51700.1	GO:0005737	C:cytoplasm	0.7
170	Mbov_0333[a,b]	AFM51701.1	GO:0005737	C:cytoplasm	0.7
171	Mbov_0334[a,b]	AFM51702.1	GO:0005737	C:cytoplasm	0.7
172	Mbov_0336[a,b]	AFM51704.1	GO:0005737	C:cytoplasm	0.7
173	Mbov_0338[a]	AFM51706.1	GO:0005737	C:cytoplasm	0.7
174	Mbov_0339[b]	AFM51707.1	GO:0005615	C:extracellular space	0.91
175	Mbov_0341[b]	AFM51709.1	GO:0005615	C:extracellular space	0.91
176	Mbov_0350[a,b]	AFM51716.1	GO:0005615	C:extracellular space	0.97
177	Mbov_0353[a,b]	AFM51719.2	GO:0005737	C:cytoplasm	0.7
178	Mbov_0364[a,b]	AFM51726.1	GO:0005615	C:extracellular space	0.96
179	Mbov_0368[b]	AFM51730.1	GO:0005615	C:extracellular space	0.97
180	Mbov_0369[a,b]	AFM51731.1	GO:0005737	C:cytoplasm	0.7
181	Mbov_0370[a,b]	AFM51732.1	GO:0005737	C:cytoplasm	0.7
182	Mbov_0371[a,b]	AFM51733.1	GO:0005737	C:cytoplasm	0.7
183	Mbov_0374[b]	AFM51736.1	GO:0005615	C:extracellular space	0.97
184	Mbov_0375[a,b]	AFM51737.1	GO:0005737	C:cytoplasm	0.7
185	Mbov_0376[a]	AFM51738.1	GO:0005737	C:cytoplasm	0.7
186	Mbov_0393[a,b]	AFM51750.1	GO:0005615	C:extracellular space	0.96
187	Mbov_0400[a,b]	AFM51757.1	GO:0005737	C:cytoplasm	0.7
188	Mbov_0403[a,b]	AFM51760.1	GO:0005737	C:cytoplasm	0.7
189	Mbov_0404[a,b]	AFM51761.1	GO:0005737	C:cytoplasm	0.7
190	Mbov_0408[a,b]	AFM51765.1	GO:0005737	C:cytoplasm	0.7
191	Mbov_0411[a,b]	AFM51768.1	GO:0005737	C:cytoplasm	0.7
192	Mbov_0412[a,b]	AFM51769.1	GO:0005737	C:cytoplasm	0.7
193	Mbov_0418[a,b]	AFM51775.2	GO:0005886	C:plasma membrane	0.84
194	Mbov_0419[a,b]	AFM51776.1	GO:0005737	C:cytoplasm	0.7
195	Mbov_0421[a,b]	AFM51778.1	GO:0005737	C:cytoplasm	0.7
196	Mbov_0422[a,b]	AFM51779.2	GO:0005737	C:cytoplasm	0.7
197	Mbov_0425[a,b]	AFM51782.1	GO:0005737	C:cytoplasm	0.7
198	Mbov_0426[a,b]	AFM51783.1	GO:0005737	C:cytoplasm	0.7
199	Mbov_0427[a,b]	AFM51784.1	GO:0005737	C:cytoplasm	0.7
200	Mbov_0428[a,b]	AFM51785.1	GO:0005737	C:cytoplasm	0.7
201	Mbov_0429[a,b]	AFM51786.1	GO:0005737	C:cytoplasm	0.7

202	Mbov_0430[a,b]	AFM51787.1	GO:0005737	C:cytoplasm	0.7
203	Mbov_0432[a,b]	AFM51789.1	GO:0005737	C:cytoplasm	0.7
204	Mbov_0433[a,b]	AFM51790.1	GO:0005737	C:cytoplasm	0.7
205	Mbov_0438[a,b]	AFM51795.1	GO:0005737	C:cytoplasm	0.7
206	Mbov_0440[a,b]	AFM51797.1	GO:0005737	C:cytoplasm	0.7
207	Mbov_0446[a,b]	AFM51803.1	GO:0005737	C:cytoplasm	0.7
208	Mbov_0449[a,b]	AFM51806.2	GO:0005615	C:extracellular space	0.95
209	Mbov_0451[a,b]	AFM51808.1	GO:0005737	C:cytoplasm	0.7
210	Mbov_0452[a,b]	AFM51809.1	GO:0005737	C:cytoplasm	0.7
211	Mbov_0458[a,b]	AFM51815.1	GO:0005615	C:extracellular space	0.96
212	Mbov_0461[a,b]	AFM51818.1	GO:0005615	C:extracellular space	0.96
213	Mbov_0462[a,b]	AFM51819.1	GO:0005615	C:extracellular space	0.97
214	Mbov_0467[b]	AFM51823.1	GO:0005615	C:extracellular space	0.93
215	Mbov_0468[a,b]	AFM51824.1	GO:0005615	C:extracellular space	0.97
216	Mbov_0469[a,b]	AFM51825.1	GO:0005615	C:extracellular space	0.97
217	Mbov_0471[a,b]	AFM51827.1	GO:0005615	C:extracellular space	0.92
218	Mbov_0473[a,b]	AFM51829.1	GO:0005615	C:extracellular space	0.91
219	Mbov_0478[a,b]	AFM51834.1	GO:0005737	C:cytoplasm	0.7
220	Mbov_0479[a]	AFM51835.1	GO:0005737	C:cytoplasm	0.7
221	Mbov_0480[a,b]	AFM51836.1	GO:0005737	C:cytoplasm	0.7
222	Mbov_0481[a,b]	AFM51837.1	GO:0005737	C:cytoplasm	0.7
223	Mbov_0482[a,b]	AFM51838.1	GO:0005737	C:cytoplasm	0.7
224	Mbov_0485[a,b]	AFM51841.1	GO:0005886	C:plasma membrane	0.64
225	Mbov_0486[a]	AFM51842.1	GO:0005737	C:cytoplasm	0.7
226	Mbov_0487[a]	AFM51843.1	GO:0005737	C:cytoplasm	0.7
227	Mbov_0488[a,b]	AFM51844.1	GO:0005737	C:cytoplasm	0.7
228	Mbov_0492[a,b]	AFM51848.1	GO:0005886	C:plasma membrane	0.78
229	Mbov_0493[a,b]	AFM51849.1	GO:0005737	C:cytoplasm	0.7
230	Mbov_0494[a,b]	AFM51850.1	GO:0005737	C:cytoplasm	0.7
231	Mbov_0495[a,b]	AFM51851.1	GO:0005886	C:plasma membrane	0.76
232	Mbov_0499[a,b]	AFM51855.1	GO:0005737	C:cytoplasm	0.7
233	Mbov_0500[a,b]	AFM51856.1	GO:0005737	C:cytoplasm	0.7
234	Mbov_0502[a,b]	AFM51858.1	GO:0005737	C:cytoplasm	0.7
235	Mbov_0504[a,b]	AFM51860.1	GO:0005737	C:cytoplasm	0.7

236	Mbov_0505[a,b]	AFM51861.1	GO:0005615	C:extracellular space	0.78
237	Mbov_0508[a,b]	AFM51862.1	GO:0005737	C:cytoplasm	0.7
238	Mbov_0509[a,b]	AFM51863.1	GO:0005737	C:cytoplasm	0.7
239	Mbov_0511[a,b]	AFM51865.1	GO:0005737	C:cytoplasm	0.7
240	Mbov_0513[a,b]	AFM51867.1	GO:0005737	C:cytoplasm	0.7
241	Mbov_0515[a,b]	AFM51869.1	GO:0005615	C:extracellular space	0.97
242	Mbov_0516[a,b]	AFM51870.1	GO:0005615	C:extracellular space	0.96
243	Mbov_0517[a,b]	AFM51871.1	GO:0005615	C:extracellular space	0.95
244	Mbov_0518[b]	AFM51872.1	GO:0005615	C:extracellular space	0.96
245	Mbov_0519[a,b]	AFM51873.1	GO:0005615	C:extracellular space	0.79
246	Mbov_0520[a,b]	AFM51874.1	GO:0005737	C:cytoplasm	0.7
247	Mbov_0521[a]	AFM51875.1	GO:0005886	C:plasma membrane	0.7
248	Mbov_0522[a,b]	AFM51876.1	GO:0005737	C:cytoplasm	0.7
249	Mbov_0528[a,b]	AFM51882.1	GO:0005737	C:cytoplasm	0.7
250	Mbov_0529[a,b]	AFM51883.1	GO:0005737	C:cytoplasm	0.7
251	Mbov_0530[a]	AFM51884.1	GO:0005737	C:cytoplasm	0.7
252	Mbov_0531[a]	AFM51885.1	GO:0005737	C:cytoplasm	0.7
253	Mbov_0532[a,b]	AFM51886.1	GO:0005737	C:cytoplasm	0.7
254	Mbov_0534[a,b]	AFM51888.1	GO:0005886	C:plasma membrane	0.81
255	Mbov_0535[a,b]	AFM51889.1	GO:0005737	C:cytoplasm	0.7
256	Mbov_0536[a,b]	AFM51890.1	GO:0005615	C:extracellular space	0.87
257	Mbov_0537[a,b]	AFM51891.1	GO:0005615	C:extracellular space	0.93
258	Mbov_0539[a,b]	AFM51893.1	GO:0005737	C:cytoplasm	0.7
259	Mbov_0541[a,b]	AFM51895.1	GO:0005737	C:cytoplasm	0.7
260	Mbov_0542[a,b]	AFM51896.1	GO:0005737	C:cytoplasm	0.7
261	Mbov_0543[a,b]	AFM51897.1	GO:0005737	C:cytoplasm	0.7
262	Mbov_0548[a,b]	AFM51902.1	GO:0005737	C:cytoplasm	0.7
263	Mbov_0549[a,b]	AFM51903.1	GO:0005737	C:cytoplasm	0.7
264	Mbov_0551[b]	AFM51905.1	GO:0005737	C:cytoplasm	0.7
265	Mbov_0552[a,b]	AFM51906.1	GO:0005886	C:plasma membrane	0.46
266	Mbov_0553[a]	AFM51907.1	GO:0005886	C:plasma membrane	0.93
267	Mbov_0554[a]	AFM51908.1	GO:0005737	C:cytoplasm	0.7
268	Mbov_0555[a,b]	AFM51909.1	GO:0005737	C:cytoplasm	0.7
269	Mbov_0557[a,b]	AFM51911.1	GO:0005886	C:plasma membrane	0.81

270	Mbov_0558[a,b]	AFM51912.1	GO:0005737	C:cytoplasm	0.7
271	Mbov_0563[a,b]	AFM51917.2	GO:0005886	C:plasma membrane	0.6
272	Mbov_0565[a,b]	AFM51919.1	GO:0005737	C:cytoplasm	0.7
273	Mbov_0566[a,b]	AFM51920.1	GO:0005737	C:cytoplasm	0.7
274	Mbov_0567[a,b]	AFM51921.1	GO:0005737	C:cytoplasm	0.7
275	Mbov_0568[a,b]	AFM51922.1	GO:0005737	C:cytoplasm	0.7
276	Mbov_0570[a,b]	AFM51924.1	GO:0005615	C:extracellular space	0.9
277	Mbov_0571[a,b]	AFM51925.1	GO:0005886	C:plasma membrane	0.86
278	Mbov_0574[a,b]	AFM51928.1	GO:0005737	C:cytoplasm	0.7
279	Mbov_0576[a,b]	AFM51930.1	GO:0005737	C:cytoplasm	0.7
280	Mbov_0579[a,b]	AFM51933.1	GO:0005615	C:extracellular space	0.96
281	Mbov_0580[a,b]	AFM51934.1	GO:0005615	C:extracellular space	0.97
282	Mbov_0581[a,b]	AFM51935.1	GO:0005737	C:cytoplasm	0.7
283	Mbov_0585[a,b]	AFM51939.1	GO:0005615	C:extracellular space	0.96
284	Mbov_0586[a,b]	AFM51940.1	GO:0005737	C:cytoplasm	0.7
285	Mbov_0587[a,b]	AFM51941.1	GO:0005737	C:cytoplasm	0.7
286	Mbov_0588[a,b]	AFM51942.1	GO:0005737	C:cytoplasm	0.7
287	Mbov_0589[a,b]	AFM51943.1	GO:0005737	C:cytoplasm	0.7
288	Mbov_0590[a,b]	AFM51944.1	GO:0005737	C:cytoplasm	0.7
289	Mbov_0591[a,b]	AFM51945.1	GO:0005737	C:cytoplasm	0.7
290	Mbov_0594[a,b]	AFM51948.1	GO:0005737	C:cytoplasm	0.7
291	Mbov_0596[a,b]	AFM51950.1	GO:0005737	C:cytoplasm	0.7
292	Mbov_0597[a,b]	AFM51951.1	GO:0005737	C:cytoplasm	0.7
293	Mbov_0598[a,b]	AFM51952.1	GO:0005737	C:cytoplasm	0.7
294	Mbov_0599[a,b]	AFM51953.1	GO:0005737	C:cytoplasm	0.7
295	Mbov_0600[a,b]	AFM51954.1	GO:0005737	C:cytoplasm	0.7
296	Mbov_0601[a,b]	AFM51955.1	GO:0005737	C:cytoplasm	0.7
297	Mbov_0602[a,b]	AFM51956.1	GO:0005737	C:cytoplasm	0.7
298	Mbov_0603[a,b]	AFM51957.1	GO:0005737	C:cytoplasm	0.7
299	Mbov_0606[a,b]	AFM51959.1	GO:0005737	C:cytoplasm	0.7
300	Mbov_0611[a]	AFM51962.1	GO:0005737	C:cytoplasm	0.7
301	Mbov_0612[a,b]	AFM51963.1	GO:0005737	C:cytoplasm	0.7
302	Mbov_0613[a,b]	AFM51964.1	GO:0005737	C:cytoplasm	0.7
303	Mbov_0614[a,b]	AFM51965.1	GO:0005737	C:cytoplasm	0.7

304	Mbov_0617[a,b]	AFM51968.1	GO:0005737	C:cytoplasm	0.7
305	Mbov_0618[a,b]	AFM51969.1	GO:0005737	C:cytoplasm	0.7
306	Mbov_0619[a,b]	AFM51970.1	GO:0005737	C:cytoplasm	0.7
307	Mbov_0620[a,b]	AFM51971.1	GO:0005737	C:cytoplasm	0.7
308	Mbov_0621[a,b]	AFM51972.1	GO:0005737	C:cytoplasm	0.7
309	Mbov_0622[a]	AFM51973.1	GO:0005737	C:cytoplasm	0.7
310	Mbov_0623[a,b]	AFM51974.1	GO:0005737	C:cytoplasm	0.7
311	Mbov_0624[a,b]	AFM51975.1	GO:0005737	C:cytoplasm	0.7
312	Mbov_0625[a,b]	AFM51976.1	GO:0005737	C:cytoplasm	0.7
313	Mbov_0626[a,b]	AFM51977.1	GO:0005737	C:cytoplasm	0.7
314	Mbov_0627[a,b]	AFM51978.1	GO:0005737	C:cytoplasm	0.7
315	Mbov_0628[a]	AFM51979.1	GO:0005737	C:cytoplasm	0.7
316	Mbov_0629[a,b]	AFM51980.1	GO:0005737	C:cytoplasm	0.7
317	Mbov_0630[a,b]	AFM51981.1	GO:0005737	C:cytoplasm	0.7
318	Mbov_0631[b]	AFM51982.1	GO:0005737	C:cytoplasm	0.7
319	Mbov_0634[a,b]	AFM51985.1	GO:0005737	C:cytoplasm	0.7
320	Mbov_0635[a,b]	AFM51986.1	GO:0005737	C:cytoplasm	0.7
321	Mbov_0636[a]	AFM51987.1	GO:0005737	C:cytoplasm	0.7
322	Mbov_0637[a,b]	AFM51988.1	GO:0005737	C:cytoplasm	0.7
323	Mbov_0638[a,b]	AFM51989.1	GO:0005737	C:cytoplasm	0.7
324	Mbov_0641[a,b]	AFM51992.1	GO:0005737	C:cytoplasm	0.7
325	Mbov_0642[a,b]	AFM51993.1	GO:0005737	C:cytoplasm	0.7
326	Mbov_0643[a]	AFM51994.1	GO:0005737	C:cytoplasm	0.7
327	Mbov_0644[a,b]	AFM51995.1	GO:0005737	C:cytoplasm	0.7
328	Mbov_0646[a,b]	AFM51997.1	GO:0005737	C:cytoplasm	0.7
329	Mbov_0647[a]	AFM51998.1	GO:0005737	C:cytoplasm	0.7
330	Mbov_0650[a,b]	AFM52001.1	GO:0005737	C:cytoplasm	0.7
331	Mbov_0651[a,b]	AFM52002.1	GO:0005737	C:cytoplasm	0.7
332	Mbov_0652[a,b]	AFM52003.1	GO:0005737	C:cytoplasm	0.7
333	Mbov_0654[a,b]	AFM52005.1	GO:0005615	C:extracellular space	0.8
334	Mbov_0656[a,b]	AFM52007.1	GO:0005615	C:extracellular space	0.8
335	Mbov_0658[a,b]	AFM52009.1	GO:0005615	C:extracellular space	0.92
336	Mbov_0660[a,b]	AFM52011.1	GO:0005737	C:cytoplasm	0.7
337	Mbov_0661[a,b]	AFM52012.1	GO:0005737	C:cytoplasm	0.7

338	Mbov_0663[a,b]	AFM52014.1	GO:0005737	C:cytoplasm	0.7
339	Mbov_0665[a]	AFM52016.1	GO:0005737	C:cytoplasm	0.7
340	Mbov_0666[a,b]	AFM52017.1	GO:0005737	C:cytoplasm	0.7
341	Mbov_0667[a,b]	AFM52018.1	GO:0005737	C:cytoplasm	0.7
342	Mbov_0668[a,b]	AFM52019.1	GO:0005737	C:cytoplasm	0.7
343	Mbov_0669[a,b]	AFM52020.1	GO:0005737	C:cytoplasm	0.7
344	Mbov_0673[a,b]	AFM52023.1	GO:0005737	C:cytoplasm	0.7
345	Mbov_0674[a,b]	AFM52024.1	GO:0005615	C:extracellular space	0.87
346	Mbov_0675[a,b]	AFM52025.1	GO:0005615	C:extracellular space	0.97
347	Mbov_0676[a,b]	AFM52026.1	GO:0005737	C:cytoplasm	0.7
348	Mbov_0677 [a,b]	AFM52027.1	GO:0005737	C:cytoplasm	0.7
349	Mbov_0678[a,b]	AFM52028.1	GO:0005737	C:cytoplasm	0.7
350	Mbov_0679[a,b]	AFM52029.1	GO:0005737	C:cytoplasm	0.7
351	Mbov_0680[a,b]	AFM52030.1	GO:0005737	C:cytoplasm	0.7
352	Mbov_0687[a,b]	AFM52036.1	GO:0005886	C:plasma membrane	0.84
353	Mbov_0691[a,b]	AFM52040.1	GO:0005737	C:cytoplasm	0.7
354	Mbov_0693[a,b]	AFM52042.1	GO:0005615	C:extracellular space	0.95
355	Mbov_0694[a,b]	AFM52043.1	GO:0005737	C:cytoplasm	0.7
356	Mbov_0695[a,b]	AFM52044.1	GO:0005737	C:cytoplasm	0.7
357	Mbov_0696[a,b]	AFM52045.1	GO:0005615	C:extracellular space	0.96
358	Mbov_0697[a,b]	AFM52046.1	GO:0005737	C:cytoplasm	0.7
359	Mbov_0698[a,b]	AFM52047.1	GO:0005737	C:cytoplasm	0.7
360	Mbov_0700[a,b]	AFM52049.1	GO:0005737	C:cytoplasm	0.7
361	Mbov_0701[a,b]	AFM52050.1	GO:0005737	C:cytoplasm	0.7
362	Mbov_0702[a,b]	AFM52051.1	GO:0005737	C:cytoplasm	0.7
363	Mbov_0703[a,b]	AFM52052.1	GO:0005737	C:cytoplasm	0.7
364	Mbov_0710[a,b]	AFM52059.1	GO:0005737	C:cytoplasm	0.7
365	Mbov_0711[a,b]	AFM52060.1	GO:0005737	C:cytoplasm	0.7
366	Mbov_0712[a,b]	AFM52061.1	GO:0005737	C:cytoplasm	0.7
367	Mbov_0713[a,b]	AFM52062.1	GO:0005737	C:cytoplasm	0.7
368	Mbov_0715[a,b]	AFM52064.1	GO:0005737	C:cytoplasm	0.7
369	Mbov_0718[a]	AFM52067.1	GO:0005737	C:cytoplasm	0.7
370	Mbov_0719[a]	AFM52068.1	GO:0005737	C:cytoplasm	0.7
371	Mbov_0720[a,b]	AFM52069.1	GO:0005737	C:cytoplasm	0.7

372	Mbov_0721[a]	AFM52070.1	GO:0005737	C:cytoplasm	0.7
373	Mbov_0722[a]	AFM52071.2	GO:0005737	C:cytoplasm	0.7
374	Mbov_0724[a]	AFM52073.1	GO:0005737	C:cytoplasm	0.7
375	Mbov_0725[a,b]	AFM52074.1	GO:0005737	C:cytoplasm	0.7
376	Mbov_0732[a]	AFM52081.1	GO:0005737	C:cytoplasm	0.7
377	Mbov_0734[a]	AFM52083.1	GO:0005737	C:cytoplasm	0.7
378	Mbov_0739[a,b]	AFM52087.1	GO:0005615	C:extracellular space	0.96
379	Mbov_0743[a,b]	AFM52091.1	GO:0005615	C:extracellular space	0.82
380	Mbov_0745[a,b]	AFM52093.1	GO:0005737	C:cytoplasm	0.7
381	Mbov_0746[a]	AFM52094.1	GO:0005737	C:cytoplasm	0.7
382	Mbov_0747[a,b]	AFM52095.1	GO:0005737	C:cytoplasm	0.7
383	Mbov_0749[a]	AFM52097.1	GO:0005737	C:cytoplasm	0.7
384	Mbov_0750[a]	AFM52098.1	GO:0005737	C:cytoplasm	0.7
385	Mbov_0755[a,b]	AFM52103.1	GO:0005737	C:cytoplasm	0.7
386	Mbov_0758[a]	AFM52106.1	GO:0005737	C:cytoplasm	0.7
387	Mbov_0760[a,b]	AFM52108.1	GO:0005886	C:plasma membrane	0.87
388	Mbov_0761[a,b]	AFM52109.1	GO:0005737	C:cytoplasm	0.7
389	Mbov_0765[a,b]	AFM52113.1	GO:0005737	C:cytoplasm	0.7
390	Mbov_0768[a,b]	AFM52116.1	GO:0005615	C:extracellular space	0.97
391	Mbov_0771[a,b]	AFM52119.1	GO:0005737	C:cytoplasm	0.7
392	Mbov_0772[a,b]	AFM52120.1	GO:0005737	C:cytoplasm	0.7
393	Mbov_0773[a,b]	AFM52121.1	GO:0005737	C:cytoplasm	0.7
394	Mbov_0774[a,b]	AFM52122.1	GO:0005737	C:cytoplasm	0.7
395	Mbov_0776[a,b]	AFM52124.1	GO:0005737	C:cytoplasm	0.7
396	Mbov_0777[a,b]	AFM52125.1	GO:0005737	C:cytoplasm	0.7
397	Mbov_0778[a,b]	AFM52126.1	GO:0005886	C:plasma membrane	0.9
398	Mbov_0779[a]	AFM52127.1	GO:0005886	C:plasma membrane	0.85
399	Mbov_0783[a,b]	AFM52131.1	GO:0005737	C:cytoplasm	0.7
400	Mbov_0784[a,b]	AFM52132.1	GO:0005737	C:cytoplasm	0.7
401	Mbov_0786[a,b]	AFM52134.1	GO:0005737	C:cytoplasm	0.7
402	Mbov_0787[a,b]	AFM52135.1	GO:0005737	C:cytoplasm	0.7
403	Mbov_0788[a,b]	AFM52136.1	GO:0005737	C:cytoplasm	0.7
404	Mbov_0789[a,b]	AFM52137.1	GO:0005737	C:cytoplasm	0.7
405	Mbov_0793[a,b]	AFM52141.1	GO:0005615	C:extracellular space	0.9

406	Mbov_0794[a,b]	AFM52142.1	GO:0005615	C:extracellular space	0.9
407	Mbov_0795[a]	AFM52143.1	GO:0005615	C:extracellular space	0.93
408	Mbov_0796[a,b]	AFM52144.1	GO:0005615	C:extracellular space	0.95
409	Mbov_0797[a,b]	AFM52145.1	GO:0005615	C:extracellular space	0.96
410	Mbov_0798[a,b]	AFM52146.1	GO:0005615	C:extracellular space	0.96
411	Mbov_0802[a,b]	AFM52150.1	GO:0005737	C:cytoplasm	0.7
412	Mbov_0804[a,b]	AFM52152.2	GO:0005737	C:cytoplasm	0.7
413	Mbov_0806[a,b]	AFM52154.1	GO:0005737	C:cytoplasm	0.7
414	Mbov_0807[a,b]	AFM52155.1	GO:0005737	C:cytoplasm	0.7
415	Mbov_0808[a,b]	AFM52156.1	GO:0005737	C:cytoplasm	0.7
316	Mbov_0810[a,b]	AFM52158.1	GO:0005737	C:cytoplasm	0.7
417	Mbov_0811[a,b]	AFM52159.1	GO:0005737	C:cytoplasm	0.7
418	Mbov_0813[a,b]	AFM52161.1	GO:0005737	C:cytoplasm	0.7
419	Mbov_0814[a,b]	AFM52162.1	GO:0005737	C:cytoplasm	0.7
420	Mbov_0815[a,b]	AFM52163.1	GO:0005737	C:cytoplasm	0.7
421	Mbov_0817[a,b]	AFM52165.1	GO:0005737	C:cytoplasm	0.7
422	Mbov_0818[a,b]	AFM52166.2	GO:0005737	C:cytoplasm	0.7
423	Mbov_0819[a,b]	AFM52167.1	GO:0005737	C:cytoplasm	0.7
424	Mbov_0820[a,b]	AFM52168.1	GO:0005737	C:cytoplasm	0.7
425	Mbov_0821[a,b]	AFM52169.1	GO:0005737	C:cytoplasm	0.7
426	Mbov_0822[a,b]	AFM52170.1	GO:0005737	C:cytoplasm	0.7
427	Mbov_0823[a,b]	AFM52171.1	GO:0005737	C:cytoplasm	0.7
428	Mbov_0824[a,b]	AFM52172.2	GO:0005737	C:cytoplasm	0.7
429	Mbov_0825[a,b]	AFM52173.1	GO:0005737	C:cytoplasm	0.7
430	Mbov_0831[a,b]	AFM52176.1	GO:0005737	C:cytoplasm	0.7
431	Mbov_0832[a,b]	AFM52177.1	GO:0005737	C:cytoplasm	0.7
432	Mbov_0833[a]	AFM52178.1	GO:0005737	C:cytoplasm	0.7
433	Mbov_0838[a,b]	AFM52183.1	GO:0005737	C:cytoplasm	0.7
434	Mbov_0839[a]	AFM52184.1	GO:0005737	C:cytoplasm	0.7
435	Mbov_0843[a]	AFM52188.1	GO:0005737	C:cytoplasm	0.7
436	Mbov_0845[a,b]	AFM52189.1	GO:0005886	C:plasma membrane	0.89
437	Mbov_0847[a,b]	AFM52191.1	GO:0005737	C:cytoplasm	0.7
438	Mbov_0848[a]	AFM52192.1	GO:0005737	C:cytoplasm	0.7

a: related to *M. bovis* HB0801-P1, b: related to P150 and a,b: related to both (*M. bovis* HB0801-P1, and P150).

Table S2 Subcellular localization of *M. bovis* proteins prediction by using PSORTb 3.0 server.

No	Proteins ID	Accession No	Protein Name	Final Prediction	Localization Score
1	Mbov_0001[a]	AFM51379.1	replication initiation protein	Cytoplasmic	9.97
2	Mbov_0002[a,b]	AFM51380.1	DNA polymerase III subunit beta	Unknown	
3	Mbov_0003[a]	AFM51381.1	hypothetical protein Mbov_0003	Unknown	
4	Mbov_0004[a,b]	AFM51382.1	transposase ISMbov7	Unknown	
5	Mbov_0006[a,b]	AFM51384.1	esterase/lipase	Unknown	
6	Mbov_0007[a,b]	AFM51385.1	lipase	Unknown	
7	Mbov_0008[a,b]	AFM51386.1	NADH dependent flavin oxidoreductase	Cytoplasmic	7.50
8	Mbov_0009[a,b]	AFM51387.1	lipoate-protein ligase A	Cytoplasmic	9.97
9	Mbov_0010[a,b]	AFM51388.1	lipoate-protein ligase A	Cytoplasmic	9.97
10	Mbov_0011[a,b]	AFM51389.1	trimethylamine dehydrogenase	Unknown	
11	Mbov_0012[a,b]	AFM51390.1	glycine cleavage system H protein	Unknown	
12	Mbov_0016[a,b]	AFM51394.1	P48-like surface lipoprotein	Unknown	
13	Mbov_0018[a,b]	AFM51396.1	simple sugar ABC transporter ATP-binding protein.P59-like protein	CytoplasmicMembrane	9.99
14	Mbov_0022[a,b]	AFM51400.1	deoxyguanosine kinase	Cytoplasmic	7.50
15	Mbov_0023[a,b]	AFM51401.1	deoxyguanosine kinase	Unknown	
16	Mbov_0025[a,b]	AFM51403.1	hypothetical protein Mbov_0025	Cytoplasmic	7.50
17	Mbov_0026[a,b]	AFM51404.1	ribokinase family sugar kinase	Unknown	
18	Mbov_0027[a]	AFM51405.1	putative Hydrolase of the HAD superfamily	Cytoplasmic	7.50
19	Mbov_0037[a,b]	AFM51415.2	oligopeptide ABC transporter substrate-binding protein	Unknown	
20	Mbov_0038[a,b]	AFM51416.1	putative transmembrane protein	Unknown	
21	Mbov_0044[a]	AFM51419.1	RNA methyltransferase	Unknown	
22	Mbov_0048[a,b]	AFM51423.1	transcriptional antiterminator	Cytoplasmic	7.50
23	Mbov_0049[a,b]	AFM51424.1	putative lipoprotein	Cytoplasmic	7.50
24	Mbov_0051[a,b]	AFM51426.1	uridylate kinase	Cytoplasmic	7.50
25	Mbov_0052[a,b]	AFM51427.1	ribosome recycling factor	Cytoplasmic	9.97
26	Mbov_0056[a,b]	AFM51431.1	glycerol-3-phosphate dehydrogenase	Unknown	
27	Mbov_0058[a]	AFM51433.1	TatD DNase family protein	Cytoplasmic	9.97
28	Mbov_0059[a,b]	AFM51434.1	tRNA modification GTPase	Cytoplasmic	9.97
29	Mbov_0062[a,b]	AFM51437.1	glyceraldehyde 3-phosphate dehydrogenase	Cytoplasmic	9.97

30	Mbov_0063[a,b]	AFM51438.1	seryl-tRNA synthetase	Cytoplasmic	10.00
31	Mbov_0068[a,b]	AFM51443.1	lipoate-protein ligase A	Cytoplasmic	9.97
32	Mbov_0069[a,b]	AFM51444.1	putative hydrolase/ acyltransferase	Unknown	
33	Mbov_0071[a,b]	AFM51446.1	aspartate ammonia ligase	Cytoplasmic	10.00
34	Mbov_0072[a,b]	AFM51447.1	DNA polymerase III subunit alpha	Cytoplasmic	9.97
35	Mbov_0074[a,b]	AFM51449.1	phenylalanyl-tRNA synthetase alpha chain	Cytoplasmic	10.00
36	Mbov_0075[a]	AFM51450.1	uracil-DNA glycosylase	Cytoplasmic	9.67
37	Mbov_0076[a,b]	AFM51451.1	phenylalanyl-tRNA synthetase beta chain	Cytoplasmic	9.97
38	Mbov_0077[a,b]	AFM51452.1	serine hydroxymethyltransferase	Cytoplasmic	9.97
39	Mbov_0078[a,b]	AFM51453.1	proline iminopeptidase	Cytoplasmic	9.97
40	Mbov_0079[a]	AFM51454.1	cysteine desulfurase	Cytoplasmic	9.67
41	Mbov_0080[a,b]	AFM51455.1	nitrogen fixation protein	Cytoplasmic	7.50
42	Mbov_0082[a,b]	AFM51457.1	nicotinate-nucleotide adenylyltransferase	Cytoplasmic	7.50
43	Mbov_0083[a,b]	AFM51458.1	ribosomal large subunit pseudouridine synthase B	Cytoplasmic	9.97
44	Mbov_0085[a,b]	AFM51460.1	Hypothetical protein Mbov_0085	Cytoplasmic	7.50
45	Mbov_0086[a,b]	AFM51461.1	fructose-bisphosphate aldolase	Unknown	
46	Mbov_0088[a,b]	AFM51463.1	50S ribosomal protein	Cytoplasmic	9.97
47	Mbov_0089[a,b]	AFM51464.1	50S ribosomal protein	Cytoplasmic	9.67
48	Mbov_0093[a]	AFM51468.1	RNA methyltransferase	Unknown	
49	Mbov_0094[a,b]	AFM51469.1	RNA methyltransferase	Cytoplasmic	7.50
50	Mbov_0095[a]	AFM51470.1	Hypothetical protein Mbov_0095	Unknown	
51	Mbov_0096[a]	AFM51471.1	putative GTPase	CytoplasmicMembrane	9.55
52	Mbov_0098[a,b]	AFM51473.1	HPr kinase/phosphorylase	Cytoplasmic	7.50
53	Mbov_0100[a,b]	AFM51475.1	thioredoxin reductase	Cytoplasmic	9.97
54	Mbov_0102[a,b]	AFM51477.1	pyruvate dehydrogenase E1 component subunit alpha	Cytoplasmic	9.97
55	Mbov_0103[a,b]	AFM51478.1	pyruvate dehydrogenase E1 component subunit beta	Cytoplasmic	7.50
56	Mbov_0104[a,b]	AFM51479.1	Hypothetical protein Mbov_0104	Cytoplasmic	7.50
57	Mbov_0105[a,b]	AFM51480.1	dihydrolipoamide succinyltransferase	Cytoplasmic	9.97
58	Mbov_0106[a,b]	AFM51481.1	dihydrolipoamide dehydrogenase	Cytoplasmic	9.97
59	Mbov_0107[a,b]	AFM51482.1	transposase ISMbov1	Unknown	
60	Mbov_0109[a,b]	AFM51484.1	monophosphate biosynthesis	Cytoplasmic	7.50
61	Mbov_0111[a,b]	AFM51486.1	putative lipoprotein	Unknown	
62	Mbov_0114[a,b]	AFM51489.1	oligopeptide ABC transporter ATP-binding protein	CytoplasmicMembrane	8.78
63	Mbov_0115[a,b]	AFM51490.1	oligopeptide ABC transporter ATP-binding protein	CytoplasmicMembrane	9.99

64	Mbov_0120[a,b]	AFM51495.2	ribose 5-phosphate isomerase B	Cytoplasmic	7.50
65	Mbov_0123[a,b]	AFM51498.2	hypothetical protein (metal-dependent hydrolase)	Cytoplasmic	7.50
66	Mbov_0124[a,b]	AFM51499.2	cytidine deaminase	Cytoplasmic	7.50
67	Mbov_0125[a,b]	AFM51500.1	GTP-binding protein	CytoplasmicMembrane	8.78
68	Mbov_0126[a,b]	AFM51501.1	XAA-Pro aminopeptidase	Cytoplasmic	9.97
69	Mbov_0127[a,b]	AFM51502.1	prolyl-tRNA synthetase	Cytoplasmic	10.00
70	Mbov_0131[a,b]	AFM51506.1	phosphoketolase	Unknown	
71	Mbov_0133[a,b]	AFM51508.1	oligoendopeptidase	Cytoplasmic	9.97
72	Mbov_0139[a]	AFM51514.1	50S ribosomal protein	Cytoplasmic	9.67
73	Mbov_0141[a]	AFM51516.1	thiamine biosynthesis protein	Cytoplasmic	9.95
74	Mbov_0145[a]	AFM51518.1	putative lipoprotein	Unknown	
75	Mbov_0148[a,b]	AFM51521.1	valyl-tRNA synthetase	Cytoplasmic	9.67
76	Mbov_0149[a,b]	AFM51522.1	methylenetetrahydrofolate dehydrogenase (NADP)	Unknown	
77	Mbov_0150[a,b]	AFM51523.1	phosphate acetyltransferase	Cytoplasmic	7.50
78	Mbov_0151[a,b]	AFM51524.1	acetate kinase	Cytoplasmic	9.97
79	Mbov_0152[a,b]	AFM51525.1	pantetheine-phosphate adenylyltransferase	Cytoplasmic	9.67
80	Mbov_0153[a,b]	AFM51526.1	GTP-binding protein	CytoplasmicMembrane	9.51
81	Mbov_0154[a,b]	AFM51527.1	putative transmembrane protein	Unknown	
82	Mbov_0155[a,b]	AFM51528.1	pyruvate kinase	Cytoplasmic	7.50
83	Mbov_0156[a,b]	AFM51529.1	putative lipoprotein	Unknown	
84	Mbov_0157[a,b]	AFM51530.1	molecular chaperone DnaK	Cytoplasmic	9.97
85	Mbov_0158[a]	AFM51531.1	tRNA (uracil-5-)-methyltransferase Gid	Cytoplasmic	9.67
86	Mbov_0160[a,b]	AFM51533.1	D-lactate dehydrogenase	Cytoplasmic	9.97
87	Mbov_0165[a,b]	AFM51536.1	hypothetical protein Mbov_0165	Cytoplasmic	7.50
88	Mbov_0168[a,b]	AFM51537.1	trigger factor	Cytoplasmic	7.50
89	Mbov_0174[a,b]	AFM51543.1	P48-like surface lipoprotein	Unknown	
90	Mbov_0175[a,b]	AFM51544.1	DNA polymerase I	Unknown	
91	Mbov_0176[a,b]	AFM51545.1	DNA polymerase III subunit alpha	Cytoplasmic	9.97
92	Mbov_0190[a,b]	AFM51559.2	Hypothetical protein Mbov_0190	Unknown	
93	Mbov_0191[a,b]	AFM51560.1	glucose inhibited division protein	Cytoplasmic	7.50
94	Mbov_0192[a,b]	AFM51561.1	hypothetical protein Mbov_0192	Unknown	
95	Mbov_0196[a,b]	AFM51565.1	formylmethionyl-tRNA deformylase	Cytoplasmic	7.50
96	Mbov_0197[a,b]	AFM51566.1	putative transmembrane protein	CytoplasmicMembrane	10.00
97	Mbov_0198[a,b]	AFM51567.1	topoisomerase IV subunit B	Cytoplasmic	9.67

98	Mbov_0199[a]	AFM51568.1	topoisomerase IV subunit A	Cytoplasmic	9.67
99	Mbov_0200[a,b]	AFM51569.1	putative hydrolases of the HAD superfamily	Cytoplasmic	7.50
100	Mbov_0202[a,b]	AFM51571.1	DNA-methyltransferase	Cytoplasmic	7.50
101	Mbov_0203[a,b]	AFM51572.1	Hypothetical protein Mbov_0203	Unknown	
102	Mbov_0205[a,b]	AFM51574.1	methyltransferase GidB (glucose inhibited division protein)	Cytoplasmic	7.50
103	Mbov_0206[a,b]	AFM51575.1	ribose-phosphate pyrophosphokinase	Cytoplasmic	9.97
104	Mbov_0207[a]	AFM51576.1	Hypothetical protein Mbov_0207	Unknown	
105	Mbov_0210[a]	AFM51579.1	Hypothetical protein Mbov_0210	Unknown	
106	Mbov_0211[b]	AFM51580.1	Endonuclease I	Extracellular	9.73
107	Mbov_0212[a,b]	AFM51581.1	transketolase	Unknown	
108	Mbov_0213[a,b]	AFM51582.1	inorganic pyrophosphatase	Cytoplasmic	9.97
109	Mbov_0214[a,b]	AFM51583.1	30S ribosomal protein	Cytoplasmic	7.50
110	Mbov_0216[a,b]	AFM51585.1	putative transmembrane protein	Unknown	
111	Mbov_0217[a,b]	AFM51586.1	putative lipoprotein	Unknown	
112	Mbov_0218[a,b]	AFM51587.1	HIT-like protein	Cytoplasmic	7.50
113	Mbov_0220[a,b]	AFM51589.1	50S ribosomal protein	Cytoplasmic	9.67
114	Mbov_0222[a,b]	AFM51591.1	Thiol peroxidase	Cytoplasmic	7.50
115	Mbov_0223[a]	AFM51592.2	hypothetical protein Mbov_0223	Cytoplasmic	7.50
116	Mbov_0224[a,b]	AFM51593.1	signal recognition particle receptor	CytoplasmicMembrane	9.51
117	Mbov_0225[a,b]	AFM51594.1	Hypothetical protein Mbov_0225	Cytoplasmic	7.50
118	Mbov_0226[a,b]	AFM51595.1	methionyl-tRNA synthetase	Cytoplasmic	9.67
119	Mbov_0227[a,b]	AFM51596.1	putative O-methyltransferase	Cytoplasmic	7.50
120	Mbov_0228[a,b]	AFM51597.1	ribonuclease R	Cytoplasmic	9.97
121	Mbov_0231[a,b]	AFM51600.1	guanylate kinase	Cytoplasmic	9.97
122	Mbov_0232[a]	AFM51601.1	protein phosphatase	Unknown	
123	Mbov_0235[a,b]	AFM51604.1	ribulose-phosphate 3-epimerase	Cytoplasmic	7.50
124	Mbov_0237[a,b]	AFM51606.1	transposase ISMbov5	Cytoplasmic	7.50
125	Mbov_0239[a]	AFM51608.1	transposase ISMbov4	Cytoplasmic	7.50
126	Mbov_0242[a,b]	AFM51611.1	Hypothetical protein Mbov_0242	Unknown	
127	Mbov_0243[a,b]	AFM51612.1	type I restriction enzyme R subunit	Cytoplasmic	7.50
128	Mbov_0244[a,b]	AFM51613.1	type I restriction enzyme M protein	CytoplasmicMembrane	9.55
129	Mbov_0247[a,b]	AFM51616.1	type I restriction enzyme S subunit	Unknown	
130	Mbov_0255[a,b]	AFM51624.1	CTP synthetase	Unknown	
131	Mbov_0256[a,b]	AFM51625.1	isoleucyl-tRNA synthetase	Cytoplasmic	9.97

132	Mbov_0266[a,b]	AFM51635.1	tRNA pseudouridine synthase B	Cytoplasmic	9.67
133	Mbov_0267[a,b]	AFM51636.1	hypothetical protein Mbov_0267	Cytoplasmic	7.50
134	Mbov_0268[a,b]	AFM51637.1	riboflavin kinase / FMN adenylyltransferase	Cytoplasmic	7.50
135	Mbov_0269[a]	AFM51638.1	30S ribosomal protein	Cytoplasmic	9.97
136	Mbov_0270[a,b]	AFM51639.1	lipase	Cytoplasmic	7.50
137	Mbov_0274 [a]	AFM51642.1	putative lipoprotein	Unknown	
138	Mbov_0277[a,b]	AFM51645.1	50S ribosomal protein	Unknown	
139	Mbov_0278[a,b]	AFM51646.1	replicative DNA helicase	Unknown	
140	Mbov_0283[b]	AFM51651.1	putative lipoprotein	Unknown	
141	Mbov_0286[a,b]	AFM51654.1	NADH oxidase (NOXASE)	Cytoplasmic	9.97
142	Mbov_0290[b]	AFM51658.1	putative lipoprotein	Cytoplasmic	7.50
143	Mbov_0294[a,b]	AFM51662.1	30S ribosomal protein	Cytoplasmic	9.67
144	Mbov_0295[a,b]	AFM51663.1	elongation factor	Cytoplasmic	9.97
145	Mbov_0296[b]	AFM51664.1	putative lipoprotein	Unknown	
146	Mbov_0297[a,b]	AFM51665.2	hypothetical protein Mbov_0297	Unknown	
147	Mbov_0299[a,b]	AFM51667.1	NADH oxidase	Cytoplasmic	9.67
148	Mbov_0301[a,b]	AFM51669.1	hypothetical protein Mbov_0301	Unknown	
149	Mbov_0302[a,b]	AFM51670.1	RNA polymerase primary sigma factor	Cytoplasmic	9.97
150	Mbov_0303[a,b]	AFM51671.1	DNA primase	Cytoplasmic	7.50
151	Mbov_0304[a,b]	AFM51672.1	glycyl-tRNA synthetase	Cytoplasmic	10.00
152	Mbov_0305[a,b]	AFM51673.1	putative transmembrane protein	Cytoplasmic	7.50
153	Mbov_0309[a,b]	AFM51677.1	hypothetical protein Mbov_0309	Unknown	
154	Mbov_0310[a,b]	AFM51678.1	preprotein translocase subunit SecA	Cytoplasmic	9.97
155	Mbov_0312[a,b]	AFM51680.1	alcohol dehydrogenase	Cytoplasmic	9.67
156	Mbov_0313[a,b]	AFM51681.1	DNA topoisomerase I	Cytoplasmic	9.67
157	Mbov_0314[a,b]	AFM51682.1	30S ribosomal protein	Cytoplasmic	7.50
158	Mbov_0315[a,b]	AFM51683.1	putative single-strand DNA-binding protein	Unknown	
159	Mbov_0316[a]	AFM51684.1	30S ribosomal protein	Cytoplasmic	9.97
160	Mbov_0319[a,b]	AFM51687.1	utative translation factor	Unknown	
161	Mbov_0321[a,b]	AFM51689.1	histidyl-tRNA synthetase	Cytoplasmic	10.00
162	Mbov_0322[a,b]	AFM51690.1	aspartyl-tRNA synthetase	Cytoplasmic	10.00
163	Mbov_0325[a,b]	AFM51693.1	glycerol kinase	Cytoplasmic	9.97
164	Mbov_0326[b]	AFM51694.1	putative secreted acid phosphatase	Unknown	
165	Mbov_0327[a,b]	AFM51695.1	exopolyphosphatase-related protein	Unknown	

166	Mbov_0328[a,b]	AFM51696.1	exopolyphosphatase-related protein	Cytoplasmic	7.50
167	Mbov_0330[a,b]	AFM51698.1	Hypothetical protein Mbov_0330	Unknown	
168	Mbov_0331[a,b]	AFM51699.1	putative kinase related to dihydroxyacetone kinase	Cytoplasmic	7.50
169	Mbov_0332[a,b]	AFM51700.1	glycerol-3-phosphate acyltransferase plsX	Unknown	
170	Mbov_0333[a,b]	AFM51701.1	ribonuclease III	Cytoplasmic	9.67
171	Mbov_0334[a,b]	AFM51702.1	chromosome segregation protein	Cytoplasmic	9.97
172	Mbov_0336[a,b]	AFM51704.1	putative hydrolase	Unknown	
173	Mbov_0338[a]	AFM51706.1	alcohol dehydrogenase	Cytoplasmic	9.97
174	Mbov_0339[b]	AFM51707.1	variable surface lipoprotein VspY1	Unknown	
175	Mbov_0341[b]	AFM51709.1	putative transmembrane protein	Extracellular	9.13
176	Mbov_0350[a,b]	AFM51716.1	putative lipoprotein	Unknown	
177	Mbov_0353[a,b]	AFM51719.2	alcohol dehydrogenase	Cytoplasmic	9.97
178	Mbov_0364[a,b]	AFM51726.1	putative membrane lipoprotein	Unknown	
179	Mbov_0368[b]	AFM51730.1	putative transmembrane protein	Unknown	
180	Mbov_0369[a,b]	AFM51731.1	purine-nucleoside phosphorylase	Cytoplasmic	7.50
181	Mbov_0370[a,b]	AFM51732.1	tRNA-methyltransferase	Cytoplasmic	9.97
182	Mbov_0371[a,b]	AFM51733.1	alanyl-tRNA synthetase	Cytoplasmic	9.97
183	Mbov_0374[b]	AFM51736.1	putative lipoprotein	Unknown	
184	Mbov_0375[a,b]	AFM51737.1	oxidoreductase	Cytoplasmic	9.97
185	Mbov_0376[a]	AFM51738.1	phage-associated protein	Unknown	
186	Mbov_0393[a,b]	AFM51750.1	putative membrane lipoprotein (ICEB-1 encoded)	Unknown	
187	Mbov_0400[a,b]	AFM51757.1	hypothetical protein (ICEB-1 encoded)	Unknown	
188	Mbov_0403[a,b]	AFM51760.1	transcription elongation factor	Cytoplasmic	9.97
189	Mbov_0404[a,b]	AFM51761.1	hypoxanthine phosphoribosyltransferase	Cytoplasmic	9.97
190	Mbov_0408[a,b]	AFM51765.1	translation-associated GTPase	Cytoplasmic	7.50
191	Mbov_0411[a,b]	AFM51768.1	excinuclease ABC subunit A	Cytoplasmic	7.50
192	Mbov_0412[a,b]	AFM51769.1	excinuclease ABC subunit B	Cytoplasmic	9.97
193	Mbov_0418[a,b]	AFM51775.2	putative transmembrane protein	CytoplasmicMembrane	9.87
194	Mbov_0419[a,b]	AFM51776.1	Hypothetical protein Mbov_0419	Cytoplasmic	7.50
195	Mbov_0421[a,b]	AFM51778.1	trk system potassium uptake protein	Cytoplasmic	7.50
196	Mbov_0422[a,b]	AFM51779.2	cell division protein	Cytoplasmic	7.50
197	Mbov_0425[a,b]	AFM51782.1	cell division protein	Unknown	
198	Mbov_0426[a,b]	AFM51783.1	glycine cleavage system H protein	Cytoplasmic	7.50
199	Mbov_0427[a,b]	AFM51784.1	ribosomal RNA small subunit methyltransferase	Cytoplasmic	7.50

200	Mbov_0428[a,b]	AFM51785.1	endopeptidase O	Cytoplasmic	9.67
201	Mbov_0429[a,b]	AFM51786.1	single-strand DNA-binding protein	Cytoplasmic	9.67
202	Mbov_0430[a,b]	AFM51787.1	Uracil phosphoribosyltransferase	Unknown	
203	Mbov_0432[a,b]	AFM51789.1	leucyl-tRNA synthetase	Cytoplasmic	9.67
204	Mbov_0433[a,b]	AFM51790.1	ATP-dependent Lon protease	Cytoplasmic	9.97
205	Mbov_0438[a,b]	AFM51795.1	ATP synthase subunit beta	CytoplasmicMembrane	9.96
206	Mbov_0440[a,b]	AFM51797.1	ATP synthase subunit alpha	Cytoplasmic	9.97
207	Mbov_0446[a,b]	AFM51803.1	hypothetical protein Mbov_0446	Cytoplasmic	7.50
208	Mbov_0449[a,b]	AFM51806.2	putative membrane lipoprotein	Unknown	
209	Mbov_0451[a,b]	AFM51808.1	threonyl-tRNA synthetase	Cytoplasmic	10.00
210	Mbov_0452[a,b]	AFM51809.1	tryptophanyl-tRNA synthetase	Cytoplasmic	9.97
211	Mbov_0458[a,b]	AFM51815.1	variable surface lipoprotein	Unknown	
212	Mbov_0461[a,b]	AFM51818.1	putative membrane lipoprotein	Unknown	
213	Mbov_0462[a,b]	AFM51819.1	Putative lipoprotein	Unknown	
214	Mbov_0467[b]	AFM51823.1	putative transmembrane protein	Unknown	
215	Mbov_0468[a,b]	AFM51824.1	putative lipoprotein	Unknown	
216	Mbov_0469[a,b]	AFM51825.1	variable lipoprotein VspX	Unknown	
217	Mbov_0471[a,b]	AFM51827.1	Periplasmic protease	Unknown	
218	Mbov_0473[a,b]	AFM51829.1	putative lipoprotein	Extracellular	9.13
219	Mbov_0478[a,b]	AFM51834.1	O-sialoglycoprotein endopeptidase	Unknown	
220	Mbov_0479[a]	AFM51835.1	putative molecular chaperone	Unknown	
221	Mbov_0480[a,b]	AFM51836.1	putative ATPase or kinase	Cytoplasmic	7.50
222	Mbov_0481[a,b]	AFM51837.1	elongation factor	Cytoplasmic	9.97
223	Mbov_0482[a,b]	AFM51838.1	enolase	Cytoplasmic	9.97
224	Mbov_0485[a,b]	AFM51841.1	glucose-6-phosphate isomerase	Cytoplasmic	9.67
225	Mbov_0486[a]	AFM51842.1	formamidopyrimidine-DNA glycosylase	Cytoplasmic	7.50
226	Mbov_0487[a]	AFM51843.1	Hypothetical protein Mbov_0487	Unknown	
227	Mbov_0488[a,b]	AFM51844.1	peptide methionine sulfoxide reductase	Cytoplasmic	7.50
228	Mbov_0492[a,b]	AFM51848.1	putative transmembrane protein	Unknown	
229	Mbov_0493[a,b]	AFM51849.1	elongation factor	Cytoplasmic	9.97
230	Mbov_0494[a,b]	AFM51850.1	Hypothetical protein Mbov_0494	Unknown	
231	Mbov_0495[a,b]	AFM51851.1	Mg2+ transport protein	CytoplasmicMembrane	10.00
232	Mbov_0499[a,b]	AFM51855.1	asparaginyl-tRNA synthetase	Cytoplasmic	10.00
233	Mbov_0500[a,b]	AFM51856.1	Glycosyltransferase	CytoplasmicMembrane	8.16

234	Mbov_0502[a,b]	AFM51858.1	ATP-dependent DNA helicase	Cytoplasmic	9.97
235	Mbov_0504[a,b]	AFM51860.1	hypothetical protein Mbov_0504	Cytoplasmic	7.50
236	Mbov_0505[a,b]	AFM51861.1	putative lipoprotein	Unknown	
237	Mbov_0508[a,b]	AFM51862.1	ATPase subunit beta	CytoplasmicMembrane	9.51
238	Mbov_0509[a,b]	AFM51863.1	ATPase subunit alpha	Cytoplasmic	9.67
239	Mbov_0511[a,b]	AFM51865.1	hypothetical protein Mbov_0511	Unknown	
240	Mbov_0513[a,b]	AFM51867.1	hypothetical protein Mbov_0513	Unknown	
241	Mbov_0515[a,b]	AFM51869.1	putative lipoprotein	Unknown	
242	Mbov_0516[a,b]	AFM51870.1	Putative transmembrane protein	Unknown	
243	Mbov_0517[a,b]	AFM51871.1	Putative transmembrane protein	Unknown	
244	Mbov_0518[b]	AFM51872.1	putative lipoprotein	Unknown	
245	Mbov_0519[a,b]	AFM51873.1	Putative transmembrane protein	Unknown	
246	Mbov_0520[a,b]	AFM51874.1	DNA ligase	Cytoplasmic	7.50
247	Mbov_0521[a]	AFM51875.1	putative transmembrane protein	CytoplasmicMembrane	9.55
248	Mbov_0522[a,b]	AFM51876.1	phosphopentomutase	Cytoplasmic	9.97
249	Mbov_0528[a,b]	AFM51882.1	DNA polymerase III subunit delta	CytoplasmicMembrane	9.55
250	Mbov_0529[a,b]	AFM51883.1	50S ribosomal protein	Cytoplasmic	7.50
251	Mbov_0530[a]	AFM51884.1	30S ribosomal protein	Cytoplasmic	9.64
252	Mbov_0531[a]	AFM51885.1	Hypothetical protein Mbov_0531	Unknown	
253	Mbov_0532[a,b]	AFM51886.1	UTP--glucose-1-phosphate uridylyltransferase	Cytoplasmic	7.50
254	Mbov_0534[a,b]	AFM51888.1	ABC transporter permease protein	Cytoplasmic Membrane	10.00
255	Mbov_0535[a,b]	AFM51889.1	ABC-2 type transporter ATP-binding protein	Cytoplasmic Membrane	8.78
256	Mbov_0536[a,b]	AFM51890.1	putative lipoprotein	Unknown	
257	Mbov_0537[a,b]	AFM51891.1	putative lipoprotein	CytoplasmicMembrane	9.55
258	Mbov_0539[a,b]	AFM51893.1	phosphomannomutase	Cytoplasmic	7.50
259	Mbov_0541[a,b]	AFM51895.1	transcription termination factor	Unknown	
260	Mbov_0542[a,b]	AFM51896.1	Acyl carrier protein phosphodiesterase	Unknown	
261	Mbov_0543[a,b]	AFM51897.1	phosphocarrier protein	Cytoplasmic	10.00
262	Mbov_0548[a,b]	AFM51902.1	putative lipoprotein	Unknown	
263	Mbov_0549[a,b]	AFM51903.1	translation initiation factor	Cytoplasmic	9.97
264	Mbov_0551[b]	AFM51905.1	50S ribosomal protein	Cytoplasmic	9.67
265	Mbov_0552[a,b]	AFM51906.1	ATP-binding protein	CytoplasmicMembrane	9.55

266	Mbov_0553[a]	AFM51907.1	putative transmembrane protein	CytoplasmicMembrane	10.00
267	Mbov_0554[a]	AFM51908.1	PTS lichenan-specific IIA component LicA	Unknown	
268	Mbov_0555[a,b]	AFM51909.1	GTP-binding protein	CytoplasmicMembrane	8.78
269	Mbov_0557[a,b]	AFM51911.1	NADPH flavin oxidoreductase	Cytoplasmic	7.50
270	Mbov_0558[a,b]	AFM51912.1	triacylglycerol lipase	Unknown	
271	Mbov_0563[a,b]	AFM51917.2	arginyl-tRNA synthetase	Cytoplasmic	9.97
272	Mbov_0565[a,b]	AFM51919.1	L-lactate dehydrogenase	Cytoplasmic	9.97
273	Mbov_0566[a,b]	AFM51920.1	pyruvate dehydrogenase E2 component	Unknown	
274	Mbov_0567[a,b]	AFM51921.1	phosphate acetyltransferase	Cytoplasmic	7.50
275	Mbov_0568[a,b]	AFM51922.1	acetate kinase	Cytoplasmic	9.97
276	Mbov_0570[a,b]	AFM51924.1	putative lipoprotein	Unknown	
277	Mbov_0571[a,b]	AFM51925.1	hexosephosphate transport protein	CytoplasmicMembrane	10.00
278	Mbov_0574[a,b]	AFM51928.1	deoxyribonuclease IV	Cytoplasmic	7.50
279	Mbov_0576[a,b]	AFM51930.1	hioredoxin	Unknown	
280	Mbov_0579[a,b]	AFM51933.1	membrane lipoprotein P81	Unknown	
281	Mbov_0580[a,b]	AFM51934.1	nuclease	Extracellular	9.97
282	Mbov_0581[a,b]	AFM51935.1	multiple sugar ABC transporter ATP-binding protein	CytoplasmicMembrane	9.51
283	Mbov_0585[a,b]	AFM51939.1	putative lipoprotein	Unknown	
284	Mbov_0586[a,b]	AFM51940.1	GTP-binding protein	Cytoplasmic	7.50
285	Mbov_0587[a,b]	AFM51941.1	putative hydrolases of the HAD superfamily	Cytoplasmic	7.50
286	Mbov_0588[a,b]	AFM51942.1	purine-nucleoside phosphorylase	Cytoplasmic	7.50
287	Mbov_0589[a,b]	AFM51943.1	thymidine phosphorylase	Cytoplasmic	7.50
288	Mbov_0590[a,b]	AFM51944.1	deoxyribose-phosphate aldolase	Cytoplasmic	9.97
289	Mbov_0591[a,b]	AFM51945.1	triosephosphate isomerase	Cytoplasmic	9.97
290	Mbov_0594[a,b]	AFM51948.1	cobalt/nickel ABC transporter ATP-binding protein	CytoplasmicMembrane	8.78
291	Mbov_0596[a,b]	AFM51950.1	50S ribosomal protein	Cytoplasmic	9.67
292	Mbov_0597[a,b]	AFM51951.1	DNA-directed RNA polymerase subunit alpha	Cytoplasmic	9.97
293	Mbov_0598[a,b]	AFM51952.1	30S ribosomal protein	Cytoplasmic	9.67
294	Mbov_0599[a,b]	AFM51953.1	30S ribosomal protein	Cytoplasmic	9.97
295	Mbov_0600[a,b]	AFM51954.1	Ribosomal protein	Cytoplasmic	9.67
296	Mbov_0601[a,b]	AFM51955.1	translation initiation factor	Cytoplasmic	9.97
297	Mbov_0602[a,b]	AFM51956.1	methionyl aminopeptidase	Cytoplasmic	9.97
298	Mbov_0603[a,b]	AFM51957.1	adenylate kinase	Cytoplasmic	9.97
299	Mbov_0606[a,b]	AFM51959.1	glucose-1-phosphatase	CytoplasmicMembrane	8.16

300	Mbov_0611[a]	AFM51962.1	50S ribosomal protein	Unknown	
301	Mbov_0612[a,b]	AFM51963.1	30S ribosomal protein	Cytoplasmic	9.67
302	Mbov_0613[a,b]	AFM51964.1	50S ribosomal protein	Cytoplasmic	9.97
303	Mbov_0614[a,b]	AFM51965.1	50S ribosomal protein	Cytoplasmic	7.50
304	Mbov_0617[a,b]	AFM51968.1	50S ribosomal protein	Cytoplasmic	7.50
305	Mbov_0618[a,b]	AFM51969.1	50S ribosomal protein	Cytoplasmic	9.67
306	Mbov_0619[a,b]	AFM51970.1	50S ribosomal protein	Cytoplasmic	9.97
307	Mbov_0620[a,b]	AFM51971.1	30S ribosomal protein	Cytoplasmic	9.97
308	Mbov_0621[a,b]	AFM51972.1	50S ribosomal protein	Unknown	
309	Mbov_0622[a]	AFM51973.1	50S ribosomal protein	Cytoplasmic	9.97
310	Mbov_0623[a,b]	AFM51974.1	30S ribosomal protein	Cytoplasmic	9.97
311	Mbov_0624[a,b]	AFM51975.1	50S ribosomal protein	Cytoplasmic	9.67
312	Mbov_0625[a,b]	AFM51976.1	30S ribosomal protein	Cytoplasmic	9.97
313	Mbov_0626[a,b]	AFM51977.1	50S ribosomal protein	Unknown	
314	Mbov_0627[a,b]	AFM51978.1	50S ribosomal protein	Cytoplasmic	9.67
315	Mbov_0628[a]	AFM51979.1	50S ribosomal protein	Unknown	
316	Mbov_0629[a,b]	AFM51980.1	50S ribosomal protein	Cytoplasmic	9.97
317	Mbov_0630[a,b]	AFM51981.1	30S ribosomal protein	Cytoplasmic	9.67
318	Mbov_0631[b]	AFM51982.1	DNA-binding protein HU-beta	Cytoplasmic	7.50
319	Mbov_0634[a,b]	AFM51985.1	hypothetical protein Mbov_0634	Unknown	
320	Mbov_0635[a,b]	AFM51986.1	endoglucanase	Cytoplasmic	7.50
321	Mbov_0636[a]	AFM51987.1	16S rRNA pseudouridine synthase	Cytoplasmic	9.97
322	Mbov_0637[a,b]	AFM51988.1	50S ribosomal protein	Cytoplasmic	9.67
323	Mbov_0638[a,b]	AFM51989.1	50S ribosomal protein	Cytoplasmic	7.50
324	Mbov_0641[a,b]	AFM51992.1	heat shock protein GrpE	Cytoplasmic	7.50
325	Mbov_0642[a,b]	AFM51993.1	heat-inducible transcriptional repressor	Unknown	
326	Mbov_0643[a]	AFM51994.1	50S ribosomal protein	Cytoplasmic	9.67
327	Mbov_0644[a,b]	AFM51995.1	30S ribosomal protein	Unknown	
328	Mbov_0646[a,b]	AFM51997.1	DNA gyrase subunit A	Cytoplasmic	9.97
329	Mbov_0647[a]	AFM51998.1	superfamily I DNA and RNA helicase	Unknown	
330	Mbov_0650[a,b]	AFM52001.1	type I restriction enzyme R subunit	Cytoplasmic	7.50
331	Mbov_0651[a,b]	AFM52002.1	type I restriction enzyme S subunit	Unknown	
332	Mbov_0652[a,b]	AFM52003.1	type I restriction enzyme M protein	Unknown	
333	Mbov_0654[a,b]	AFM52005.1	putative lipoprotein	Unknown	

334	Mbov_0656[a,b]	AFM52007.1	putative lipoprotein	Unknown	
335	Mbov_0658[a,b]	AFM52009.1	Periplasmic protease	Unknown	
336	Mbov_0660[a,b]	AFM52011.1	glutamyl-tRNA synthetase	Cytoplasmic	9.97
337	Mbov_0661[a,b]	AFM52012.1	hypothetical protein Mbov_0661	Unknown	
338	Mbov_0663[a,b]	AFM52014.1	S-adenosylmethionine synthetase	Cytoplasmic	9.97
339	Mbov_0665[a]	AFM52016.1	signal recognition particle subunit	CytoplasmicMembrane	8.78
340	Mbov_0666[a,b]	AFM52017.1	putative acetyltransferase	Unknown	
341	Mbov_0667[a,b]	AFM52018.1	Hypothetical protein Mbov_0667	Unknown	
342	Mbov_0668[a,b]	AFM52019.1	nicotinate phosphoribosyltransferase	Cytoplasmic	7.50
343	Mbov_0669[a,b]	AFM52020.1	phosphoglycerate kinase	Cytoplasmic	9.97
344	Mbov_0673[a,b]	AFM52023.1	leucyl aminopeptidase	Cytoplasmic	9.67
345	Mbov_0674[a,b]	AFM52024.1	putative lipoprotein	Unknown	
346	Mbov_0675[a,b]	AFM52025.1	5'nucleotidase	Cell wall	9.21
347	Mbov_0676[a,b]	AFM52026.1	elongation factor	Cytoplasmic	9.97
348	Mbov_0677[a,b]	AFM52027.1	30S ribosomal protein	Cytoplasmic	7.50
349	Mbov_0678[a,b]	AFM52028.1	30S ribosomal protein	Cytoplasmic	9.67
350	Mbov_0679[a,b]	AFM52029.1	hypothetical protein Mbov_0679	Cytoplasmic	7.50
351	Mbov_0680[a,b]	AFM52030.1	drug resistance ATPase ABC transporter family ATP-binding protein	Cytoplasmic	9.97
352	Mbov_0687[a,b]	AFM52036.1	ATP-binding cassette subfamily B	CytoplasmicMembrane	9.99
353	Mbov_0691[a,b]	AFM52040.1	endoglucanase	Cytoplasmic	7.50
354	Mbov_0693[a,b]	AFM52042.1	putative transmembrane protein	Extracellular	8.91
355	Mbov_0694[a,b]	AFM52043.1	DNA-directed RNA polymerase subunit beta'	Cytoplasmic	9.97
356	Mbov_0695[a,b]	AFM52044.1	DNA-directed RNA polymerase subunit beta	Cytoplasmic	9.97
357	Mbov_0696[a,b]	AFM52045.1	putative lipoprotein	Unknown	
358	Mbov_0697[a,b]	AFM52046.1	50S ribosomal protein	Cytoplasmic	9.67
359	Mbov_0698[a,b]	AFM52047.1	50S ribosomal protein	Cytoplasmic	9.97
360	Mbov_0700[a,b]	AFM52049.1	lysyl-tRNA synthetase	Cytoplasmic	10.00
361	Mbov_0701[a,b]	AFM52050.1	hypothetical protein Mbov_0701	Cytoplasmic	7.50
362	Mbov_0702[a,b]	AFM52051.1	Transcriptional accessory protein	Cytoplasmic	7.50
363	Mbov_0703[a,b]	AFM52052.1	Clp protease ATP-binding subunit	Cytoplasmic	9.97
364	Mbov_0710[a,b]	AFM52059.1	ribosomal protein small subunit S16	Cytoplasmic	9.97
365	Mbov_0711[a,b]	AFM52060.1	tRNA-methyltransferase	Cytoplasmic	7.50

366	Mbov_0712[a,b]	AFM52061.1	50S ribosomal protein	Cytoplasmic	9.97
367	Mbov_0713[a,b]	AFM52062.1	methionyl-tRNA formyltransferase	Cytoplasmic	9.97
368	Mbov_0715[a,b]	AFM52064.1	phosphotransferase system enzyme I	Cytoplasmic	9.97
369	Mbov_0718[a]	AFM52067.1	L-ribulose-5-phosphate 4-epimerase	Cytoplasmic	7.50
370	Mbov_0719[a]	AFM52068.1	L-ribulose-5-phosphate 3-epimerase	Cytoplasmic	7.50
371	Mbov_0720[a,b]	AFM52069.1	3-dehydro-L-gulonate-6-phosphate decarboxylase	Cytoplasmic	7.50
372	Mbov_0721[a]	AFM52070.1	ascorbate-specific PTS system enzyme II Ccomponent	Unknown	
373	Mbov_0722[a]	AFM52071.2	ascorbate-specific PTS system enzyme II Bcomponent	Unknown	
374	Mbov_0724[a]	AFM52073.1	putative metal-dependent hydrolase with the TIM-barrel fold	CytoplasmicMembrane	8.78
375	Mbov_0725[a,b]	AFM52074.1	putative hydrolases of the HAD superfamily	Unknown	
376	Mbov_0732[a]	AFM52081.1	putative lipoprotein	Extracellular	8.91
377	Mbov_0734[a]	AFM52083.1	hypothetical protein Mbov_0734	Cytoplasmic	7.50
378	Mbov_0739[a,b]	AFM52087.1	putative lipoprotein	Unknown	
379	Mbov_0743[a,b]	AFM52091.1	putative transmembrane protein	Unknown	
380	Mbov_0745[a,b]	AFM52093.1	hypothetical protein Mbov_0745	Cytoplasmic	7.50
381	Mbov_0746[a]	AFM52094.1	NAD(+) synthetase [	Cytoplasmic	7.50
382	Mbov_0747[a,b]	AFM52095.1	Hypothetical protein Mbov_0747	Unknown	
383	Mbov_0749[a]	AFM52097.1	methyltransferase	Cytoplasmic	7.50
384	Mbov_0750[a]	AFM52098.1	DNA replication protein	Unknown	
385	Mbov_0755[a,b]	AFM52103.1	putative adenine-specific DNA-methyltransferase	Cytoplasmic	7.50
386	Mbov_0758[a]	AFM52106.1	putative ATPase	Cytoplasmic	7.50
387	Mbov_0760[a,b]	AFM52108.1	putative transmembrane protein	CytoplasmicMembrane	10.00
388	Mbov_0761[a,b]	AFM52109.1	ribosomal large subunit pseudouridine synthase D	Cytoplasmic	9.97
389	Mbov_0765[a,b]	AFM52113.1	hypothetical protein Mbov_0765	Cytoplasmic	7.50
390	Mbov_0768[a,b]	AFM52116.1	chromosome segregation protein	Unknown	
391	Mbov_0771[a,b]	AFM52119.1	thymidylate kinase	Cytoplasmic	7.50
392	Mbov_0772[a,b]	AFM52120.1	recombination protein	Cytoplasmic	7.50
393	Mbov_0773[a,b]	AFM52121.1	Hypothetical protein Mbov_0773	Cytoplasmic	7.50
394	Mbov_0774[a,b]	AFM52122.1	DNA polymerase III subunit gamma/tau	Cytoplasmic	9.97
395	Mbov_0776[a,b]	AFM52124.1	phosphoglycerate mutase	Cytoplasmic	9.97
396	Mbov_0777[a,b]	AFM52125.1	putative hydrolases of the HAD superfamily	Cytoplasmic	7.50
397	Mbov_0778[a,b]	AFM52126.1	putative transmembrane protein	CytoplasmicMembrane	10.00
398	Mbov_0779[a]	AFM52127.1	putative transmembrane protein	CytoplasmicMembrane	9.55

399	Mbov_0783[a,b]	AFM52131.1	adenine phosphoribosyltransferase	Cytoplasmic	9.97
400	Mbov_0784[a,b]	AFM52132.1	translation initiation factor	Cytoplasmic	9.97
401	Mbov_0786[a,b]	AFM52134.1	transcription elongation factor	Cytoplasmic	7.50
402	Mbov_0787[a,b]	AFM52135.1	hypothetical protein Mbov_0787	Cytoplasmic	7.50
403	Mbov_0788[a,b]	AFM52136.1	thymidine kinase	Cytoplasmic	9.97
404	Mbov_0789[a,b]	AFM52137.1	leucyl aminopeptidase	Cytoplasmic	9.64
405	Mbov_0793[a,b]	AFM52141.1	variable surface lipoprotein VspHB0801-1	Unknown	
406	Mbov_0794[a,b]	AFM52142.1	variable surface lipoprotein VspHB0801-2	CytoplasmicMembrane	9.87
407	Mbov_0795[a]	AFM52143.1	variable surface lipoprotein VspHB0801-3	CytoplasmicMembrane	9.87
408	Mbov_0796[a,b]	AFM52144.1	variable surface lipoprotein VspHB0801-4	Unknown	
409	Mbov_0797[a,b]	AFM52145.1	variable surface lipoprotein VspHB0801-5	Unknown	
410	Mbov_0798[a,b]	AFM52146.1	variable surface lipoprotein VspHB0801-6	Unknown	
411	Mbov_0802[a,b]	AFM52150.1	ribonuclease J	Cytoplasmic	7.50
412	Mbov_0804[a,b]	AFM52152.2	hypothetical protein Mbov_0804	Unknown	
413	Mbov_0806[a,b]	AFM52154.1	aspartyl/glutamyl-tRNA amidotransferase subunit B	Cytoplasmic	7.50
414	Mbov_0807[a,b]	AFM52155.1	aspartyl/glutamyl-tRNA amidotransferase subunit A	Cytoplasmic	9.67
415	Mbov_0808[a,b]	AFM52156.1	aspartyl/glutamyl-tRNA amidotransferase subunit C	Cytoplasmic	7.50
316	Mbov_0810[a,b]	AFM52158.1	segregation and condensation protein	Cytoplasmic	9.97
417	Mbov_0811[a,b]	AFM52159.1	segregation and condensation protein	Cytoplasmic	9.97
418	Mbov_0813[a,b]	AFM52161.1	holo-[acyl-carrier protein] synthase	Cytoplasmic	7.50
419	Mbov_0814[a,b]	AFM52162.1	GTP-binding protein	Cytoplasmic	9.97
420	Mbov_0815[a,b]	AFM52163.1	cytidylate kinase	Cytoplasmic	9.97
421	Mbov_0817[a,b]	AFM52165.1	heat shock protein	Cytoplasmic	9.67
422	Mbov_0818[a,b]	AFM52166.2	ribosome-binding factor A	Cytoplasmic	9.67
423	Mbov_0819[a,b]	AFM52167.1	tyrosyl-tRNA synthetase	Cytoplasmic	9.97
424	Mbov_0820[a,b]	AFM52168.1	EMAP domain protein	Cytoplasmic	9.67
425	Mbov_0821[a,b]	AFM52169.1	hypothetical protein Mbov_0821	Unknown	
426	Mbov_0822[a,b]	AFM52170.1	Acyl carrier protein	Unknown	
427	Mbov_0823[a,b]	AFM52171.1	protoporphirogen oxidase HEMK	Cytoplasmic	7.50
428	Mbov_0824[a,b]	AFM52172.2	peptide chain release factor	Cytoplasmic	9.97
429	Mbov_0825[a,b]	AFM52173.1	cell filamentation protein	Cytoplasmic	7.50
430	Mbov_0831[a,b]	AFM52176.1	DNA gyrase subunit B	Cytoplasmic	9.67
431	Mbov_0832[a,b]	AFM52177.1	Thioredoxin	Cytoplasmic	9.67
432	Mbov_0833[a]	AFM52178.1	SsrA-binding protein	Unknown	

433	Mbov_0838[a,b]	AFM52183.1	putative lipoprotein	Unknown	
434	Mbov_0839[a]	AFM52184.1	LacI family transcriptional regulator	Cytoplasmic	9.97
435	Mbov_0843[a]	AFM52188.1	ABC transporter ATP-binding protein	CytoplasmicMembrane	9.51
436	Mbov_0845[a,b]	AFM52189.1	cell division protease	CytoplasmicMembrane	10.00
437	Mbov_0847[a,b]	AFM52191.1	peptidyl-tRNA hydrolase	Cytoplasmic	9.97
438	Mbov_0848[a]	AFM52192.1	exodeoxyribonuclease V alpha subunit	Cytoplasmic	7.50

a: related to *M. bovis* HB0801-P1, b: related to P150 and a,b: related to both (*M. bovis* HB0801-P1, and P150).

Table S3 A total of 59 extracellular proteins were identified from *M. bovis* secretome by using using PRID-LIPO signal peptide prediction

No	Proteins ID			Signal peptides		
	Mnemonic	Accession No	Protein Name	Signal peptide	Most likely cleavage	Reliabil
1	Mbov_0016[a,b]	AFM51394_1	P48-like surface lipoprotein	Lipo signal peptide predicted	1 - 24 [AAS-CG]	1.000
2	Mbov_0037[a,b]	AFM51415_2	oligopeptide ABC transporter substrate-binding protein	Lipo signal peptide predicted	1 - 26 [AAK-CG]	0.880
3	Mbov_0038[a,b]	AFM51416_1	putative transmembrane protein	TM segment predicted	14 - 35	0.696
4	Mbov_0049[a,b]	AFM51424_1	putative lipoprotein	Lipo signal peptide predicted	1 - 22 [SSS-CF]	0.711
5	Mbov_0111[a,b]	AFM51486_1	putative lipoprotein	Lipo signal peptide predicted	1 - 27 [AAR-CQ]	0.915
6	Mbov_0154[a,b]	AFM51527_1	putative transmembrane protein	TM segment predicted:	9 - 30	0.739
7	Mbov_0156[a,b]	AFM51529_1	putative lipoprotein	Lipo signal peptide predicted	1 - 25 [AAK-CV]	0.978
8	Mbov_0211[b]	AFM51580_1	Endonuclease I	Lipo signal peptide predicted	1 - 32 [STG-CN]	0.998
9	Mbov_0217[a,b]	AFM51586_1	putative lipoprotein	Lipo signal peptide predicted	1 - 26 [SAK-CG]	0.956
10	Mbov_0274[a]	AFM51642_1	putative lipoprotein	Lipo signal peptide predicted	1 - 23 [AAS-CQ]	1.000

11	Mbov_0283[b]	AFM51651_1	putative lipoprotein	Sec signal peptide predicted	1 - 31 [AKA-DN]	0.982
12	Mbov_0290[b]	AFM51658_1	putative lipoprotein	Lipo signal peptide predicted	1 - 20 [AAA-CD]	0.999
13	Mbov_0296[b]	AFM51664_1	putative lipoprotein	Sec signal peptide predicted	1 - 25 [AKC-FG]	0.919
14	Mbov_0326[b]	AFM51694_1	putative secreted acid phosphatase	Lipo signal peptide predicted	1 - 26 [AAT-CE]	0.840
15	Mbov_0339[b]	AFM51707_1	variable surface lipoprotein VspY1	Lipo signal peptide predicted	1 - 23 [AAS-CD]	0.993
16	Mbov_0341[b]	AFM51709_1	putative transmembrane protein	Sec signal peptide predicted	1 - 32 [PFG-DK]	0.916
17	Mbov_0350[a,b]	AFM51716_1	putative lipoprotein	Sec signal peptide predicted	1 - 27 [SIS-KP]	0.800
18	Mbov_0364[a,b]	AFM51726_1	putative membrane lipoprotein	Sec signal peptide predicted	1 - 23 [AIA-VP]	0.995
19	Mbov_0368[b]	AFM51730_1	putative transmembrane protein	Sec signal peptide predicted	1 - 33 [VIK-SN]	0.967
20	Mbov_0374[b]	AFM51736_1	putative lipoprotein	Lipo signal peptide predicted	1 - 28 [VLS-CT]	0.539
21	Mbov_0393[a,b]	AFM51750_1	putative membrane lipoprotein (ICEB-1 encoded)	TM segment predicted	6 - 28	0.925

22	Mbov_0449[a,b]	AFM51806_2	putative membrane lipoprotein	Sec signal peptide predicted	1 - 31 [LIA-AK]	0.981
23	Mbov_0458[a,b]	AFM51815_1	variable surface lipoprotein	Lipo signal peptide predicted	1 - 24 [ATS-CG]	0.999
24	Mbov_0461[a,b]	AFM51818_1	putative membrane lipoprotein	Sec signal peptide predicted	1 - 24 [VVA-AS]	0.949
25	Mbov_0462[a,b]	AFM51819_1	Putative lipoprotein	Lipo signal peptide predicted	1 - 26 [AAS-CD]	0.891
26	Mbov_0467[b]	AFM51823_1	putative transmembrane protein	TM segment predicted	6 - 27	0.876
27	Mbov_0468[a,b]	AFM51824_1	putative lipoprotein	Lipo signal peptide predicted	1 - 24 [LTS-CS]	1.000
28	Mbov_0469[a,b]	AFM51825_1	variable lipoprotein VspX	Lipo signal peptide predicted	1 - 25 [AAS-CG]	1.000
29	Mbov_0471[a,b]	AFM51827_1	Periplasmic protease	Sec signal peptide predicted	1 - 32 [ADF-DE]	0.921
30	Mbov_0473[a,b]	AFM51829_1	putative lipoprotein	Lipo signal peptide predicted	1 - 26 [AAS-CE]	1.000
31	Mbov_0505[a,b]	AFM51861_1	putative lipoprotein	Sec signal peptide predicted	1 - 33 [AYN-IS]	0.925
32	Mbov_0515[a,b]	AFM51869_1	putative lipoprotein	Lipo signal peptide predicted	1 - 25 [AAK-CN]	0.974

33	Mbov_0516[a,b]	AFM51870_1	Putative transmembrane protein	Sec signal peptide predicted	1 - 32 [IYN-AS]	0.982
34	Mbov_0517[a,b]	AFM51871_1	Putative transmembrane protein	Sec signal peptide predicted	1 - 32 [IYK-AT]	0.979
35	Mbov_0518[b]	AFM51872_1	putative lipoprotein	Sec signal peptide predicted	1 - 22 [LIA-AK]	0.947
36	Mbov_0519[a,b]	AFM51873_1	Putative transmembrane protein	TM segment predicted	11 - 31	0.591
37	Mbov_0536[a,b]	AFM51890_1	putative lipoprotein	Lipo signal peptide predicted	1 - 23 [LAS-CE]	0.974
38	Mbov_0537[a,b]	AFM51891_1	putative lipoprotein	Lipo signal peptide predicted	1 - 23 [SVS-CI]	0.884
39	Mbov_0570[a,b]	AFM51924_1	putative lipoprotein	Lipo signal peptide predicted	1 - 27 [SAR-CN]	0.927
40	Mbov_0579[a,b]	AFM51933_1	membrane lipoprotein P81	Lipo signal peptide predicted	1 - 25 [SAK-CG]	0.961
41	Mbov_0580[a,b]	AFM51934_1	Nuclease	Sec signal peptide predicted	1 - 33 [IKA-KE]	0.993
42	Mbov_0585[a,b]	AFM51939_1	putative lipoprotein	Sec signal peptide predicted	1 - 27 [ASC-IH]	0.948
43	Mbov_0654[a,b]	AFM52005_1	putative lipoprotein	Lipo signal peptide predicted	1 - 25 [AAS-CE]	0.993

44	Mbov_0656[a,b]	AFM52007_1	putative lipoprotein (variable)	No signal peptide predicted		0.559
45	Mbov_0658[a,b]	AFM52009_1	Periplasmic protease	Sec signal peptide predicted	1 - 28 [SNS-NS]	0.984
46	Mbov_0674[a,b]	AFM52024_1	putative lipoprotein	TM segment predicted	18 - 39	0.973
47	Mbov_0675[a,b]	AFM52025_1	5'nucleotidase	Lipo signal peptide predicted	1 - 24 [AAT-CG]	0.965
48	Mbov_0693[a,b]	AFM52042_1	putative transmembrane protein	TM segment predicted	9 - 30	0.684
49	Mbov_0696[a,b]	AFM52045_1	putative lipoprotein	Sec signal peptide predicted	1 - 31 [NSA-SE]	0.988
50	Mbov_0732[a]	AFM52081_1	putative lipoprotein	No signal peptide predicted		1.000
51	Mbov_0739[a,b]	AFM52087_1	putative lipoprotein	Lipo signal peptide predicted	1 - 23 [VVA-CG]	1.000
52	Mbov_0743[a,b]	AFM52091_1	putative transmembrane protein	TM segment predicted:	17 - 34	0.576
53	Mbov_0768[a,b]	AFM52116_1	chromosome segregation protein	Sec signal peptide predicted	1 - 24 [VFV-AV]	0.955
54	Mbov_0793[a,b]	AFM52141_1	variable surface lipoprotein VspHB0801-1	Lipo signal peptide predicted	1 - 24 [AAK-CG]	0.998
55	Mbov_0794[a,b]	AFM52142_1	variable surface lipoprotein VspHB0801-2	Lipo signal peptide predicted	1 - 24 [AAK-CG]	0.998

56	Mbov_0795[a]	AFM52143_1	variable surface lipoprotein VspHB0801-3	Lipo signal peptide predicted	1 - 24 [AAK-CG]	0.998
57	Mbov_0796[a,b]	AFM52144_1	variable surface lipoprotein VspHB0801-4	Lipo signal peptide predicted	1 - 24 [AAK-CG]	0.998
58	Mbov_0797[a,b]	AFM52145_1	variable surface lipoprotein VspHB0801-5	Lipo signal peptide predicted	1 - 24 [AAK-CG]	0.998
59	Mbov_0798[a,b]	AFM52146_1	variable surface lipoprotein VspHB0801-6	Lipo signal peptide predicted.	1 - 24 [AAK-CG]	0.998

a: related to *M. bovis* HB0801-P1, b: related to P150 and a,b: related to both (*M. bovis* HB0801-P1, and P150).

Table S4 Identification of linear B-cell epitopes of *M. bovis* secreted proteins.

No	Proteins		B cell epitopes	
	Mnemonic	Accession No	Over all B cell epitopes Numbers	Image length
1	Mbov_0016[a,b]	AFM51394_1	22	23
2	Mbov_0038[a,b]	AFM51416_1	161	30
3	Mbov_0049[a,b]	AFM51424_1	34	64
4	Mbov_0111[a,b]	AFM51486_1	53	31
5	Mbov_0154[a,b]	AFM51527_1	24	20
6	Mbov_0156[a,b]	AFM51529_1	14	29
7	Mbov_0217[a,b]	AFM51586_1	21	30
8	Mbov_0274[a]	AFM51642_1	35	17
9	Mbov_0283[b]	AFM51651_1	6	107
10	Mbov_0290[b]	AFM51658_1	26	29
11	Mbov_0296[b]	AFM51664_1	30	12
12	Mbov_0339[b]	AFM51707.1	7	185
13	Mbov_0350[a,b]	AFM51716_1	36	64
14	Mbov_0364[a,b]	AFM51726_1	25	24
15	Mbov_0368[b]	AFM51730_1	14	29
16	Mbov_0374[b]	AFM51736_1	38	40
17	Mbov_0449[a,b]	AFM51806_2	12	73
18	Mbov_0458[a,b]	AFM51815_1	6	159
19	Mbov_0461[a,b]	AFM51818_1	5	27
20	Mbov_0462[a,b]	AFM51819_1	8	80
21	Mbov_0468[a,b]	AFM51824_1	24	60
22	Mbov_0469[a,b]	AFM51825_1	4	93
23	Mbov_0471[a,b]	AFM51827_1	30	22
24	Mbov_0473[a,b]	AFM51829_1	9	73
25	Mbov_0505[a,b]	AFM51861_1	38	53
26	Mbov_0515[a,b]	AFM51869_1	52	34
27	Mbov_0516[a,b]	AFM51870_1	46	55
28	Mbov_0517[a,b]	AFM51871_1	42	28
29	Mbov_0518[b]	AFM51872_1	45	23
30	Mbov_0519[a,b]	AFM51873_1	41	34

31	Mbov_0536[a,b]	AFM51890_1	19	19
32	Mbov_0537[a,b]	AFM51891_1	13	114
33	Mbov_0570[a,b]	AFM51924_1	34	26
34	Mbov_0579[a,b]	AFM51933_1	39	21
35	Mbov_0580[a,b]	AFM51934_1	18	20
36	Mbov_0585[a,b]	AFM51939_1	18	38
37	Mbov_0654[a,b]	AFM52005_1	5	132
38	Mbov_0656[a,b]	AFM52007_1	5	129
39	Mbov_0658[a,b]	AFM52009_1	29	45
40	Mbov_0674[a,b]	AFM52024_1	18	48
41	Mbov_0675[a,b]	AFM52025_1	36	30
42	Mbov_0696[a,b]	AFM52045_1	6	57
43	Mbov_0739[a,b]	AFM52087_1	38	19
44	Mbov_0743[a,b]	AFM52091_1	48	23
45	Mbov_0768[a,b]	AFM52116_1	15	15
46	Mbov_0793[a,b]	AFM52141_1	0	Nil
47	Mbov_0794[a,b]	AFM52142_1	1	145
48	Mbov_0796[a,b]	AFM52144_1	15	60
49	Mbov_0797[a,b]	AFM52145_1	5	168
50	Mbov_0798[a,b]	AFM52146_1	22	21

a: related to *M. bovis* HB0801-P1, b: related to P150 and a,b: related to both (*M. bovis* HB0801-P1, and P150).

Table S5 Identification of affinity of T cell epitopes to MHC class I (nine-mers) of *M. bovis* secreted proteins by using IEDB-AR.

No	Proteins		Intermediate affinity binding		High affinity binding	
	Mnemonic	Accession No	Sequence	Position	Sequence	Position
			AAPVLSVPL	12-20		
			AVYNVLADL	349-357		
			CGFTHKAAL	139-147		
			KYVGVATSG	392-400		
			YYESKKAEI	420-428		
			GEPVAVAAI	280-288	GENEASKYV	386-394
			GEPAAKAFV	179-187	FDTKQAAYI	200-208
			ASYPVAGSL	296-304	IPWPAVSDF	236-244
1	Mbov_0016[a,b]	AFM51394_1	SSFRYIVLC	131-139	LEPEYIKKI	150-158
			LYSQGENSL	357-365	TEVDGVKTV	33-41
			AAYIAGRAL	205-213	KSLNNTIEL	265-273
			KYYESKKAE	419-427		
			RIFTSVMKL	337-345		
			AAPVLSVPL	12-20		
			NDEIANKAL	406-414		
			KFYLFLGAA	5-13		
			NEASKYVGV	388-396		

			YDKNAVKFV	2409-2417		
			SFNNSSTSL	1059-1067		
			SELISVETL	752-760		
			TEFYAYSKF	793-801		
			LETAFADPF	2598-2606	IEDLTSRFI	419-427
			AEDDEGKTI	912-920	FEAELSVFL	1287-1295
			KELTSYKTL	1719-1727	SEPELRKFL	224-232
			ANIGKYNAL	233-241	YSLENGKYL	2084-2092
			DEAELKNIF	50-58	SALLNSSSL	1402-1410
			EEKYKADAL	3028-3036	TAPINGEVL	1820-1828
			LESNLFKEL	1302-1310	IMRRFSSSL	2795-2803
			KYAISGGVL	13-21	LYLKARTHV	2173-2181
			FPDTSKADF	2658-2666	FDMWVINAI	2286-2294
			FEEVIKNGL	2350-2358	YSNVNGIEV	1597-1605
			WEIGAEDTI	2851-2859	GEWLEGLHI	2064-2072
			FAMFNSALT	2804-2812	QYIEAVSPI	126-134
			INEQFFRGI	249-257	SSIMNDAIV	1445-1453
2	Mbov_0038[a,b]	AFM51416_1	NEFEKLTKL	930-938	NESINNKSV	1537-1545
			SYFGEFQSI	3060-3068	KEIELGNKI	961-969
			YSPSNHDFG	2962-2970	VEQDIDKSI	1839-1847
			SSYTIHNAF	375-383	TEFNKKFNI	1684-1692
			AETVINITI	526-534	NYAGARKAI	477-485
			YVYDQFNGV	3317-3325	RGITNVRYL	255-263
			KAITLVDSL	2874-2882	GEVLFNSFV	1825-1833
			LNWRDFYNL	769-777	SEETLKNSL	440-448
			TERKFNELF	2934-2942	SENTSSNIL	2514-2522
			FSIVKGVEM	159-167	KEYTTIKFF	182-190
			KYIQFDKAL	404-412	KYYAKHHAL	103-111
			IMSKYRNAL	3241-3249	NELSNKFGI	658-666
			SETKFGSKF	1981-1989	VELGTKRLL	2181-2189
			KNLNKYIQF	400-408	WETKTNRDI	1737-1745
			TVTNMFSNI	2472-2480		
			NSLAKFSEL	446-454		
			FPDAFFGTL	190-198		

VALQQYELV	2899-2907
YNSTFLMQL	1022-1030
IEFYKFTEW	134-142
MFYYKTQNI	3183-3191
KVYEHILYV	3310-3318
VAYLPSELI	747-755
KYDKNSDTV	2725-2733
SVFLNYKNT	1292-1300
FDDGSSYTI	371-379
YEGLTDELL	2049-2057
NEIVDKALL	360-368
KELNRLKRV	1308-1316
NMPSFLSNI	2971-2979
IYENPRYYI	2530-2538
DYAAQYSLL	1093-1101
NEKLGRKNF	40-48
LSFLSSQNV	1035-1043
DYVNSKVDI	2738-2746
NEDYAAQYS	1091-1099
TMYMPNVKL	2590-2598
KYYAKHHAL	103-111
IEAELSKFK	1801-1809
EYAEIYNNL	1381-1389
YTYNFAEVI	2945-2953
RQITNENDV	1911-1919
IEFEDYKKW	2705-2713
IDILTKKKI	1929-1937
VEGIRKNQL	1889-1897
VSPIEFYKF	131-139
HEYGHHITL	2127-2135
SALLNSSSL	1402-1410
ITLKSFSIV	154-162
SDEQGTKDI	3262-3270
FEKLTKLLA	932-940

TYKLNKSVL	215-223
FSLLYSEVL	871-879
NYTESLKRL	2675-2683
NMNEFFSVL	2005-2013
NPNGIWNML	2641-2649
KYQAAWVTS	1747-1755
FSNIYKNYL	2477-2485
VDLKAITLV	2871-2879
AYNHSLTHS	1877-1885
SDVERNRLV	286-294
IVRRLGRNL	1566-1574
NYNNTNEIV	355-363
YEAYNHSLT	1875-1883
TYLNYKDEL	1345-1353
DYINKRKDI	1773-1781
TYLQALTVE	1788-1796
AMFNSALTF	2805-2813
TEWFMKNVS	140-148
SYFGEFQSI	3060-3068
LNLRYKKAV	3036-3044
GSFNFYNQY	119-127
IIVEQYKVL	1516-1524
LYEAYNHSL	1874-1882
SKFKKLPQL	1806-1814
EETLKNSLA	441-449
SVPEFLKKF	1544-1552
KNMNEFFSV	2004-2012
FYNQYIEAV	123-131
YYINRNNSS	2536-2544
KAIEDADKL	483-491
TVFDTTQQL	716-724
EWLTLRQTI	460-468
SERANVASF	1424-1432
ISTDFLKFV	2116-2124

			EEKVKTESI	496-504		
			SVYEHEQTL	655-663		
			EEKRLADEV	207-215		
			EESKTLKRL	517-525		
			TENFSDSFF	530-538		
			VSSSCFNNI	19-27		
			HEYYSAAHT	313-321		
			GEVVVTFSV	104-112		
			EYYSAAHTK	314-322	YEEEKALLV	389-397
			IEKLTAEKL	346-354	FEKQKMNLL	684-692
			VEEALTRQM	292-300	KYQSSEATM	273-281
3	Mbov_0049[a,b]	AFM51424_1	SYESQLVQV	377-385	KAYIYCELL	583-591
			FPLVSSSCF	16-24	NEGFNHQII	544-552
			IEKLQEFIL	639-647	YESQLVQVV	378-386
			LMHEYYSAA	311-319	LEELKLEEI	709-717
			FDDEYTDLV	284-292	YEHEQTLEL	657-665
			RESYESQLV	375-383		
			FEYEDLEEL	704-712		
			EEALTRQMY	293-301		
			NNRVFWAEV	225-233		
			NHNNSNRPI	165-173		
			QDAYFKGLM	304-312		
			VKVVNLGEI	48-56		

			NENIFKTAV	479-487		
			SVWRYKLEL	141-149		
			AGRYYYAGW	425-433		
			NWEYLAKYI	600-608		
			KEALFSPIV	119-127		
			IYQATSTDA	195-203		
			LELGSKVIV	147-155		
			SEYSFDGSV	91-99		
			ASHTSYANF	764-772		
			NEHFAKLAF	556-564		
			NPTTSPYQF	528-536		
			SAVGLATPL	15-23		
			RPAVSEYSF	87-95		
			GYESGESKV	61-69	TSYANFASI	767-775
			FEVVKGQKW	223-231	LESAQGILI	109-117
4	Mbov_0111[a,b]	AFM51486_1	VKYANSLPL	894-902	NSAINWEYL	596-604
			WIPDLYMAL	734-742	PYQFLTSYI	533-541
			TAVAFRTLI	587-595	TSYANFASI	767-775
			SLIAPNTAI	618-626		
			SIATFKNPI	187-195		
			LAFAPQSLL	496-504		
			YEVVAKDFY	241-249		
			YYYAGWNPS	428-436		
			VKYANSLPL	894-902		
			YYSWLRTTG	249-257		
			LYMALDPRL	738-746		
			KIGLYWYGF	409-417		
			NDFLRGKLA	489-497		
			YYGRVPEPA	915-923		
			SEYSFDGSV	91-99		

			KYNTSSSAP	368-376		
			NEYNVHYSL	294-302		
			KEFTAKLQV	327-335		
			EDLTKDHFI	123-131	MEKFKKKVI	1-9
			YHNTTNGYI	380-388	VHYSLFTYV	298-306
			KSIDNANAL	268-276	GEAIIAVKI	456-464
5	Mbov_0154[a,b]	AFM51527_1	DYNHTVKSI	262-270	TEALNQIEI	341-349
			RYASEFAYI	66-74	SEFAYISFF	69-77
			EYTVSYHKI	411-419	LENGYTESI	468-476
			VHYSLFTYV	298-306	ISKEFTAKL	325-333
			IGTLKLTGL	477-485		
			KANSNETEA	34-42		
			FTYVPNAQF	303-311		
			YEQLKNKVW	449-457		
			YDALNSILI	331-339		
			FDLDHYNLV	218-226		
			SYINQIVPV	362-370		
			EEIWKNFAL	53-61		
			KYAFENKYI	227-235		
			IEAFAHLGV	429-437		
			KELQGYAGI	294-302		
			LEEIWKNFA	52-60	SELKKLQTI	179-187
			SILISFNDL	336-344	KAIEAFAHL	427-435
6	Mbov_0217[a,b]	AFM51586_1	KEINNMLLV	164-172	GETDQKDFI	144-152
			NDVFTRKEI	249-257	FENKYIQLW	230-238
			NETDNKRRV	314-322		
			ISKKLLASL	4-12		
			SIVSYINQI	359-367		
			EYYFVEKSL	113-121		
			LYEQLKNKV	448-456		
			KLQTIANNI	183-191		
			VYDALNSIL	330-338		
			FEGTIYKEY	400-408		

7	Mbov_0274[a]	AFM51642_1	SDYGITKSI	123-131	NEAALPNLI	95-103
			IPLAVAAPM	11-19	EEFRILNFL	512-520
			SEIKEDDLI	571-579	FAQSNSWPL	35-43
			REFELIKKV	79-87	ASVVNDESL	482-490
			AVVYNLEVL	164-172	DETLTGEVI	261-269
			ISVDYANDL	269-277	TEGGSSLLV	425-433
			KPVNVKYNI	303-311	KEDDLIKSI	574-582
			GYIMPLASV	476-484	NEVFKGLTV	222-230
			FETEADKLK	493-501	SAIGFAASV	367-375
			NSTMYSSRL	378-386	LNFLRSAYV	517-525
			VEENWKTFA	464-472		
8	Mbov_0290[b]	AFM51658_1	TYLLRETNI	206-214		
			KYKEQETQL	249-257		
			STFSFDGIV	408-416		
			KTLEKFAGF	360-368	EEHYYGELI	171-179
			AELEQLKKL	230-238	QETQLRKSV	253-261
			LDSLFVLSI	115-123	QELWNTYLL	201-209
			KSFIWQNSI	383-391	TEWENIVQV	451-459
			FSFVLLPIL	4-12	GELIIGNDI	289-297
			STDLFDAEL	224-232	SENDELKKI	41-49
			LEKIIVKDM	425-433		
			GEEHYYGEL	170-178		
			FSFVLLPIL	4-12		

			FVFDNFYGL	249-257			
			EENYIFDSL	424-432			
			FESRRLLKV	370-378			
			AYSYSFNTL	486-494			
			KWFLLTSNL	517-525			
			YYGVDINTI	207-215			
			GIMHNLTPL	141-149	RQFNYYTPL	308-316	
			IPLFVFNLL	183-191	FESWLDPSI	268-276	
			KAYSYSFNT	485-493	IIPLFVFNL	182-190	
			AALYKLIGL	469-477	LSISFLPLL	8-16	
			IYHANGASL	106-114	SELTNKFKI	224-232	
9	Mbov_0296[b]	AFM51664_1	YSYSFNTLT	487-495	VSVENGFEV	592-600	
			LSFSAANAL	525-533	LEYTDNNYI	316	324
			KDILNRDPL	278-286	EESVYRAEI	499	507
			TYYFNNDRI	61-69	KPQSIKFGL	159	167
			SFNTLTRTL	490-498	PYVSVKQAL	70	78
			KEPIIPLFV	179-187	NDDALAKLV	613	621
			KNYKWFLLT	514-522	YEHIGKEGI	51	59
			SNDKHYVSV	586-594			
			FSAANALVA	527-535			
			KYNWKIHAV	169-177			
			YNKGYFKAL	288-296			
			IHAVNKEPI	174-182			

			KFHTATHNI	133-141		
			FYYMDFKSI	245-253		
			NENNFQSLW	576-584		
			ISKNPFGYL	229-237		
			IGLVISSNL	8-16		
			AYYTPRYNV	449-457		
			LNFNFNELI	221-229		
			YLPSNLSQL	236-244		
			SQLFYYMDF	242-250		
			SILIWFNRW	315-323		
			AYTISRSTS	124-132	YELLLATNI	361-369
			YDRSFSILI	310-318	QEELTSNII	421-429
10	Mbov_0350[a,b]	AFM51716_1	TDNRFWEHI	164-172	NELISKNPF	226-234
			QEDEITKDF	331-339	KHNTTSNAI	666-674
			YIYDRSFSI	308-316	VQPINPTNI	80-88
			KNRGWYGDL	173-181		
			IYDRSFSIL	309-317		
			VNENNFQSL	575-583		
			IEKEDDALV	631-639		
			LTYKFQANK	554-562		
			FEKNSKHKF	384-392		
			YKFQANKSI	556-564		
			KNGEFEILL	272-280		
			YQYIYDRSF	306-314		
			GYLPSNLSQ	235-243		
			IESWDTSNI	357-365		
			SSLINKASF	93-101		
11	Mbov_0364[a,b]	AFM51726_1	VEKMQSMFY	415-423	DESLKKTFI	221-229
			SNFCTNSGL	444-452	TEKEIINLV	112-120
			SMFYGASEF	420-428		
			VTFFNAKKF	395-403		

			SDTEAKTPI	40-48		
			YELDHNDRI	393-401		
			LSYATYESI	187-195		
			EEEVKGLEV	154-162		
			FNLRNISNI	199-207		
			LEKKFKLLL	6-14		
			IHSKTKTYI	69-77		
			TTLDQFSTL	592-600		
			SFVLSCTNV	24-32		
			YLPSNLSQL	178-186	WEKYQQPFI	563-571
			SYFNTKNSG	443-451	FEDAYAQYL	404-412
			DENWFRDVW	537-545	RPQTNGIVL	663-671
12	Mbov_0374[b]	AFM51736_1	VWLRYDEKL	544-552	ANIDFFTYI	245-253
			SSFNVIGIL	623-631	RYESNNTYL	420-428
			YHYDKFKNV	323-331	YELLLATNM	302-310
			SDTEAKTPI	40-48	IDGLKSKLI	689-697
			KNFLKTRNI	84-92		
			KFLGTAWVI	282-290		
			ATNMHVFNL	307-315		
			GHYLNHQHL	141-149		
			EYWSYAKYL	109-117		
			GIFNFPKFL	276-284		
			FNFPKFLGT	278-286		
			GYLPSNLSQ	177-185		
			SETLKNNFS	435-443		

			IYNGVSSNV	493-501		
			LAYNEYGQM	482-490		
			FALKFLTKL	94-102		
			NDNKDNKLI	57-65		
			LYYGASGSL	474-482		
			FDSYWGRVL	451-459		
			SSISSLVLL	14-22		
			FIATNLHVL	134-142		
			QYNVNFSSL	466-474		
			SSNVQFGDL	498-506	TEAISSPKI	228-236
			SNVQFGDLL	499-507	SENNVGEKI	435-443
13	Mbov_0468[a,b]	AFM51824_1	KYQDKLNEV	251-259	NEYGQMIGI	485-493
			RVLAAWYGF	457-465	KYLQTVDYV	344-352
			VPMMVDFGI	289-297	AWYGFQYNV	461-469
			SSISSLVLL	14-22		
			FALKFLTKL	94-102		
			YGFQYNVNF	463-471		
			IFDSYWGRV	450-458		
			AYNEYGQMI	483-491		
			TEGFSENNV	431-439		
			QNQSMKFDF	400-408		
			LLIPMLSSI	8-16		
			NQTSLFKII	72-80		

			VDYKLKKII	116-124		
			FQLKYYPFL	240-248		
			EFLRSISTI	75-83		
			PYAFSAGNI	446-454		
			IGYKTFGGA	468-476		
			NIFRFAESL	206-214		
			ASAISYAIL	476-484		
			FLFDYYYGI	247-255		
			IELIQSKYP	426-434		
			DESALKEFL	33-41		
			KDNNVGKVI	460-468	FEEDSASHI	362-370
14	Mbov_0471[a,b]	AFM51827_1	KNIVFNLTL	383-391	SNYKFFTEI	126-134
			FEFDLKKYN	161-169	HYLSTKKII	282-290
			AYVGIKEFL	69-77	EEKLKISFL	142-150
			SLNSNSSTI	586-594		
			KYERGEEKL	137-145		
			YMHNDVAYV	63-71		
			SLNSNSSTI	586-594		
			NESNIQTYF	192-200		
			EYQNVYTPD	343-351		
			ISSAFILPF	11-19		
			YPFLFDYYY	245-253		
			FDYYYGIKL	249-257		
			IAIILSTVV	617-625		

			NDLDFYQYI	362-370		
			LYNTYSNAI	748-756		
			DHLSTSQTI	394-402		
			SAIKHTIGL	277-285		
			YEQYLTAPY	514-522		
			NSNINAENI	45-53		
			IAKSFKISL	97-105		
			WEYTKEIGI	229-237		
			YSNNFDGNL	765-773		
			IENDAVISF	77-85		
			SCFENAYNI	26-34		
			YIYDHSFSI	369-377		
			TFYGGYPSV	610-618		
			KHHPIKTTF	603-611		
			FLPSNLSQL	296-304		
			FDGEHGMPL	690-698	FEYDKSKYI	60-68
15	Mbov_0505[a,b]	AFM51861_1	EYKHAVNNI	190-198	NQHSNMYTL	702-710
			KYWEYTKEI	227-235	NEAEKNSAI	271-279
			KTFDKSLYF	437-445	YELLLATNM	422-430
			KLYNTYSNA	747-755		
			DYKSASKVL	309-317		
			NDAVISFNI	79-87		
			YIQKFLPNF	262-270		
			AENIKIDDI	50-58		
			FYNNTLSAN	251-259		
			ATNMHVFNL	427-435		
			IAKNPFGFL	289-297		
			NTYSNAISL	750-758		
			FKNNNHPPI	89-97		
			NFYDSITNI	465-473		
			FDGNLRNDI	769-777		
			TTINQHSNM	699-707		
			AVISFNIAF	81-89		

16	Mbov_0515[a,b]	AFM51869_1	FEREVAKAP	262-270		
			SGLILRDPI	159-167		
			SNSQFKTNL	826-834		
			EEYIDFAVV	532-540		
			VEEYIDFAV	531-539		
			FEDKNEEQI	327-335		
			SYLESQIKI	337-345		
			GELDVSLLF	124-132		
			SYREALKTM	814-822	KYTNVISAI	97-105
			KAPSHVDTL	268-276	YEKDIKNHI	580-588
			VTFNFEKDF	305-313	TEKKLPTII	111-119
			VSFKFSNGG	62-70	AENNKDKLI	565-573
			DYENVEEYI	527-535	SYLDNYNSI	594-602
			TVNSKYTNV	93-101	FEKDFRKEL	309-317
			NYNSIDVPL	598-606	NEEQIKSYL	331-339
			FFFGTNSHV	446-454	EENTQNLSV	84-92
			KDYYLERYI	635-643		
			SYIHSSQKA	518-526		
			SYQIGYRTF	691-699		
			KYVNYGLHL	732-740		
			FSFSKPSGI	493-501		
			LAPRFYAPN	740-748		
			DGFKKYLGM	174-182		
			YAENNKDKL	564-572		

			LEKEGFKSL	269-277			
			QYNKVTSRL	333-341			
			IEKPIEKKI	81-89			
			SGYGKLKNL	423-431			
			KYYQYSDAL	704-712			
			GYGKLKNLI	424-432			
			AQATNTSSL	479-487			
			DYSRTIDSI	59-67			
			KYYQYSDAL	704-712			
			EEGKGIGLF	198-206			
			SLYTMGNSL	499-507			
			VTVSKLLNI	172-180			
17	Mbov_0516[a,b]	AFM51870_1	VEEDNGLTF	739-747	WYLRSSDSI	356-364	
			GYSTNSMKI	677-685	FELTRQNPV	395-403	
			ANISGYQNV	164-172			
			SQYPPNTPI	533-541			
			KYIWIIANL	280-288			
			DYLKKGLAI	302-310			
			LYTMGNSLL	500-508			
			TPIATRISF	539-547			
			SEKVKIFGV	132-140			
			LDKAEAVLI	223-231			
			SLYTMGNSL	499-507			
			ITSYRIFNM	442-450			
			EEKITPPKV	90-98			

			FNLKKFFSL	693-701		
			SYETYDSQL	45-53		
			SEGTNSYPL	589-597		
			SDFNFAQSL	35-43		
			VPIATRITF	532-540		
			VSSEYARNV	524-532		
			GYKKTKELI	417-425		
			NERIFQGSF	572-580		
			GEETKSINI	673-681		
			LENKKIKEL	483-491	VETGTGSFI	731-739
			KELLDSKNV	240-248	TEELRKKTV	178-186
			TDYDRTKGI	150-158	SEYARNVPI	526-534
18	Mbov_0517[a,b]	AFM51871_1	KYIWLIANL	271-279	WAFKNVAWI	509-517
			YETYDSQLA	46-54	FDGTFFKVI	199-207
			SEYQTKQFG	258-266	YYARNNERI	567-575
			VIVNKLISV	167-175	TEKLNSDGI	684-692
			KFFSLNDAL	697-705		
			NNLFRYKEL	234-242		
			SDFNFAQSL	35-43		
			SEGTNSYPL	589-597		
			FNPWAFKNV	506-514		
			KYIWLIANL	271-279		
			LYTLGNSLL	493-501		
			TEFSSYKIG	371-379		
			FSLLSGGVL	14-22		

19	Mbov_0518[b]	AFM51872_1	YGYDYKGLL	780-788	YEIGYRSFI	693-701
			SYLKDYDQI	595-603	EEFMDFAVI	528-536
			KYYKNVTTQ	665-673	KYANSISVI	93-101
			SSFDSYINA	234-242	STIPFLSTL	11-19
			TESGTMWLM	420-428	FYFGTNSHV	442-450
			LESKPADFL	508-516	MYMAMLKNI	192-200
			VEEFMDFAV	527-535	TYQVSFTVL	303-311
			IEIDFEKVL	536-544	TSFIFDSNF	57-65
			LMPRSYVPM	735-743		
			YPLSVGDYF	631-639		
			DNPMYMAML	189-197		
			SYVPMGGAS	739-747		
			KYFNYGLHL	727-735		
			LEYHKLSHL	174-182		
			LMPRSYVPM	735-743		
			VSFTVLNTF	306-314		
			SDNKSIKLL	31-39		
			QEQINSFTF	333-341		
			GVISNNQFL	551-559		
			TMWLMDFML	424-432		
			NDKDFEQLI	40-48		
			KYKNTEELI	566-574		
			SGLLEYHKL	171-179		
			KYFNYGLHL	727-735		
			SLYILGYPL	625-633		
			SNFGPSKPL	63-71		
			LDDRFTKFL	481-489		
			KYQSNGKTY	817-825		
			KVLQNKESL	542-550		
			NTEELIKLI	569-577		
			GDYFLENYI	636-644		

			TFFVFLNNI	199-207		
			KNRVIFATL	8-16		
			FDSIVQRQI	261-269		
			NELDQKAVW	680-688		
			LENKRIKEL	479-487		
			SNVNPYQNI	156-164		
			SDIDLSRVI	35-43		
			IMFSNGDTT	663-671		
			DAVMNNPWI	219-227	LALRNTEWI	505-513
20	Mbov_0519[a,b]	AFM51873_1	FAGTFFVFL	196-204	VENEKPVVI	72-80
			IEVTPDRVI	138-146	LERLGYKVV	719-727
			LDQKAVWNL	682-690	FEHFATDSM	647-655
			QFNTVTSRI	320-328		
			SGYDKTLKL	412-420		
			IIVSKVLNV	164-172		
			NEGNNSYPM	584-592		
			VSRDFRPNT	520-528		
			LYTLGNSLL	489-497		
			FAGTFFVFL	196-204		
			RESNLTKAI	275-283		
			KEIDFSKIL	323-331		
			LYEKVHNEL	157-165		
			QENKSNALF	75-83		
			FSLTYYPIF	88-96		
			DSNANAETI	310-318		
21	Mbov_0536[a,b]	AFM51890_1	ANYQYNELF	348-356		
			FDSNKAIKI	226-234		
			NELSVYRFT	366-374		
			ISIPKKEPL	39-47		
			SNIESWYLI	217-225		
			KLLTTSTVV	3-11		
			LFVEAINTI	355-363		

22	Mbov_0570[a,b]	AFM51924_1	KEKLMNKSL	698-706	TEVKKGQII	594-602
			TEGITTKNL	436-444	FDTEAFKAI	106-114
			VVVPRFVHI	475-483	AELIDVHTI	338-346
			LGVGRYSLL	411-419	HIYMSFLHL	548-556
			VYLNNISML	711-719		
			IAPDFQPVL	488-496		
			NEKPFADAL	188-196		
			QAIGFSALI	13-21		
			IGFSALIPV	15-23		
			QNNYMSKYI	96-104		
			VYLNNISML	711-719		
			TSQDFEVKL	207-215		
			INNLQLAEL	332-340		
			FEKYNQVNL	53-61		
			FGIEDLFEL	744-752		
			KYINFDTEA	102-110		
			KPETVEYSL	85-93		
			YTLKFNKFI	272-280		
			AAYELTTKV	505-513		
			HAHVNLYTN	617-625		
			VIKIPVANL	523-531		
			LPSIDKTFF	444-452		
			YSLLFDKQF	416-424		

			ISSSTFENL	246-254		
			NYGSLASRL	111-119		
			RANSNTENL	156-164		
			FEKTDTSKI	644-652		
			FDKEIIGSI	711-719		
			STFENLEEL	249-257		
			TYNELKEAV	660-668		
			YAMINADAN	295-303		
			FENKGAKFD	195-203		
			VIYSQGPSL	566-574		
			SIIASSSQI	718-726		
			SYQTKHEYA	361-369		
			IYSQGPSLI	567-575		
			FMIPMLKSI	170-178	EELLNFANI	255-263
23	Mbov_0579[a,b]	AFM51933_1	KFLTSLEKI	592-600	KESLATKSL	342-350
			IYNKFKESL	337-345	DESEVQKLW	222-230
			KEAVTNKNV	665-673		
			SYKSLDSAT	453-461		
			NADANDFFI	299-307		
			NLPNNGTFM	163-171		
			TEKGVDQLF	414-422		
			VIYEEPAGF	674-682		
			LEDFDKEII	708-716		
			FESSQQDAV	233-241		
			AYLKAKNDQ	698-706		
			YIVHTYNEL	656-664		
			STFENLEEL	249-257		
			TAIPLLAAV	14-22		
			LPNNGTFMI	164-172		

24	Mbov_0580[a,b]	AFM51934_1	LTLPYYDNI	296-304	VYWEHYALL	315-323
			RSIKNTLIV	3-11	HSPTNLYNI	368-376
			FESNKASAF	160-168	WEHYALLRL	317-325
			IPGEKDYFL	79-87		
			LSIKNDKFI	141-149		
			LPYWIQSNI	345-353		
			KPNGKWTLL	357-365		
			GEKDYFLKI	81-89		
			KGFFKTFAL	337-345		
			AEINLEFKI	113-121		
			YQYLYSVEI	283-291		
			TSASAFLPV	13-21		
			SNLEHNYAL	236-244		
			WDKLKAKYF	175-183		
25	Mbov_0585[a,b]	AFM51939_1	PYLSTFLAI	144-152	DMISNYEEI	419-427
			YEYGYLLAL	227-235	ITFRNLTSL	114-122
			NYGTTSIQI	316-324	KEFLKTNPV	39-47
			NNYEYGYLL	225-233	SSLSEFNSL	167-175
			GYLLALPPI	230-238		
			INFLRNYDL	238-246		
			SSYKIYDII	375-383		
			ITFRNLTSL	114-122		
			DEIKDLDMI	413-421		
			FNYYSDKFI	183-191		
			EYDTRASNI	78-86		
			SLTITFRNL	111-119		
			KYLYFANDV	101-109		
			YYDTMKNYG	310-318		
			KKYDFKYFL	354-362		
			SYAQTFNYY	178-186		
			KNNYEYGYL	224-232		

26	Mbov_0658[a,b]	AFM52009_1	GYIGAAYEI	394-402	FEEDSATHI	361-369
			EEKLDIQFL	141-149	SNYKFFVEI	125-133
			YYSKSSDLI	304-312	FNVWTYNPL	411-419
			GYYSKSSDL	303-311	HYLSTRKIV	281-289
			FLLDYYYGL	246-254		
			YYILPTGDI	480-488		
			AAYEIMGFL	398-406		
			IETGAKNLY	519-527		
			KNIIFNVTL	382-390		
			KYKSQITHT	268-276		
			NYFVLTAPV	438-446		
			SAGNIFPQL	449-457		
			NESNVQTYF	191-199		
			YYYGLKSES	250-258		
			LDDLHSAYI	292-300		
			AYIGIQEFL	68-76		
			QSKYFSFLL	240-248		
			MTYFLVKRF	634-642		
			RDNNLGKII	459-467		
			NDLYLPQIL	176-184		
			KPFNVWTYN	409-417		
			FQSKYFSFL	239-247		
			ITSCTIAPL	11-19		

			KETPFGGSV	212-220		
			KYMSNPSYT	167-175		
			VNQIYYNKF	3-11		
			KAYFKTQAL	107-115		
			PPYAMKFKI	226-234		
			WIYTYAGLV	18-26		
			TSINKYANS	339-347	FEMKKFKWI	11-19
			SELNEAHNL	272-280	NEAHNLQYV	275-283
			QYVKSSSSK	388-396	YVFDYFNEL	282-290
27	Mbov_0674[a,b]	AFM52024_1	HSPVGYTVL	412-420	KWIYTYAGL	17-25
			IYTYAGLVI	19-27	KYKSNSGYI	397-405
			LFFAGDTNI	297-305	KYANSYDKI	343-351
			GDGEFAGFL	178-186	INIGFWNVL	90-98
			YDFVNNGFL	368-376		
			FLYKNINSL	375-383		
			SSLSFTTSC	30-38		
			VIFNQKYDL	118-126		
			FEGAGSSEL	266-274		
			KIFKLYDFV	363-371		

28	Mbov_0675[a,b]	AFM52025_1	SYIKFGQLI	638-646	NEQVFTPYI	246-254
			VEKDKDYYI	593-601	KEAINAKDI	62-70
			VESDIKELV	51-59	ESYIKFGQL	637-645
			LESGIKVGI	258-266	YYIVTNDFI	599-607
			AWVKTRTPI	84-92	VEELKSKGV	299-307
			CYTKYLSEL	366-374	KETKDDKFI	570-578
			KGVNFIIAL	305-313		
			YIVTNDFIL	600-608		
			ADAAAYDFI	471-479		
			VHVTFLDPI	284-292		
			SDSDKGLTI	173-181		
			AYAQYSHNV	555-563		
			IEFKHTEEV	435-443		
			NFIIALTHL	308-316		
			SAFAAIPMV	13-21		
			RTIEYTELL	393-401		
			VVFNLPIEF	429-437		
			KFILGLGAI	4-12		
29	Mbov_0739[a,b]	AFM52087_1	GEYQLLKKV	95-103	EEEDNWYTI	484-492
			LNMLRSAQL	544-552	SAISFAASV	384-392
			NEYGEYQLL	92-100	GAYQNKITM	393-401
			EEEKVVFRL	28-36	QIFRFQSAI	378-386
			FELDKDKNV	310-318	IENENVEGI	202-210
			TETIYNIPF	165-173		
			KEWGSWQIF	372-380		
			TELDRNKEF	591-599		
			WYTIARTSG	489-497		
			IFHEGGSSI	445-453		
			KSIADLHSI	150-158		
			GYIMPLASV	497-505		
			IRFEFADNF	68-76		
			LPAAAGLSV	13-21		
			SEHDVLMLP	425-433		
			SAISFAASV	384-392		

30	Mbov_0743[a,b]	AFM52091_1	YENEFSNYY	303-311		
			TEAIQANSY	422-430		
			ISYDHIKEL	606-614		
			SLMLNTNEM	88-96		
			SKFVKYTYI	578-586		
			VSLTLTAAV	888-896		
			VNISKVNLL	668-676		
			VIPMLSMSL	23-31		
			LNFNTYKSI	828-836		
			IDLKVNKFL	491-499		
			INYQNGDHI	691-699		
			TNVLNFNYV	616-624		
			TNVLNFNYV	616-624	YVLNNTTPV	447-455
			FKVSNNDLI	483-491	NELRYSWEI	791-799
			NEFSNYYNV	305-313	LEKPNSKFV	573-581
			GYEYIGKNI	286-294	FETYDFDKI	175-183
			SYVLNNTTP	446-454	KYHSSIKII	772-780
			TYKSINQLL	832-840	KYYISYDHI	603-611
			VNFLPYNSS	270-278	NDQTYANPI	631-639
			VAIRGYKTV	195-203	KYTFSKLYI	535-543
			KHITKNGSL	185-193	FDVNVSSFI	507-515
			VPFAESGTF	557-565	YEYIGKNIL	287-295
			VNKKFMNWL	6-14		
			VIVGSKAPL	378-386		
			FSLRDDYIL	57-65		
			KYYISYDHI	603-611		
			IEVQSNSKV	212-220		
			SSFIDDSFI	512-520		
			ANDKYYKNI	204-212		
			TDGTTGAWI	248-256		
			NQYQYNTGL	67-75		
			YYNVANYGV	310-318		
			NYYNVANYG	309-317		
			LPYNSSAII	273-281		

			GEVTLKFEI	109-117
			GEVTLKFEI	195-203
			IEAAIKEAI	69-77
31	Mbov_0798[a,b]	AFM52146_1	IEAAIKEAI	155-163
			FEGEVTLKF	107-115
			FEGEVTLKF	193-201
			SKGENYLPI	253-261

a: related to *M. bovis* HB0801-P1, b: related to P150 and a,b: related to both (*M. bovis* HB0801-P1, and P150).

Table S6 Identification the affinity of T cell epitopes to MHC class II (15-mers) of recognized *M. bovis* secreted proteins by using IEDB-AR

No	Proteins		Intermediate affinity binding		High affinity binding	
	Mnemonic	Accession No	Sequence	Position	Sequence	Position
1	Mbov_0016[a,b]	AFM51394_1	FYLFLGAAPVLSVPL	6-20		
			YLFLGAAPVLSVPLV	7-21		
			KKNKFYLFLGAAPVL	2-16		
			MKKNKFYLFLGAAPV	1-15		
			QAAYIAGRALADYFS	204-218		
			TKQAAYIAGRALADY	202-216		
			DTKQAAYIAGRALAD	201-215	KFYLFLGAAPVLSVP	5-19
			FDTKQAAYIAGRALA	200-214	NKFYLFLGAAPVLSV	4-18
			IKATASYPVAGSLSS	292-306	KNKFYLFLGAAPVLS	3-17
			KQAAYIAGRALADYF	203-217		
			KATASYPVAGSLSSD	293-307		
			SVIKATASYPVAGSL	290-304		
			LKTSFTSGEPVAVAA	273-287		
			KTSFTSGEPVAVAAI	274-288		
			TSFTSGEPVAVAAIN	275-289		

2	Mbov_0038[a,b]	AFM51416_1	NGKTFSQASIRMAKS	3284-3298	TFSQASIRMAKSEKP	3287-3301
			KTFSQASIRMAKSEK	3286-3300	FSQASIRMAKSEKPY	3288-3302
			EKFEVDLKAITLVDS	2867-2881	GKTFSQASIRMAKSE	3285-3299
			KFEVDLKAITLVDSL	2868-2882		
			LFSDYTYNFAEVINR	2941-2955		
			FSDYTYNFAEVINRD	2942-2956		
			SKYTPTFITSSSNPN	2629-2643		
			ELFSDYTYNFAEVIN	2940-2954		
			SDYTYNFAEVINRDN	2943-2957		
			NELFSDYTYNFAEVI	2939-2953		
			PIFVSALTPNATPNI	2148-2162		
			TDLRNYAGARKAIED	473-487		
			ETDLRNYAGARKAIE	472-486		
			YTPTFITSSSNPNGI	2631-2645		
			KYTPTFITSSSNPNG	2630-2644		
			ITNVVLIKALESHKI	1137-1151		
			DLRNYAGARKAIEDA	474-488		
			DPIFVSALTPNATPN	2147-2161		
			IKKLKAEKATLLNKT	1205-1219		
			IFVSALTPNATPNIN	2149-2163		
			METDLRNYAGARKAI	471-485		
			SDPIFVSALTPNATP	2146-2160		
			ENGKTFSQASIRMAK	3283-3297		
			VRYLKNQKVYALRRP	260-274		
			GSDPIFVSALTPNAT	2145-2159		
			SIKKLKAEKATLLNK	1204-1218		
			LGYKKMVAPSVIKEE	2013-2027		
			TNVVLIKALESHKID	1138-1152		
			VLGYKKMVAPSVIKE	2012-2026		
			ISKYQAAWVTSPFID	1745-1759		
			YMETDLRNYAGARKA	470-484		
			TNVRYLKNQKVYALR	258-272		
			NVRYLKNQKVYALRR	259-273		
			DSIKKLKAEKATLLN	1203-1217		

			TPTFITSSSNPNGIW	2632-2646
			RKKWEIGAEDTIKTY	2848-2862
			DRKKWEIGAEDTIKT	2847-2861
			FVSALTPNATPNINN	2150-2164
			EDSIKKLKAEKATLL	1202-1216
			DISKYQAAWVTSPFI	1744-1758
			PTFITSSSNPNGIWN	2633-2647
			LRNYAGARKAIEDAD	475-489
			SKYQAAWVTSPFIDV	1746-1760
			GAQQKLVDALTEILS	620-634
			VAELQKQATGLVTKI	1615-1629
			KVEDSIKKLKAEKAT	1200-1214
			DISKYQAAWVTSPFI	1744-1758
			IKNKIDLKSLPALHL	1266-1280
			GLGEYFNTLIAPAIR	1856-1870
			DYTYNFAEVINRDNL	2944-2958
			RDISKYQAAWVTSPF	1743-1757
			LGEYFNTLIAPAIRD	1857-1871
			YTYNFAEVINRDNLQ	2945-2959
			NKVEAIKKLVAPYYE	848-862
			YHKYQSSEATMRLFD	271-285
			HKYQSSEATMRLFDD	272-286
			YYHKYQSSEATMRLF	270-284
			KYQSSEATMRLFDDE	273-287
			ESYESQLVQVVDAYE	376-390
			RESYESQLVQVVDAY	375-389
3	Mbov_0049[a,b]	AFM51424_1	LYYHKYQSSEATMRL	269-283
			RLYYHKYQSSEATMR	268-282
			SYESQLVQVVDAYEE	377-391
			LRESYESQLVQVVDA	374-388
			YQSSEATMRLFDDEY	274-288
			YYHKYQSSEATMRLF	270-284
			ILKSLQASQSVYEHE	646-660

			NKLFLYLGTSAVGLA	6-20
			RNKLFLYLGTSAVGL	5-19
			KLFLYLGTSAVGLAT	7-21
			KRNKLFLYLGTSAVG	4-18
			DAREEWVSAIASHTS	754-768
			AREEWVSAIASHTSY	755-769
4	Mbov_0111[a,b]	AFM51486_1	TKRNKLFLYLGTSAV	3-17
			LFLYLGTSAVGLATP	8-22
			FLYLGTSAVGLATPL	9-23
			AGRYYYAGWNPSNRE	425-439
			LGLSSLRVILVLINS	789-803
			TAGRYYYAGWNPSNR	424-438
			GRYYYAGWNPSNREE	426-440
			SSLKYNTSSSAPNDS	365-379
			SLKYNTSSSAPNDSY	366-380
5	Mbov_0154[a,b]	AFM51527_1	PSSLKYNTSSSAPND	364-378
			IPSSLKYNTSSSAPN	363-377
			RKEINNMLLVKAYFD	163-177
			SKNLLEKAIEAFAHL	421-435
6	Mbov_0217[a,b]	AFM51586_1	KEINNMLLVKAYFDI	164-178
			KNLLEKAIEAFAHLG	422-436
			NLLEKAIEAFAHLGV	423-437

7	Mbov_0274[a]	AFM51642_1	RSAYVSIKSIRDFEK	521-535	FLRSAYVSIKSIRDF	519-533
			AKYSGYIMPLASVVN	472-486	LRSAYVSIKSIRDFE	520-534
			FAKYSGYIMPLASVV	471-485	NFLRSAYVSIKSIRD	518-532
			KYSGYIMPLASVVND	473-487		
			LNFLRSAYVSIKSIR	517-531		
			SAYVSIKSIRDFEKD	522-536		
			KKVVFQFAQSNSWPL	29-43		
			VDEKATIVQKATEAG	186-200		
			YSGYIMPLASVVNDE	474-488		
			KVVFQFAQSNSWPLP	30-44		
			VVFQFAQSNSWPLPK	31-45		
			SGYIMPLASVVNDES	475-489		
			NVDEKATIVQKATEA	185-199		
			IIPLAVAAPMIAASC	10-24		
			GIIPLAVAAPMIAAS	9-23		
			IPLAVAAPMIAASCQ	11-25		
			FGIIPLAVAAPMIAA	8-22		
			FQFAQSNSWPLPKML	33-47		
			VFQFAQSNSWPLPKM	32-46		
			GNVDEKATIVQKATE	184-198		
			DEKATIVQKATEAGR	187-201		
			SKDSSMNKAVAKFVK	436-450		

			ISKIAITASKAKELA	268-282
			KALVATKEAKAKLTK	136-150
			DKISKIAITASKAKE	266-280
			LDKISKIAITASKAK	265-279
			KISKIAITASKAKEL	267-281
			ILDKISKIAITASKA	264-278
			DKALVATKEAKAKLT	135-149
			FGKSAKVVQDAVDAH	78-92
8	Mbov_0290[b]	AFM51658_1	KSAKVVQDAVDAHHK	80-94
			FDKALVATKEAKAKL	134-148
			VLEKSIKAAESIKKG	156-170
			VFGKSAKVVQDAVDA	77-91
			DFDKALVATKEAKAK	133-147
			GKSAKVVQDAVDAHH	79-93
			KDMEAKMELIKKLKP	431-445
			LKKLFKDAKANKNKK	235-249
			DMEAKMELIKKLKPE	432-446

			LTSNLSFSAANALVA	521-535
			TSNLSFSAANALVAY	522-536
			SNLSFSAANALVAYT	523-537
			AGQLRTKLAALRERA	350-364
			NLSFSAANALVAYTA	524-538
			LSFSAANALVAYTAA	525-539
			LLTSNLSFSAANALV	520-534
			KNYQIYHANGASLKF	102-116
			NKNYQIYHANGASLK	101-115
			NAGQLRTKLAALRER	349-363
			LSFSAANALVAYTAA	525-539
			NYQIYHANGASLKFN	103-117
			FLLTSNLSFSAANAL	519-533
			YQIYHANGASLKFNW	104-118
			LELMKYALDVIQRRS	432-446
9	Mbov_0296[b]	AFM51664_1	SFSAANALVAYTAAD	526-540
			SLELMKYALDVIQRR	431-445
			LNAGQLRTKLAALRE	348-362
			FSAANALVAYTAADK	527-541
			GQLRTKLAALRERAI	351-365
			NLSFSAANALVAYTA	524-538
			ELMKYALDVIQRRSM	433-447
			IPYVSVKQALSALEG	69-83
			SAANALVAYTAADKS	528-542
			DNKSAVITLDSFSAA	400-414
			PYVSVKQALSALEGV	70-84
			QLRTKLAALRERAIY	352-366
			VSVKQALSALEGVVN	72-86
			YVSVKQALSALEGVV	71-85
			LRTKLAALRERAIYH	353-367
			RIPYVSVKQALSALE	68-82
			KTQFYAGYPATQLGM	536-550
10	Mbov_0350[a,b]	AFM51716_1	LKTQFYAGYPATQLG	535-549
			PLKTQFYAGYPATQL	534-548

11	Mbov_0364[a,b]	AFM51726_1	IALATAIAVPVTLQQ	16-30
			TIALATAIAVPVTLQ	15-29
			GTIALATAIAVPVTL	14-28
			IGTIALATAIAVPVT	13-27
			ALATAIAVPVTLQQK	17-31
			MKKKLTIALSSVIGT	1-15
			LATAIAVPVTLQQKN	18-32
			VIGTIALATAIAVPV	12-26
12	Mbov_0374[b]	AFM51736_1	IKKTKLKDVLPKLAS	461-475
			EGASGSMAIDSSFNV	613-627
			YQKTQFFNPSIPIGH	128-142
			GASGSMAIDSSFNVI	614-628
			QKTQFFNPSIPIGHY	129-143
			KIKKTKLKDVLPKLA	460-474
			KTQFFNPSIPIGHYL	130-144
			EAKTPIKRADKHFLA	43-57
13	Mbov_0468[a,b]	AFM51824_1	FDFSKFDPSKPFPIL	406-420
			DFSKFDPSKPFPILP	407-421
			KFDFSKFDPSKPFPI	405-419
			FSKFDPSKPFPILPH	408-422
			SKFDPSKPFPILPHN	409-423
			FSSLYYGASGSLAYN	471-485
			NFSSLYYGASGSLAY	470-484
			MMSKQKLLLIPMLSS	1-15
			SSLYYGASGSLAYNE	472-486
			SLYYGASGSLAYNEY	473-487
			SKYLQTVDYVSAFKT	343-357
			LYYGASGSLAYNEYG	474-488

14	Mbov_0471[a,b]	AFM51827_1	IGYKTFGGASAISYA	468-82
			VIGYKTFGGASAISY	467-481
			GYKTFGGASAISYAI	469-483
			KVIGYKTFGGASAIS	466-480
			GKVIGYKTFGGASAI	465-479
			DFNYYVLTSPYAFSA	437-451
			FNYYVLTSPYAFSAG	438-452
			YDFNYYVLTSPYAFS	436-450
			KYDFNYYVLTSPYAF	435-449
			NYYVLTSPYAFSAGN	439-453
			FDESALKEFLIIPLA	32-46
			YKTFGGASAISYAIL	470-484
			YVLTSPYAFSAGNIF	441-455
			LTSPYAFSAGNIFPQ	443-457
15	Mbov_0505[a,b]	AFM51861_1	AKSFKISLPKPSNSK	98-112
			IAKSFKISLPKPSNS	97-111
			KSFKISLPKPSNSKD	99-113
			PIAKSFKISLPKPSN	96-110
			PPIAKSFKISLPKPS	95-109
			PVNFRYGGADSNKEL	210-224
			VNFRYGGADSNKELL	211-225
			LLRLRIKKANLAQIL	565-579
			NPVNFRYGGADSNKE	209-223
			VLLRLRIKKANLAQI	564-578
			SFKISLPKPSNSKDT	100-114
			FVLLRLRIKKANLAQ	563-577
			GNPVNFRYGGADSNK	208-222
			FKISLPKPSNSKDTE	101-115
			SGNPVNFRYGGADSN	207-221
			DFVLLRLRIKKANLA	562-576

16	Mbov_0515[a,b]	AFM51869_1	GDHKWYKQLAVQEGQ	661-675
			DHKWYKQLAVQEGQQ	662-676
			DNYNSIDVPLAVDEK	597-611
			NFTKFSFSKPSGIIK	489-503
			LDNYNSIDVPLAVDE	596-610
			PNFTKFSFSKPSGII	488-502
			NGDHKWYKQLAVQEG	660-674
			DPNFTKFSFSKPSGI	487-501
			NYNSIDVPLAVDEKI	598-612
			FTKFSFSKPSGIIKT	490-504
			YLDNYNSIDVPLAVD	595-609
			YNSIDVPLAVDEKIK	599-613
			HKWYKQLAVQEGQQP	663-677
			TKFSFSKPSGIIKTV	491-505
17	Mbov_0516[a,b]	AFM51870_1	LNKYYQYSDALKAGG	702-716
			NLNKYYQYSDALKAG	701-715
			DNLNKYYQYSDALKA	700-714
			NKYYQYSDALKAGGK	703-717
			KYYQYSDALKAGGKK	704-718
			VSSQYPPNTPIATRI	531-545
			YNVSSQYPPNTPIAT	529-543
			NVSSQYPPNTPIATR	530-544
			SSQYPPNTPIATRIS	532-546
			SQYPPNTPIATRISF	533-547
			KVEEVPKPPTAAPEP	97-111

18	Mbov_0517[a,b]	AFM51871_1	GGVLSIASAVAIYKA	19-33		
			EELRKKTVEKALTSG	179-193		
			TEELRKKTVEKALTS	178-192		
			LRKKTVEKALTSGDG	181-195		
			ELRKKTVEKALTSGD	180-194		
			IASAVAIYKATSDFN	24-38		
			VTEELRKKTVEKALT	177-191		
			ASAVAIYKATSDFNF	25-39	VLSIASAVAIYKATS	21-35
			QRRQITLNGVQVYAT	126-140	LSIASAVAIYKATSD	22-36
			NTQRRQITLNGVQVY	124-138	SIASAVAIYKATSDF	23-37
			QLELFFSANATNTSS	465-479	GVLSIASAVAIYKAT	20-34
			LELFFSANATNTSSL	466-480		
			ELFFSANATNTSSLI	467-481		
			TQRRQITLNGVQVYA	125-139		
			PQLELFFSANATNTS	464-478		
			RRQITLNGVQVYATV	127-141		
			LFFSANATNTSSLIA	468-482		
			PNTQRRQITLNGVQV	123-137		
19	Mbov_0518[b]	AFM51872_1	TNSSAKTGLAAVFRS	765-779		
			MAKMKKILTLSTIPF	1-15		
			NSSAKTGLAAVFRSY	766-780		
			NPMYMAMLKNILSQG	190-204		
			TTNSSAKTGLAAVFR	764-778		
20	Mbov_0519[a,b]	AFM51873_1	NVSRDFRPNTSIPTR	519-533		
			VSRDFRPNTSIPTRI	520-534		
			SRDFRPNTSIPTRIT	521-535		
			YNVSRDFRPNTSIPT	518-532		
21	Mbov_0536[a,b]	AFM51890_1	LIFDSNKAIKILKYI	224-238		
			FDSNKAIKILKYIED	226-240		
			IFDSNKAIKILKYIE	225-239		
			YLIFDSNKAIKILKY	223-237		
			DSNKAIKILKYIEDD	227-241		
			KAKNAIQKHLSADWY	114-128		

			GTLSSAKAKIRIATS	288-302		
			TLSSAKAKIRIATSL	289-303		
			DGTLSSAKAKIRIAT	287-301		
			SGGYASARAFYADNV	461-475		
			QLFSGGYASARAFYA	458-472		
			GGYASARAFYADNVV	462-476		
			FSGGYASARAFYADN	460-474		
			LFSGGYASARAFYAD	459-473		
			LGENRTIKVAPTVSA	576-590	LSSAKAKIRIATSLF	290-304
22	Mbov_0570[a,b]	AFM51924_1	IGFSALIPVLSARCN	15-29	SSAKAKIRIATSLFD	291-305
			QFTKQNIKAPAFATE	423-437	SAKAKIRIATSLFDD	292-306
			AIGFSALIPVLSARC	14-28	MKSRKIKALFISQAI	1-15
			DKQFTKQNIKAPAFA	421-435		
			KQFTKQNIKAPAFAT	422-436		
			LFSGGYASARAFYAD	459-473		
			FSGGYASARAFYADN	460-474		
			AKAKIRIATSLFDDK	293-307		
			GFSALIPVLSARCND	16-30		
			NSQLFSGGYASARAF	456-470		
			GENRTIKVAPTVSAD	577-591		

23	Mbov_0579[a,b]	AFM51933_1	LSSTAIPLLAAVSAK	11-25	GLSSTAIPLLAAVSA	10-24
			MIGLSSTAIPLLAAV	8-22	IPMLKSIQVMSANAP	172-186
			LKSIQVMSANAPVLQ	175-189	IGLSSTAIPLLAAVS	9-23
			MIPMLKSIQVMSANA	171-185	PMLKSIQVMSANAPV	173-187
			FMIPMLKSIQVMSAN	170-184		
			LMIGLSSTAIPLLAA	7-21		
			MLKSIQVMSANAPVL	174-188		
			TFMIPMLKSIQVMSA	169-183		
			YGSLASRLASSEMRD	112-126		
			NYGSLASRLASSEMR	111-125		
			FNYGSLASRLASSEM	110-124		
			AFNYGSLASRLASSE	109-123		
			KLMIGLSSTAIPLLA	6-20		
			LAFNYGSLASRLASS	108-122		
			STAIPLLAAVSAKCG	13-27		
24	Mbov_0580[a,b]	AFM51934_1	FLKIYSLTPPSIDEN	86-100		
			SVYNSFESNKASAFE	155-169		
			NSVYNSFESNKASAF	154-168		
			YFLKIYSLTPPSIDE	85-99		
			DYFLKIYSLTPPSID	84-98		
			LKIYSLTPPSIDENT	87-101		
			VYNSFESNKASAFEF	156-170		
			KIYSLTPPSIDENTV	88-102		
			LNSVYNSFESNKASA	153-167		

			YEYGYLLALPPINFL	227-241
			EYGYLLALPPINFLR	228-242
			NYEYGYLLALPPINF	226-240
			NNYEYGYLLALPPIN	225-239
			YGYLLALPPINFLRN	229-243
			YGTTSIQIVKLIQDI	317-331
			KNYGTTSIQIVKLIQ	315-329
			NYGTTSIQIVKLIQD	316-330
25	Mbov_0585[a,b]	AFM51939_1	MKNYGTTSIQIVKLI	314-328
			SSSVSAFTLPIVSAS	12-26
			SSVSAFTLPIVSASC	13-27
			GYLLALPPINFLRNY	230-244
			SVSAFTLPIVSASCI	14-28
			KNNYEYGYLLALPPI	224-238
			TMKNYGTTSIQIVKL	313-327
			NKNNYEYGYLLALPP	223-237
			YLLALPPINFLRNYD	231-245
			DFNYFVLTAPVSFSA	436-450
26	Mbov_0658[a,b]	AFM52009_1	FNYFVLTAPVSFSAG	437-451
			YDFNYFVLTAPVSFS	435-449
			LIAGISVGAASGVAV	616-630

			EAQFNSGRSNASEHS	70-84
			EEAQFNSGRSNASEH	69-83
			KAYFKTQALASVIFN	107-121
			KEEAQFNSGRSNASE	68-82
			AQFNSGRSNASEHSG	71-85
			EKEEAQFNSGRSNAS	67-81
			AYFKTQALASVIFNQ	108-122
			FKWIYTYAGLVISSS	16-30
			YFKTQALASVIFNQK	109-123
27	Mbov_0674[a,b]	AFM52024_1	KFKWIYTYAGLVISS	15-29
			KKFKWIYTYAGLVIS	14-28
			KWIYTYAGLVISSSS	17-31
			SKAYFKTQALASVIF	106-120
			WIYTYAGLVISSSSL	18-32
			KELSFEGAGSSELNE	262-276
			NKELSFEGAGSSELN	261-275
			ELSFEGAGSSELNEA	263-277
			LSFEGAGSSELNEAH	264-278
			SNKELSFEGAGSSEL	260-274

28	Mbov_0675[a,b]	AFM52025_1	LGLGAISAFAAIPMV	7-21	GAISAFAAIPMVAAT	10-24
			VAKEMKYQAVAIGNH	184-198	LGAISAFAAIPMVAA	9-23
			KFILGLGAISAFAAI	4-18	AISAFAAIPMVAATC	11-25
			AKEMKYQAVAIGNHE	185-199	ISAFAAIPMVAATCG	12-26
			KVAKEMKYQAVAIGN	183-197	GLGAISAFAAIPMVA	8-22
			SKVAKEMKYQAVAIG	182-196	SAFAAIPMVAATCGV	13-27
			AFAAIPMVAATCGVS	14-28		
			ASKVFSGAYAQYSHN	548-562		
			SSPKQVAKARAAYDE	28-42		
			GASKVFSGAYAQYSH	547-561		
			RKFILGLGAISAFAA	3-17		
			HGASKVFSGAYAQYS	546-560		
			GNRIAAVQVKGSVLL	527-541		
			FAAIPMVAATCGVSS	15-29		
			FGNRIAAVQVKGSVL	526-540		
			VSSPKQVAKARAAYD	27-41		
			VKGSVLLEAMKHGAS	535-549		
			NRIAAVQVKGSVLLE	528-542		
			ILGLGAISAFAAIPM	6-20		
			PFGNRIAAVQVKGSV	525-539		
			GAISAFAAIPMVAAT	10-24		
			KVFSGAYAQYSHNVS	550-564		
			LGAISAFAAIPMVAA	9-23		
			SKVFSGAYAQYSHNV	549-563		
			AISAFAAIPMVAATC	11-25		

29	Mbov_0739[a,b]	AFM52087_1	MLRSAQLSLESLLKV	546-560
			LRSAQLSLESLLKVE	547-561
			NMLRSAQLSLESLLK	545-559
			RSAQLSLESLLKVEK	548-562
			ARTSGYIMPLASVVT	493-507
			RTSGYIMPLASVVTE	494-508
			IARTSGYIMPLASVV	492-506
			TSQMSNTVMQAMIEQ	576-590
			SQMSNTVMQAMIEQT	577-591
			TSGYIMPLASVVTEA	495-509
			KTSQMSNTVMQAMIE	575-589
			QIFRFQSAISFAASV	378-392
			SGYIMPLASVVTEAT	496-510
			LNMLRSAQLSLESLL	544-558
			WQIFRFQSAISFAAS	377-391
			SWQIFRFQSAISFAA	376-390
			QMSNTVMQAMIEQTE	578-592
			IFRFQSAISFAASVG	379-393
			KIFIKAGAILLPAAA	3-17
			FRFQSAISFAASVGA	380-394
			IFRFQSAISFAASVG	379-393
			QIFRFQSAISFAASV	378-392
			DKTSQMSNTVMQAMI	574-588
			FRFQSAISFAASVGA	380-394
			SAQLSLESLLKVEKG	549-563
			YGEYQLLKKVAHAIE	94-108
			EYGEYQLLKKVAHAI	93-107
			QSAISFAASVGAYQN	383-397
			SAISFAASVGAYQNK	384-398
			IKAGAILLPAAAGLS	6-20
			KAGAILLPAAAGLSV	7-21
			KLNMLRSAQLSLESL	543-557
			KKIFIKAGAILLPAA	2-16
			WQIFRFQSAISFAAS	377-391

			DDKTSQMSNTVMQAM	573-587
30	Mbov_0743[a,b]	AFM52091_1	GVNFLPYNSSAIINK	269-283
			DGVNFLPYNSSAIIN	268-282
			VNFLPYNSSAIINKF	270-284
			HDGVNFLPYNSSAII	267-281
31	Mbov_0798[a,b]	AFM52146_1	GSVASLASIPFVAAK	10-24
			SVASLASIPFVAAKC	11-25
			LGSVASLASIPFVAA	9-23
			VASLASIPFVAAKCG	12-26
			KIEAAIKEAIVAKVP	154-168
			EKIEAAIKEAIVAKV	153-167
			AAIKEAIVAKVPTLK	157-171
			LLGSVASLASIPFVA	8-22
			EAAIKEAIVAKVPTL	156-170
			KIEAAIKEAIVTKVP	68-82
			SKFLLLGSVASLASI	4-18
			IEAAIKEAIVAKVPT	155-169

a: related to *M. bovis* HB0801-P1, b: related to P150 and a,b: related to both (*M. bovis* HB0801-P1, and P150).

Table S7 Overlapped MHC class I and II T cell epitopes in recognized *M. bovis* secreted antigenic proteins predicted by using IEDB-AR.

No	Proteins		MHC class I		MHC class II	
	Mnemonic	Accession No	Sequence	Position	Sequence	Position
1	Mbov_0016[a,b]	AFM51394_1	KFYLFLGAA	5-13	KFYLFLGAAPVLSVP	5-19
					NKFYLFLGAAPVLSV	4-18
					KNKFYLFLGAAPVLS	3-17
			AAPVLSVPL	12-20	FYLFLGAAPVLSVPL	6-20
			FDTKQAAYI	200-208	FDTKQAAYIAGRALA	200-214
			AAYIAGRAL	205-213	QAAYIAGRALADYFS	204-218
					KQAAYIAGRALADYF	203-217
			ASYPVAGSL	296-304	IKATASYPVAGSLSS	292-306
					KATASYPVAGSLSSD	293-307
					SVIKATASYPVAGSL	290-304
			GEPVAVAAI	280-288	KTSFTSGEPVAVAAI	274-288
					TSFTSGEPVAVAAIN	275-289

			NYAGARKAI	477-485	TDLRNYAGARKAIED	473-487
					ETDLRNYAGARKAIE	472-486
					DLRNYAGARKAIEDA	474-488
					METDLRNYAGARKAI	471-485
					LRNYAGARKAIEDAD	475-489
			WEIGAEDTI	2851-2859	RKKWEIGAEDTIKTY	2848-2862
					DRKKWEIGAEDTIKT	2847-2861
			KAITLVDSL	2874-2882	KFEVDLKAITLVDSL	2868-2882
			YTYNFAEVI	2945-2953	LFSDYTYNFAEVINR	2941-2955
					FSDYTYNFAEVINRD	2942-2956
2	Mbov_0038[a,b]	AFM51416_1			ELFSDYTYNFAEVIN	2940-2954
					SDYTYNFAEVINRDN	2943-2957
					NELFSDYTYNFAEVI	2939-2953
					DYTYNFAEVINRDNL	2944-2958
					YTYNFAEVINRDNLQ	2945-2959
			KYQAAWVTS	1747-1755	ISKYQAAWVTSPFID	1745-1759
					DISKYQAAWVTSPFI	1744-1758
					SKYQAAWVTSPFIDV	1746-1760
					DISKYQAAWVTSPFI	1744-1758
					RDISKYQAAWVTSPF	1743-1757
			VDLKAITLV	2871-2879	EKFEVDLKAITLVDS	2867-2881
					KFEVDLKAITLVDSL	2868-2882
					YHKYQSSEATMRLFD	271-285
			KYQSSEATM	273-281	HKYQSSEATMRLFDD	272-286
					YYHKYQSSEATMRLF	270-284
					KYQSSEATMRLFDDE	273-287
					LYYHKYQSSEATMRL	269-283
3	Mbov_0049[a,b]	AFM51424_1			RLYYHKYQSSEATMR	268-282
					YYHKYQSSEATMRLF	270-284
					ESYESQLVQVVDAYE	376-390
			YESQLVQVV	378-386	RESYESQLVQVVDAY	375-389
			SYESQLVQV	377-385	SYESQLVQVVDAYEE	377-391
					LRESYESQLVQVVDA	374-388

			AGRYYYAGW	425-433	AGRYYYAGWNPSNRE	425-439
4	Mbov_0111[a,b]	AFM51486_1	YYYAGWNPS	428-436	TAGRYYYAGWNPSNR	424-438
					GRYYYAGWNPSNREE	426-440
			SAVGLATPL	428-436	FLYLGTSAVGLATPL	9-23
					SSLKYNTSSSAPNDS	365-379
5	Mbov_0154[a,b]	AFM51527_1	KYNTSSSAP	368-376	SLKYNTSSSAPNDSY	366-380
					PSSLKYNTSSSAPND	364-378
					IPSSLKYNTSSSAPN	363-377
			KAIEAFAHL	427-435	SKNLLEKAIEAFAHL	421-435
			IEAFAHLGV	429-437	KNLLEKAIEAFAHLG	422-436
6	Mbov_0217[a,b]	AFM51586_1			NLLEKAIEAFAHLGV	423-437
			KEINNMLLV	164-172	RKEINNMLLVKAYFD	163-177
					KEINNMLLVKAYFDI	164-178
					KKVVFQFAQSNSWPL	29-43
					KVVFQFAQSNSWPLP	30-44
			FAQSNSWPL	35-43	VVFQFAQSNSWPLPK	31-45
					FQFAQSNSWPLPKML	33-47
					VFQFAQSNSWPLPKM	32-46
					LNFLRSAYVSIKSIR	517-531
					IIPLAVAAPMIAASC	10-24
7	Mbov_0274[a]	AFM51642_1	LNFLRSAYV	517-525	GIIPLAVAAPMIAAS	9-23
			IPLAVAAPM	11-19	IPLAVAAPMIAASCQ	11-25
					FGIIPLAVAAPMIAA	8-22
					AKYSGYIMPLASVVN	472-486
					FAKYSGYIMPLASVV	471-485
			GYIMPLASV	476-484	KYSGYIMPLASVVND	473-487
					YSGYIMPLASVVNDE	474-488
					SGYIMPLASVVNDES	475-489
8	Mbov_0290[b]	AFM51658_1				

					IPYVSVKQALSALEG	69-83
					PYVSVKQALSALEGV	70-84
					RIPYVSVKQALSALE	68-82
					KNYQIYHANGASLKF	102-116
					NKNYQIYHANGASLK	101-115
			PYVSVKQAL	70-78	NYQIYHANGASLKFN	103-117
					YQIYHANGASLKFNW	104-118
			IYHANGASL	106-114	LLTSNLSFSAANALV	520-534
9	Mbov_0296[b]	AFM51664_1			FLLTSNLSFSAANAL	519-533
					LTSNLSFSAANALVA	521-535
					TSNLSFSAANALVAY	522-536
					SNLSFSAANALVAYT	523-537
			LSFSAANAL	525-533	NLSFSAANALVAYTA	524-538
			FSAANALVA	527-535	LSFSAANALVAYTAA	525-539
					LSFSAANALVAYTAA	525-539
					NLSFSAANALVAYTA	524-538
					SFSAANALVAYTAAD	526-540
					FSAANALVAYTAADK	527-541
10	Mbov_0350[a,b]	AFM51716_1				
11	Mbov_0364[a,b]	AFM51726_1				
12	Mbov_0374[b]	AFM51736_1				
					SKYLQTVDYVSAFKT	343-357
					FSSLYYGASGSLAYN	471-485
13	Mbov_0468[a,b]	AFM51824_1	KYLQTVDYV	344-352	NFSSLYYGASGSLAY	470-484
			LYYGASGSL	474-482	SSLYYGASGSLAYNE	472-486
					SLYYGASGSLAYNEY	473-487
					LYYGASGSLAYNEYG	474-488

14	Mbov_0471[a,b]	AFM51827_1	PYAFSAGNI	446-454	YVLTSPYAFSAGNIF	441-455
					LTSPYAFSAGNIFPQ	443-457
			IGYKTFGGA	468-476	IGYKTFGGASAISYA	468-482
					VIGYKTFGGASAISY	467-481
					KVIGYKTFGGASAIS	466-480
					GKVIGYKTFGGASAI	465-479
			ASAISYAIL	476-484	YKTFGGASAISYAIL	470-484
15	Mbov_0505[a,b]	AFM51861_1	DESALKEFL	33-41	FDESALKEFLIIPLA	32-46
			IAKSFKISL	97-105	IAKSFKISLPKPSNS	97-111
					PIAKSFKISLPKPSN	96-110
					PPIAKSFKISLPKPS	95-109
16	Mbov_0515[a,b]	AFM51869_1	NYNSIDVPL	598-606	DNYNSIDVPLAVDEK	597-611
					LDNYNSIDVPLAVDE	596-610
					NYNSIDVPLAVDEKI	598-612
					YLDNYNSIDVPLAVD	595-609
			FSFSKPSGI	493-501	NFTKFSFSKPSGIIK	489-503
					PNFTKFSFSKPSGII	488-502
					DPNFTKFSFSKPSGI	487-501
					FTKFSFSKPSGIIKT	490-504
					TKFSFSKPSGIIKTV	491-505
17	Mbov_0516[a,b]	AFM51870_1	KYYQYSDAL	704-712	LNKYYQYSDALKAGG	702-716
					NLNKYYQYSDALKAG	701-715
					DNLNKYYQYSDALKA	700-714
					NKYYQYSDALKAGGK	703-717
					KYYQYSDALKAGGKK	704-718
			SQYPPNTPI	533-541	VSSQYPPNTPIATRI	531-545
					YNVSSQYPPNTPIAT	529-543
					NVSSQYPPNTPIATR	530-544
					SSQYPPNTPIATRIS	532-546
			TPIATRISF	539-547	SQYPPNTPIATRISF	533-547
18	Mbov_0517[a,b]	AFM51871_1	TEELRKKTV	178-186	TEELRKKTVEKALTS	178-192
					VTEELRKKTVEKALT	177-191
19	Mbov_0518[b]	AFM51872_1	MYMAMLKNI	192-200	NPMYMAMLKNILSQG	190-204

					NVSRDFRPNTSIPTR	519-533
20	Mbov_0519[a,b]	AFM51873_1	VSRDFRPNT	520-528	VSRDFRPNTSIPTRI	520-534
					YNVSRDFRPNTSIPT	518-532
21	Mbov_0536[a,b]	AFM51890_1	FDSNKAIKI	226-234	LIFDSNKAIKILKYI	224-238
22	Mbov_0570[a,b]	AFM51924_1	IGFSALIPV	15-23	IGFSALIPVLSARCN	15-29
					AIGFSALIPVLSARC	14-28
			TAIPLLAAV	14-22	LSSTAIPLLAAVSAK	11-25
					MIGLSSTAIPLLAAV	8-22
					STAIPLLAAVSAKCG	13-27
					GLSSTAIPLLAAVSA	10-24
					IGLSSTAIPLLAAVS	9-23
23	Mbov_0579[a,b]	AFM51933_1	NYGSLASRL	111-119	NYGSLASRLASSEMR	111-125
					FNYGSLASRLASSEM	110-124
					AFNYGSLASRLASSE	109-123
					LAFNYGSLASRLASS	108-122
			FMIPMLKSI	170-178	FMIPMLKSIQVMSAN	170-184
					TFMIPMLKSIQVMSA	169-183
					SVYNSFESNKASAFE	155-169
24	Mbov_0580[a,b]	AFM51934_1	FESNKASAF	160-168	NSVYNSFESNKASAF	154-168
					VYNSFESNKASAFEF	156-170
					YEYGYLLALPPINFL	227-241
			YEYGYLLAL	227-235	EYGYLLALPPINFLR	228-242
			NNYEYGYLL	225-233	NYEYGYLLALPPINF	226-240
			GYLLALPPI	230-238	NNYEYGYLLALPPIN	225-239
			KNNYEYGYL	224-232	YGYLLALPPINFLRN	229-243
25	Mbov_0585[a,b]	AFM51939_1			GYLLALPPINFLRNY	230-244
					KNNYEYGYLLALPPI	224-238
					TMKNYGTTSIQIVKL	313-327
					NKNNYEYGYLLALPP	223-237
					KNYGTTSIQIVKLIQ	315-329
			NYGTTSIQI	316-324	NYGTTSIQIVKLIQD	316-330
					MKNYGTTSIQIVKLI	314-328

					DF<u>NYFVLTAPV</u>SFSA	436-450
26	Mbov_0658[a,b]	AFM52009_1	NYFVLTAPV	438-446	F<u>NYFVLTAPV</u>SFSAG	437-451
					YDF<u>NYFVLTAPV</u>SFS	435-449
					F<u>KWIYTYAGLVI</u>SSS	16-30
					KF<u>KWIYTYAGLVI</u>SS	15-29
			WIYTYAGLV	18-26	KKF<u>KWIYTYAGLVI</u>S	14-28
			IYTYAGLVI	19-27	<u>KWIYTYAGLVI</u>SSSS	17-31
			KWIYTYAGL	17025	<u>WIYTYAGLVI</u>SSSSL	18-32
27	Mbov_0674[a,b]	AFM52024_1			<u>KAYFKTQAL</u>ASVIFN	107-121
					KELS<u>FEGAGSSEL</u>NE	262-276
			KAYFKTQAL	107-115	NKELS<u>FEGAGSSEL</u>N	261-275
			FEGAGSSEL	266-274	ELS<u>FEGAGSSEL</u>NEA	263-277
					LS<u>FEGAGSSEL</u>NEAH	264-278
					SNKELS<u>FEGAGSSEL</u>	260-274
			SAFAAIPMV	13-21	LGLGAI<u>SAFAAIPMV</u>	7-21
					GAI<u>SAFAAIPMV</u>AAT	10-24
					LGAI<u>SAFAAIPMV</u>AA	9-23
					AI<u>SAFAAIPMV</u>AATC	11-25
					GAI<u>SAFAAIPMV</u>AAT	10-24
					LGAI<u>SAFAAIPMV</u>AA	9-23
28	Mbov_0675[a,b]	AFM52025_1			AI<u>SAFAAIPMV</u>AATC	11-25
					I<u>SAFAAIPMV</u>AATCG	12-26
					GLGAI<u>SAFAAIPMV</u>A	8-22
					<u>SAFAAIPMV</u>AATCGV	13-27
			AYAQYSHNV	555-563	KVFSG<u>AYAQYSHNV</u>S	550-564
					SKVFSG<u>AYAQYSHNV</u>	549-563
			KFILGLGAI	4-12	<u>KFILGLGAI</u>SAFAAI	4-18
					R<u>KFILGLGAI</u>SAFAA	3-17

			SAISFAASV	384-392	QIFRFQSAISFAASV	378-392
			QIFRFQSAI	378-386	IFRFQSAISFAASVG	379-393
					FRFQSAISFAASVGA	380-394
					IFRFQSAISFAASVG	379-393
					QIFRFQSAISFAASV	378-392
					FRFQSAISFAASVGA	380-394
					QSAISFAASVGAYQN	383-397
					SAISFAASVGAYQNK	384-398
					WQIFRFQSAISFAAS	377-391
					SWQIFRFQSAISFAA	376-390
29	Mbov_0739[a,b]	AFM52087_1	GEYQLLKKV	95-103	YGEYQLLKKVAHAIE	94-108
					EYGEYQLLKKVAHAI	93-107
			LNMLRSAQL	544-552	LNMLRSAQLSLESLL	544-558
					KLNMLRSAQLSLESL	543-557
			GYIMPLASV	497-505	ARTSGYIMPLASVVT	493-507
					RTSGYIMPLASVVTE	494-508
					IARTSGYIMPLASVV	492-506
					TSGYIMPLASVVTEA	495-509
					SGYIMPLASVVTEAT	496-510
			LPAAAGLSV	13-21	KAGAILLPAAAGLSV	7-21
					GVNFLPYNSSAIINK	269-283
30	Mbov_0743[a,b]	AFM52091_1	VNFLPYNSS	270-278	DGVNFLPYNSSAIIN	268-282
			LPYNSSAII	273-281	VNFLPYNSSAIINKF	270-284
					HDGVNFLPYNSSAII	267-281
					KIEAAIKEAIVAKVP	154-168
			IEAAIKEAI	155-163	EKIEAAIKEAIVAKV	153-167
31	Mbov_0798[a,b]	AFM52146_1	IEAAIKEAI	69-77	KIEAAIKEAIVTKVP	68-82
					IEAAIKEAIVAKVPT	155-169

a: related to *M. bovis* HB0801-P1, b: related to P150 and a,b: related to both (*M. bovis* HB0801-P1, and P150).

Table S8 The 3D structure templates used for recognition of conformational B cell epitopes at minimum cut-off 0.8 for *M. bovis* secreted proteins

No	Proteins		PDB ID	Seq Identity/ Percentage	Epitope	Residue	No	Score	3D Structure
	Mnemonic	Accession No							
					1	A:Q102, A:V103, A:S104, A:G105, A:N106, A:K107, A:N108, A:L109, A:R110, A:N111	10	0.892	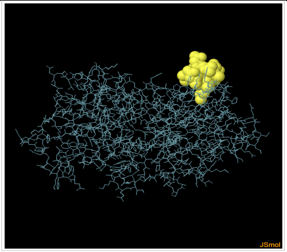
1	Mbov_0016[a,b]	AFM51394_1	2fqx	20.61	2	A:N162, A:S359, A:Q360, A:G361, A:E362, A:N363, A:S364, A:L365, A:S366, A:L367, A:Q368, A:P369, A:G370	13	0.866	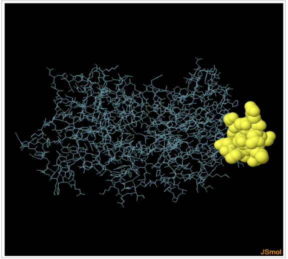
					3	A:S218, A:Y221, A:K222, A:D223, A:N224, A:P225, A:E226, A:K227, A:E258, A:H259, A:P260, A:E261, A:A262, A:K263, A:T264, A:K265, A:S266, A:L267	18	0.862	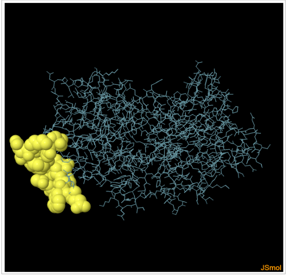

Formatted Table

					4	A:D175, A:A176, A:S177, A:T178, A:G179, A:E180, A:P181, A:A182, A:A185, A:F186, A:V187, A:D188, A:K189, A:I190, A:G191, A:Q192	16	0.844	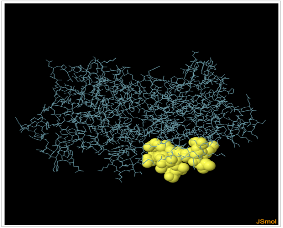
					5	A:D407, A:E408, A:I409, A:K412	4	0.834	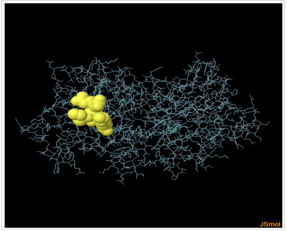
					6	A:K314, A:L315, A:G316, A:D317, A:K318, A:K320, A:K334, A:G335, A:H336	9	0.822	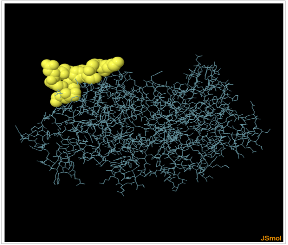
2	Mbov_0038[a,b]	AFM51416_1	4tql	31.11	1	A:A642, A:L643, A:L644	3	0.879	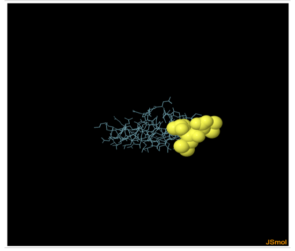

					2	A:T587, A:K589, A:N590	3	0.862	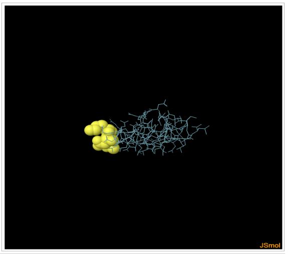
					1	A:Y706, A:L709, A:E710	3	0.967	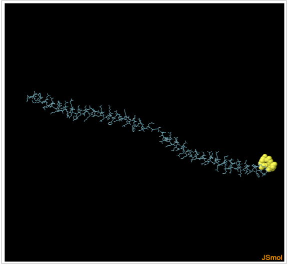
3	Mbov_0049[a,b]	AFM51424_1	1c1g	21.85					
					2	A:L700, A:S703, A:F704, A:E707, A:D708	5	0.95	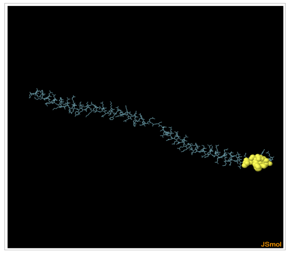

4	Mbov_0111[a,b]	AFM51486_1	3ryb	16.11	1	A:R517, A:D518, A:Y519	3	0.866	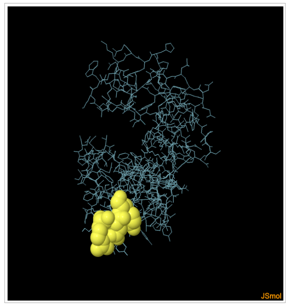
					2	A:G426, A:R427, A:N445, A:P446, A:H447, A:Y448, A:R449, A:K450, A:W460, A:K461, A:E462, A:S463, A:R464	13	0.841	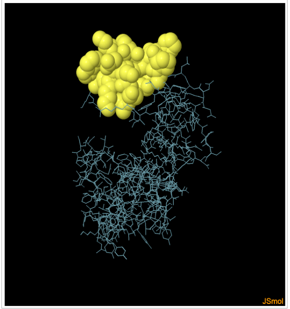
					3	A:A563, A:F564, A:G565, A:A566, A:S567, A:L568, A:K569, A:E570, A:I571, A:R572, A:E573, A:G574, A:G575, A:K576, A:P577, A:T578, A:N579, A:L580, A:K581, A:D582, A:K583, A:L584, A:T587	23	0.838	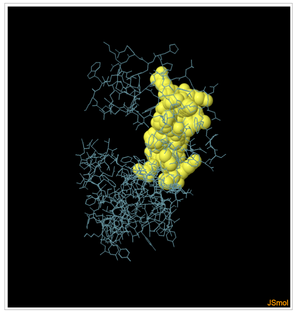

5	Mbov_0217[a,b]	AFM51586_1	2l69	22.45	1	A:L409, A:N410, A:K411, A:A413, A:Y414	5	0.841	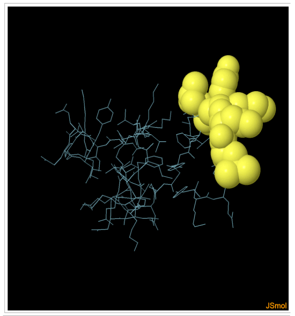
6	Mbov_0274[a]	AFM51642_1	4gqo	20.00	1	A:N168, A:L169, A:E170, A:V171, A:L172, A:D173, A:Y174	7	0.963	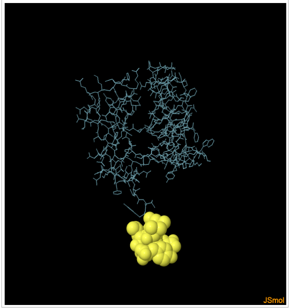
					2	A:A164, A:V165, A:V166, A:Y167	4	0.888	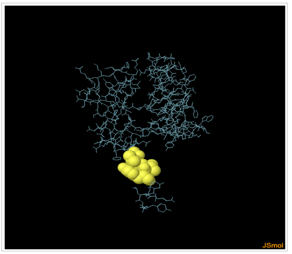

					3	A:Y53, A:T56, A:T57, A:F58, A:S59, A:K60, A:H61	7	0.848	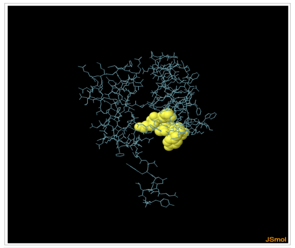
					4	A:K62, A:D63, A:F64	3	0.811	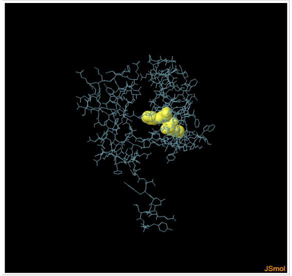
7	Mbov_0296[b]	AFM51664_1	4ghn	14.52	1	A:N401, A:K402, A:Q443, A:R445, A:S446, A:M447, A:E448, A:T449, A:G450, A:K451, A:E452, A:I453, A:N454	13	0.859	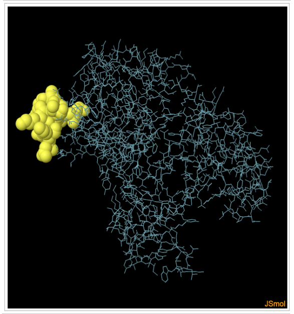
					2	A:Q241, A:D242, A:Q245, A:F249, A:D273, A:P274, A:S275, A:K277, A:K278	9	0.857	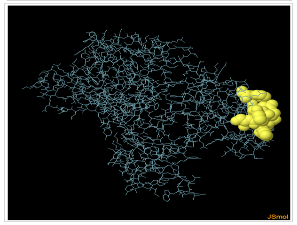

3	A:L281, A:N282, A:R283, A:D284, A:P285	5	0.852	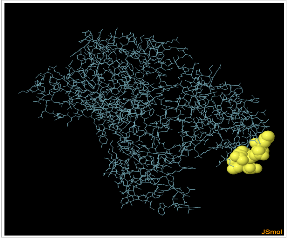
4	A:E235, A:T236, A:S237, A:A238, A:Q239, A:L568, A:S569, A:D570, A:G571, A:T572	10	0.815	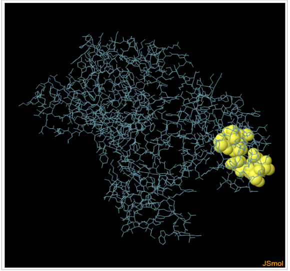
5	A:N322, A:Y323, A:I324, A:L327, A:V328, A:K329, A:H330, A:S331, A:L332, A:D333, A:E334, A:Y335, A:K336, A:N337, A:K338, A:V339, A:D340, A:P341, A:F343, A:N344, A:S345, A:K346, A:W347, A:L348, A:N349, A:A350, A:V378, A:S379, A:Q380, A:T381, A:I382, A:N383, A:G384, A:L385, A:R386, A:L387, A:V388	37	0.81	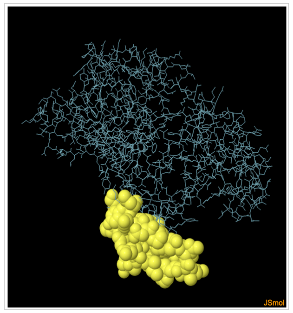

8	Mbov_0471[a,b]	AFM51827_1	1k32	15.41	1	C:K517, C:Y518, C:D519, C:L520, C:S521, C:K522, C:N523, C:P524, C:E525, C:K526, C:L527, C:Y528	12	0.935	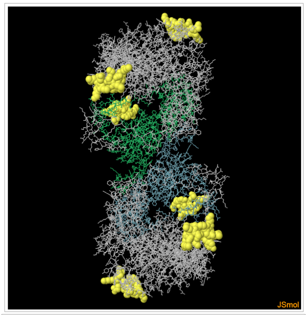
					2	A:I255, A:L257, A:D258, A:N260, A:K261, A:K264	6	0.894	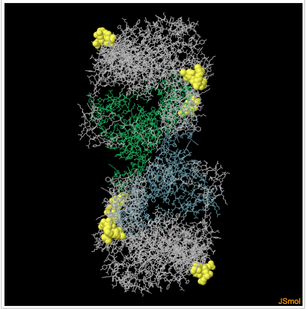
					3	B:I255, B:L257, B:D258, B:N260, B:K261, B:K264	6	0.894	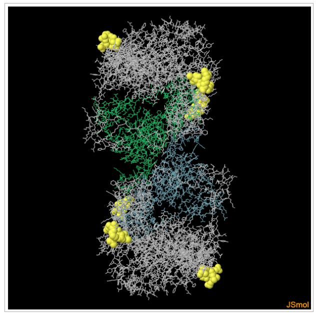

4	F:I255, F:L257, F:D258, F:N260, F:K261	5	0.887	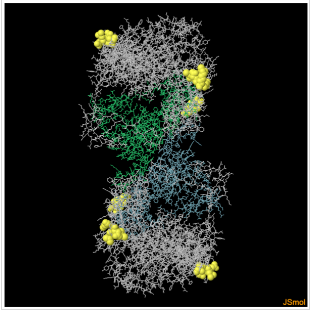
5	C:I255, C:L257, C:D258, C:N260, C:K261, C:S262, C:E265, C:F266	8	0.881	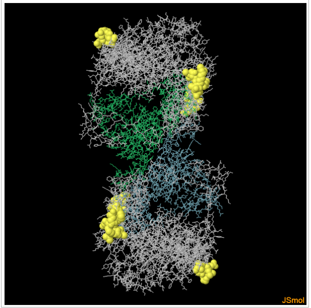
6	F:Y348, F:T349, F:P350, F:D351, F:N352, F:K353, F:K383, F:P446, F:G465, F:K466, F:G469, F:Y470, F:K471, F:T499, F:D500, F:K501, F:R504, F:F508, F:G509, F:I510, F:E511, F:P512, F:N513, F:F514, F:K515, F:F516, F:K517, F:Y518, F:D519, F:L520, F:S521, F:K522, F:N523, F:P524, F:E525, F:L527, F:Y528, F:D529, F:L530, F:T531, F:Q534, F:N535, F:V537, F:N538, F:N539, F:I540, F:S541, F:Q542	48	0.876	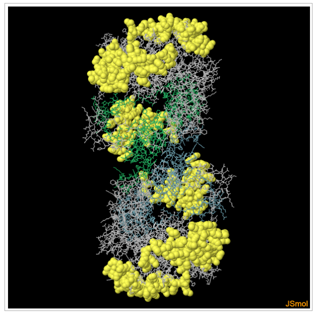

7	D:Y348, D:T349, D:P350, D:D351, D:N352, D:K353, D:T354, D:K383, D:P446, D:K466, D:G469, D:Y470, D:K471, D:T472, D:G509, D:I510, D:E511, D:P512, D:N513, D:F514, D:K515, D:F516, D:K517, D:Y518, D:D519, D:L520, D:S521, D:K522, D:N523, D:P524, D:E525, D:K526, D:Y528, D:D529, D:L530, D:T531, D:Q534, D:N535, D:V537, D:N538, D:N539, D:I540, D:S541, D:Q542	44	0.873	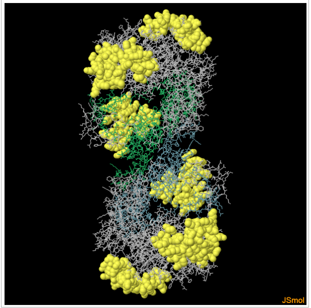
8	E:I255, E:L257, E:D258, E:N260, E:K261, E:S262, E:E265, E:F266, E:D292	9	0.87	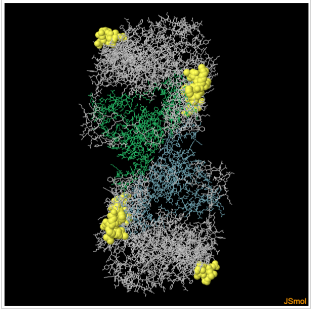

9	E:Y348, E:T349, E:P350, E:D351, E:N352, E:K353, E:T354, E:K383, E:P446, E:G465, E:K466, E:G469, E:Y470, E:K471, E:T472, E:G509, E:I510, E:E511, E:P512, E:N513, E:F514, E:K515, E:F516, E:K517, E:Y518, E:D519, E:L520, E:S521, E:K522, E:N523, E:P524, E:E525, E:L527, E:Y528, E:L530, E:T531, E:Q534, E:N535, E:V537, E:N538, E:N539, E:I540, E:S541, E:Q542	44	0.867	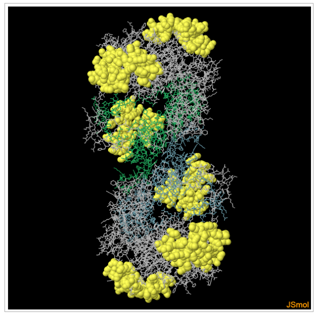
10	B:Y348, B:T349, B:P350, B:D351, B:N352, B:K353, B:V382, B:K383, B:F438, B:P446, B:G465, B:K466, B:G469, B:Y470, B:K471, B:T472, B:F508, B:G509, B:I510, B:E511, B:P512, B:N513, B:F514, B:K515, B:F516, B:K517, B:Y518, B:D519, B:L520, B:S521, B:K522, B:N523, B:P524, B:E525, B:K526, B:D529, B:L530, B:T531, B:Q534, B:N535, B:V537, B:N538, B:N539, B:I540, B:S541, B:Q542	46	0.864	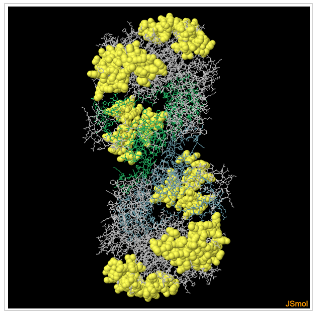

11	A:Y348, A:T349, A:P350, A:D351, A:N352, A:K353, A:T354, A:V382, A:K383, A:F438, A:P446, A:G465, A:K466, A:G469, A:Y470, A:K471, A:T472, A:G509, A:I510, A:E511, A:P512, A:N513, A:F514, A:K515, A:F516, A:K517, A:Y518, A:D519, A:L520, A:S521, A:K522, A:N523, A:P524, A:E525, A:K526, A:Y528, A:D529, A:L530, A:T531, A:Q534, A:N535, A:V537, A:N538, A:N539, A:I540, A:S541, A:Q542	47	0.861	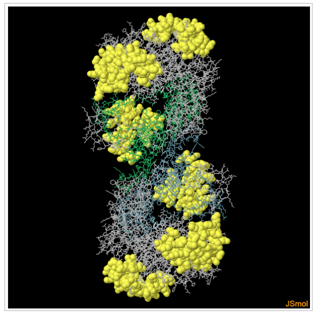
12	A:S262, A:E265, A:F266, A:D292	4	0.86	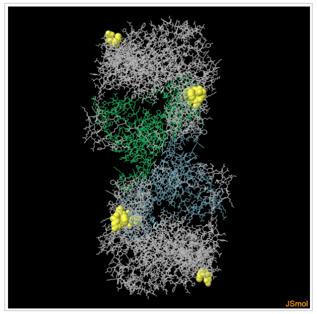

13	B:S262, B:E265, B:F266, B:D292	4	0.86	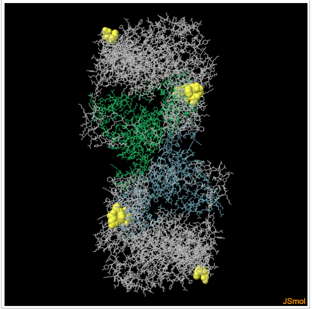
14	F:S262, F:E265, F:F266, F:D292	4	0.86	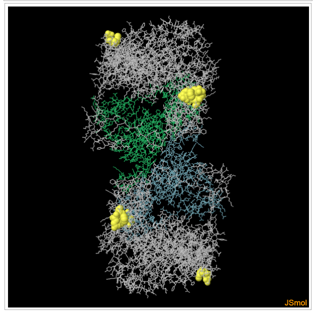
15	D:K261, D:S262, D:E265, D:F266, D:D292, D:L293	6	0.857	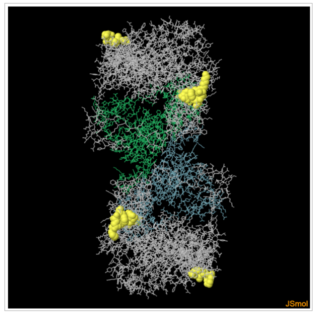

16	A:T499, A:D500, A:K501, A:H502, A:F503, A:R504	6	0.851	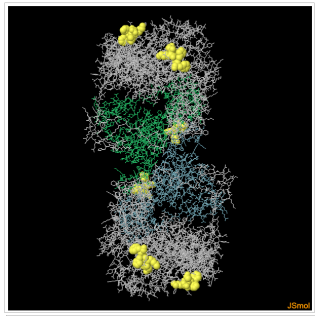
17	B:T499, B:D500, B:K501, B:H502, B:F503, B:R504	6	0.851	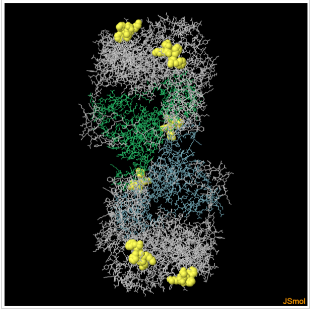
18	C:T499, C:D500, C:K501, C:H502, C:F503, C:R504	6	0.851	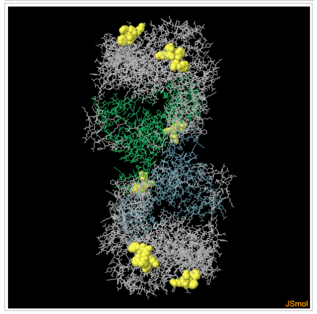

19	D:T499, D:D500, D:K501, D:H502, D:F503, D:R504	6	0.851	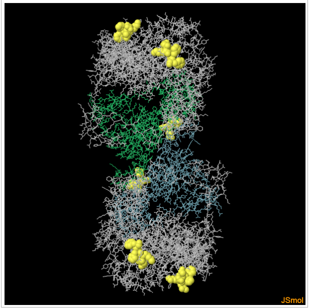
20	D:I255, D:L257, D:D258	3	0.849	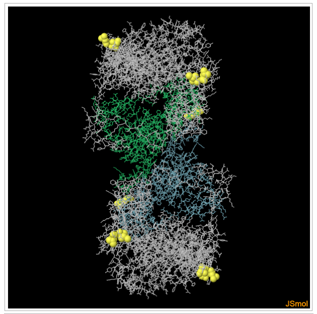
21	E:T499, E:D500, E:K501, E:H502, E:F503, E:R504	6	0.847	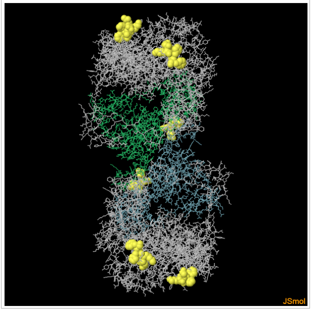

					22	C:Y348, C:T349, C:P350, C:D351, C:N352, C:K353, C:K383, C:F438, C:N462, C:N463, C:V464, C:G465, C:K466, C:G469, C:Y470, C:K471, C:T472, C:G509, C:I510, C:E511, C:P512, C:N513, C:F514, C:K515, C:F516, C:L530, C:T531, C:Q534, C:N535, C:V537, C:N538, C:N539, C:I540, C:S541, C:Q542	35	0.846	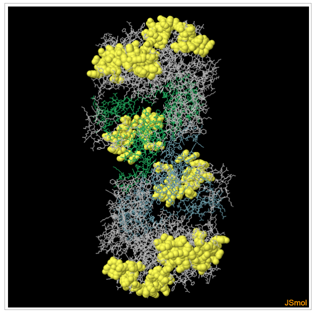
9	Mbov_0515[a,b]	AFM51869_1	2mi7	30.77	1	A:K398, A:K399, A:D400	3	0.803	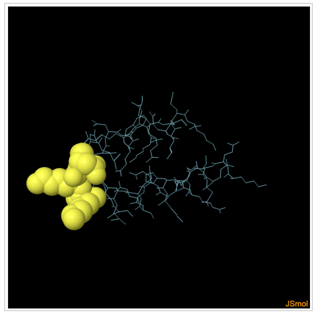
10	Mbov_0516[a,b]	AFM51870_1	1wpl	20.41	1	K:N743, K:G744, K:L745, K:T746, K:F747, K:T748	6	0.803	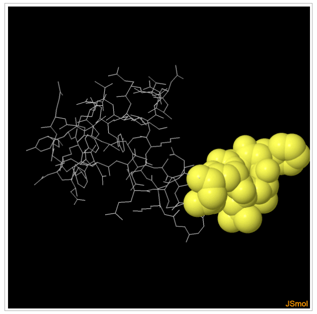

					1	C:D546, C:S547, C:D548, C:D549, C:Y550, C:K551, C:T552, C:E553, C:N554, C:N555, C:F556, C:E557	12	0.916	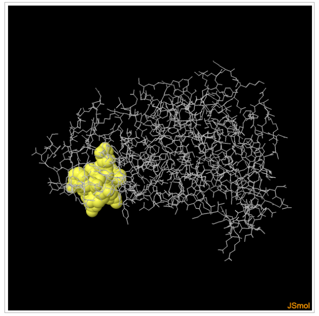
11	Mbov_0517[a,b]	AFM51871_1	4nzr	19.70	2	C:K636, C:A637, C:S638, C:S639, C:F640, C:S641	6	0.908	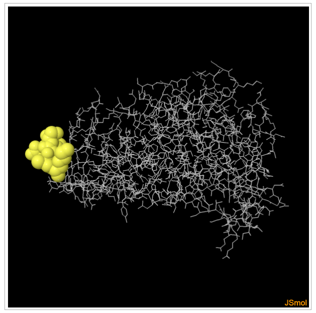
					3	C:I607, C:K608, C:R611	3	0.874	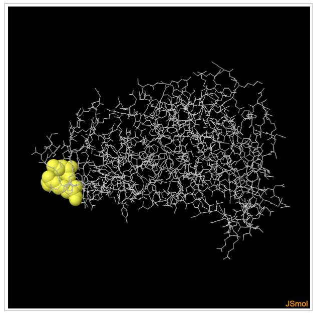

4	C:R323, C:E324, C:F325, C:N326, C:T327, C:V328, C:T329, C:S330, C:R331, C:G343, C:S385	11	0.833	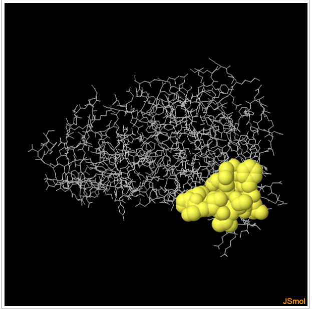
5	C:R413, C:N414, C:E416, C:G417, C:Y418, C:K419, C:T421, C:K422, C:E423, C:L424, C:I425, C:K427, C:F428	13	0.828	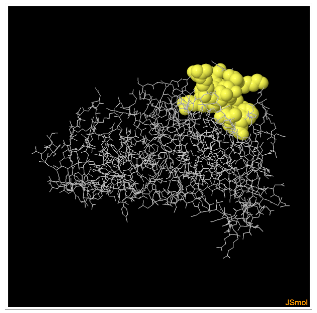
6	C:N380, C:N381, C:D382, C:G383, C:I384, C:H387, C:K394, C:I395, C:K396	9	0.822	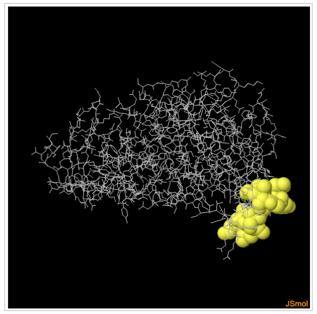

12	Mbov_0518[b]	AFM51872_1	5jwg	28.24	1	A:I711, A:T716, A:G717, A:S718, A:Y721	5	0.853	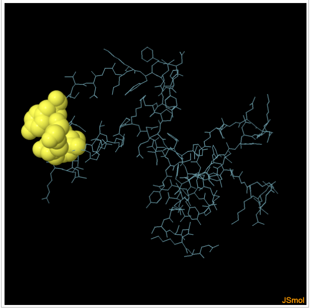
					2	A:E683, A:N684, A:L685	3	0.826	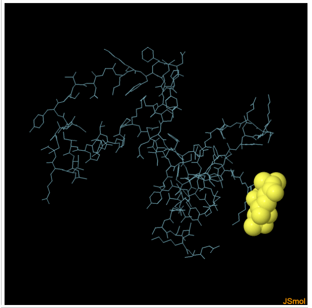
13	Mbov_0519[a,b]	AFM51873_1	4nzr	18.28	1	C:N322, C:T323, C:V324, C:T325, C:S326, C:R327, C:F338, C:K382, C:T384, C:K385, C:R388	11	0.857	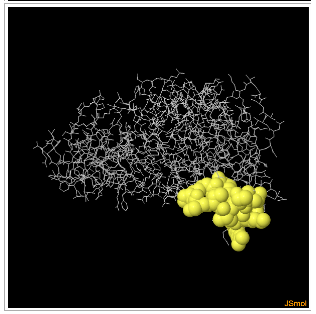

2	C:E600, C:G601, C:I602, C:K603, C:R606, C:L628, C:F629, C:N630, C:N631, C:S632, C:E633, C:A634, C:F635, C:E636	14	0.834	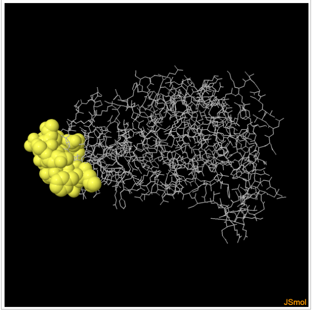
3	C:A540, C:D542, C:S543, C:D544, C:D545, C:F546, C:K547, C:N548, C:K549, C:S550, C:F551, C:E552, C:R598, C:V599	14	0.828	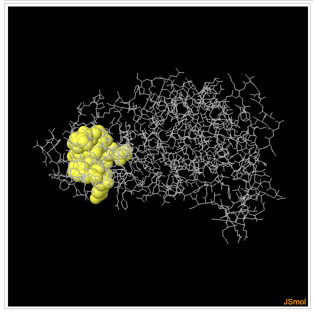
4	C:S377, C:A378, C:D379, C:G380, C:I381	5	0.813	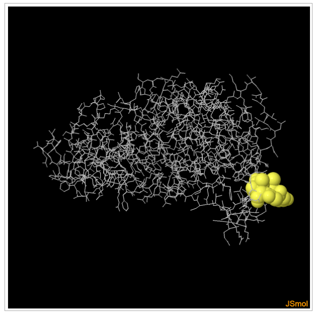

					5	C:S412, C:G413, C:Y414, C:D415, C:K416, C:T417, C:L418, C:K419, C:D450	9	0.804	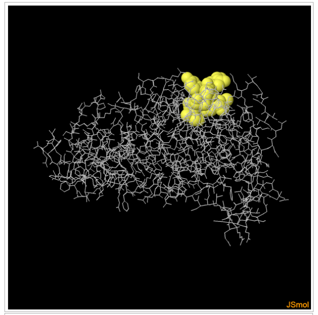
14	Mbov_0570[a,b]	AFM51924_1	1f3z	23.96	1	A:F492, A:Q493, A:P494, A:V495	4	0.822	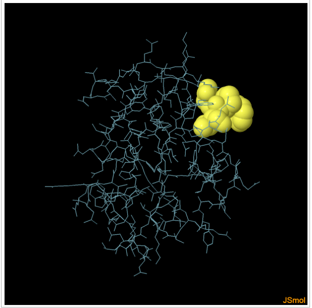
15	Mbov_0579[a,b]	AFM51933_1	4uac	13.33	1	A:Y699, A:L700, A:K701	3	0.896	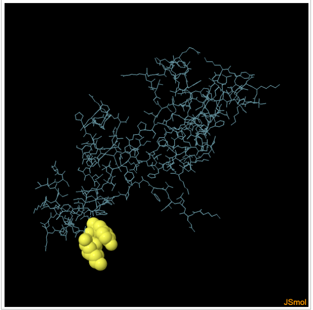

2	A:W558, A:S561, A:N562, A:E563	4	0.854	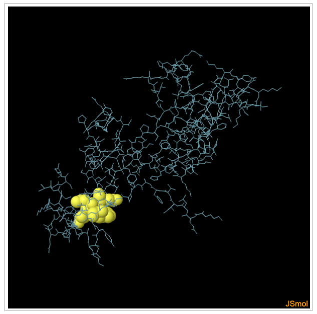
3	A:V577, A:S578, A:R579, A:G580, A:A581, A:K582, A:P583, A:D584	8	0.853	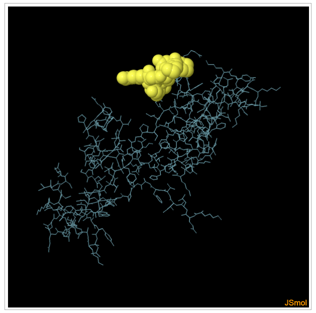
4	A:S697, A:A698, A:A702, A:K703, A:N704, A:D705, A:Q706, A:P707, A:L708, A:E709, A:D710, A:K713	12	0.829	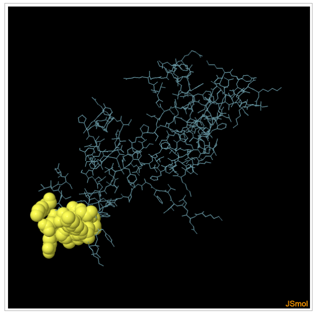

					1	A:T334, A:N335, A:K336, A:K337, A:K341	5	0.867	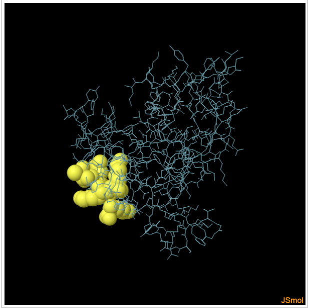
16	Mbov_0580[a,b]	AFM51934_1	1sta	24.44	2	A:S258, A:D281, A:K282, A:Y283	4	0.863	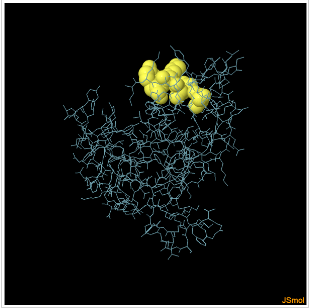
					3	A:A321, A:L322, A:L323	3	0.841	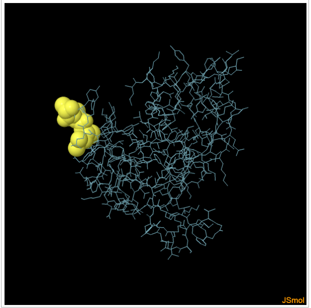

					4	A:K228, A:F229, A:V230, A:G231, A:G232, A:I233, A:K234, A:S235, A:S236, A:N237	10	0.841	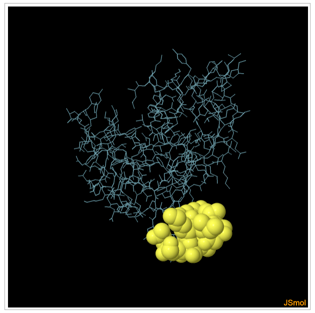
					5	A:F310, A:V311, A:K312	3	0.809	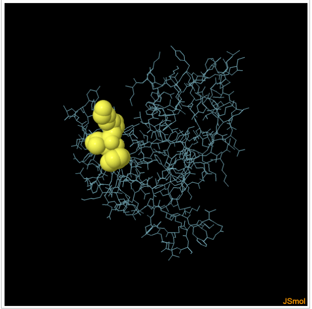
17	Mbov_0585[a,b]	AFM51939_1	5djn	26.47	1	A:F498, A:M499, A:E500, A:I502, A:N503, A:L504, A:G505	7	0.893	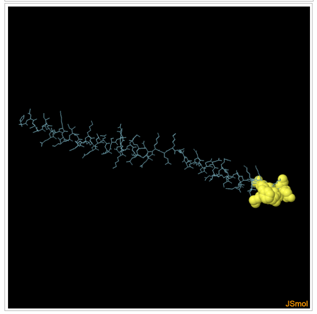

					1	A:I482, A:L483, A:P484, A:T485, A:G486, A:D487, A:I488, A:I489, A:Q490	9	0.945	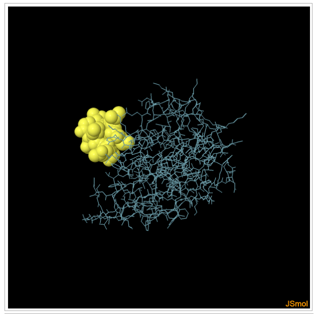
18	Mbov_0658[a,b]	AFM52009_1	4ql6	19.15	2	A:D350, A:G351, A:Q352, A:T353, A:K371, A:K374, A:E375, A:A376, A:S378, A:K379, A:G380, A:K382	12	0.864	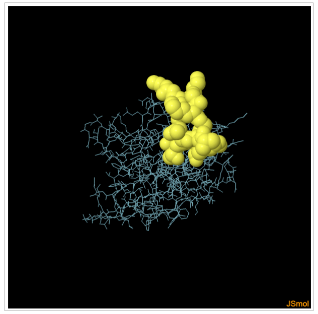
					3	A:I536, A:N537, A:N538, A:I539, A:S540, A:K541	6	0.846	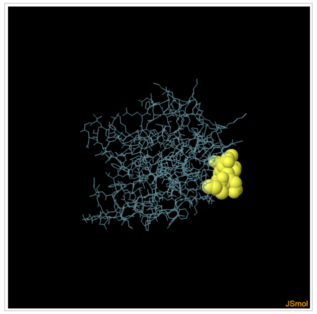

19	Mbov_0674[a,b]	AFM52024_1	4awn	20.32	4	A:P445, A:D516, A:E517, A:N518, A:I519, A:E520, A:T521, A:G522, A:A523, A:K524, A:N525, A:L526	12	0.808 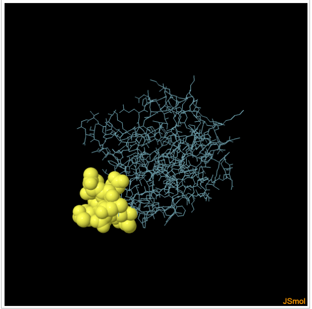
					1	A:F375, A:Y377, A:K378, A:N379, A:I380, A:N381, A:S382, A:L383, A:N384, A:D385, A:W386, A:Y389, A:V390, A:K391, A:S392, A:S393, A:S394, A:S395, A:K396, A:K397, A:K399, A:S400, A:N401, A:S402, A:G403	25	0.892 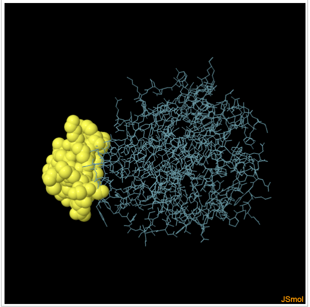
					2	A:P209, A:E210, A:K212, A:E213, A:T214, A:P215, A:F216, A:G217, A:G218, A:S219, A:V220, A:E267, A:G268	13	0.847 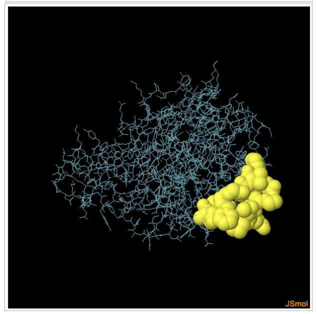

3	A:N148, A:E149, A:Q150, A:S151, A:Q152, A:I154, A:S155, A:S156, A:H157, A:D158	10	0.836	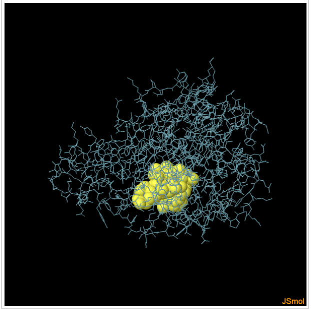
4	A:L235, A:D236, A:N237, A:S238, A:L239, A:K240, A:N242, A:S355, A:K356, A:L357	10	0.824	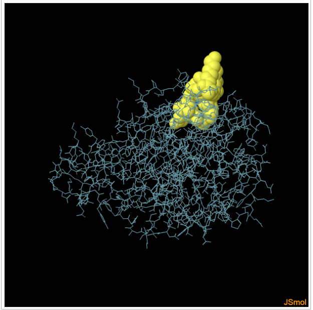
5	A:S106, A:K107, A:A108	3	0.819	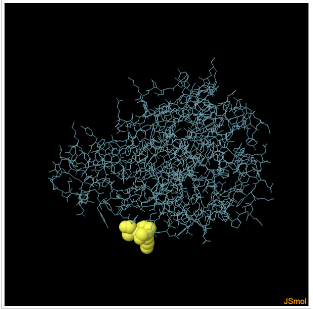

20	Mbov_0675[a,b]	AFM52025_1	5h7w	30.97	1	A:E441, A:E442, A:P447, A:G448, A:N449, A:G450, A:E451, A:R452, A:E453, A:V454, A:P455, A:I456, A:W457, A:R458, A:G459, A:R460, A:A461, A:S549, A:K550, A:K570, A:E571, A:T572, A:K573	23	0.882	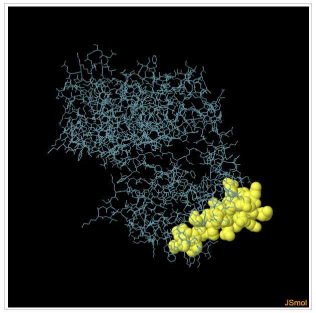
					2	A:K358, A:V377, A:V378, A:D379, A:K380, A:E381, A:T382, A:G383, A:K384, A:V385, A:T386, A:E387, A:V388, A:K389	14	0.862	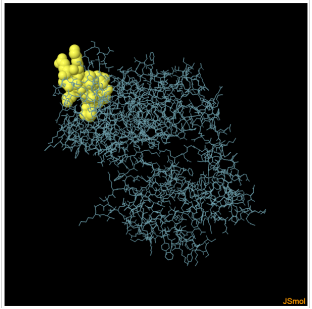
					3	A:W226, A:N227, A:D228, A:K229, A:A230, A:V231, A:A232, A:E233, A:K234, A:V235, A:D239, A:G240, A:K241, A:L242, A:A243, A:V244, A:K245	17	0.858	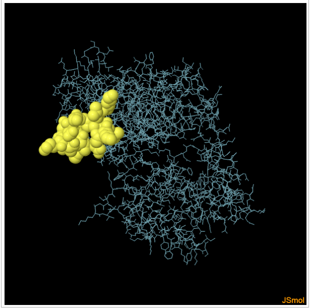

4	A:I408, A:A409, A:S410, A:D413	4	0.846	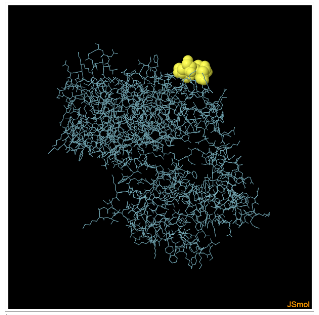
5	A:N562, A:V563, A:S564, A:A565, A:K566, A:I567, A:S568, A:Y569, A:D574, A:K576, A:F577, A:Y579, A:D582, A:S585, A:V586, A:M587, A:I588, A:N589, A:K591, A:K592, A:V593, A:E594, A:K597	23	0.838	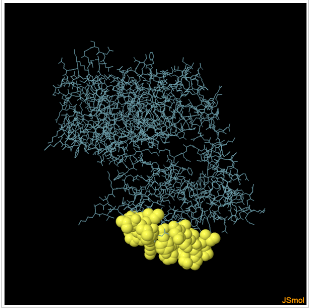
6	A:D350, A:I351, A:E352, A:D355, A:E357	5	0.812	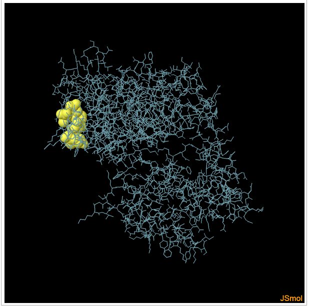

21	Mbov_0739[a,b]	AFM52087_1	4gqo	17.56	7	A:A424, A:D427, A:K428, A:V429, A:N432, A:P434, A:I435, A:E436, A:F437, A:K438, A:D509, A:T510, A:Q511, A:S512, A:G513, A:D514	16	0.802	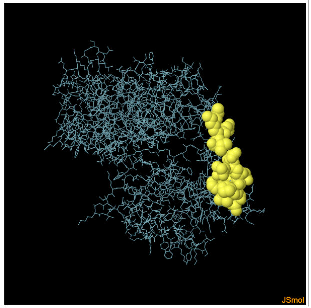
					1	A:S180, A:V181, A:Q182, A:L183, A:N184, A:L185, A:K186, A:V187, A:L188, A:H189, A:K190	11	0.937	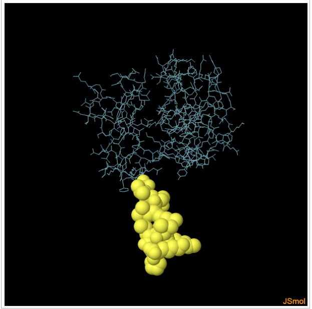
					2	A:N56, A:E58, A:K60, A:K61, A:D62, A:K63, A:D64	7	0.867	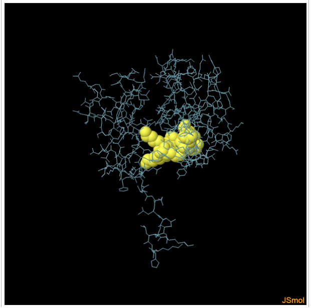

					1	B:I212, B:E213, B:V214, B:Q215, B:S216, B:N217, B:S218, B:K219, B:V220, B:K221, B:V222, B:H223, B:E224, B:L225, B:V226, B:G227, B:T228, B:G229	18	0.923	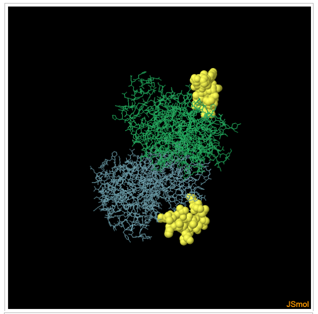
22	Mbov_0743[a,b]	AFM52091_1	4ci7	18.75	2	B:K326, B:K328, B:I330, B:I357, B:Y358, B:Q359, B:D360, B:G361, B:V362, B:D363, B:V364, B:S365, B:D366, B:Y367, B:F368, B:N369, B:I370, B:E371, B:Y372, B:V373, B:K374, B:N375, B:D376, B:G377, B:V378, B:I379	26	0.891	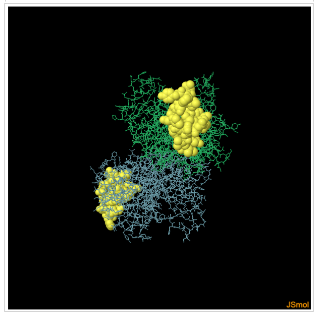
					3	A:K326, A:K328, A:I330, A:Y358, A:Q359, A:D360, A:G361, A:V362, A:D363, A:V364, A:S365, A:D366, A:Y367, A:F368, A:N369, A:I370, A:E371, A:Y372, A:V373, A:K374, A:N375, A:D376, A:G377, A:V378, A:I379, A:L395, A:F396, A:Y397, A:D398, A:Y399, A:E400	31	0.887	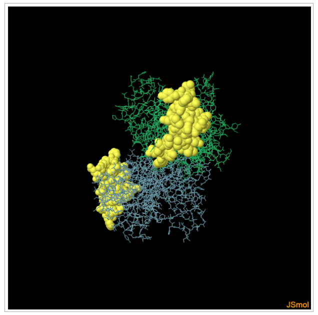

4	A:V214, A:Q215, A:S216, A:N217, A:S218, A:K219, A:V220, A:K221, A:V222, A:G227, A:T228, A:G229	12	0.884	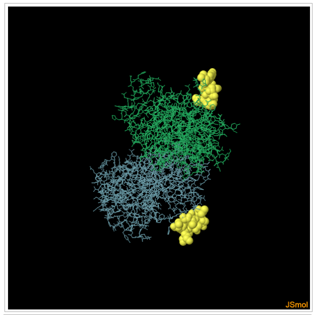
5	A:V144, A:P145, A:N146, A:K147, A:G148, A:A149, A:Y150	7	0.878	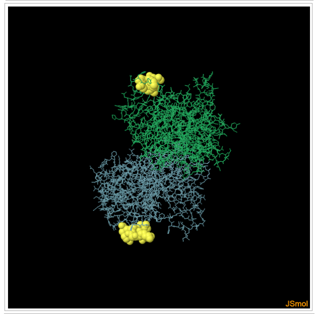
6	B:N315, B:G317, B:V318, B:S320, B:K321, B:N322, B:T323, B:A325	8	0.838	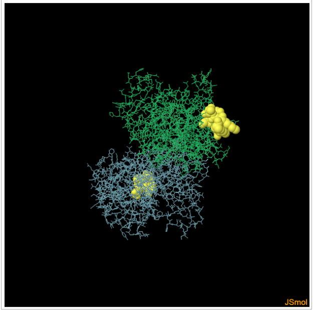

7	B:D140, B:L141, B:Y142, B:Y143, B:V144, B:P145, B:N146, B:K147, B:G148, B:A149, B:Y150, B:H151, B:N152, B:A153, B:L154, B:R155, B:N156, B:Y157, B:N159, B:T162, B:H163	21	0.836	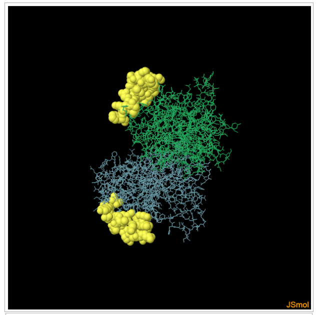
8	A:N315, A:G317, A:V318, A:S320, A:K321, A:N322, A:T323, A:A325	8	0.831	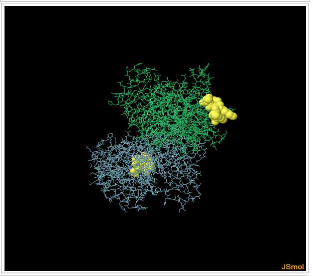
9	A:S333, A:T334, A:Q335	3	0.83	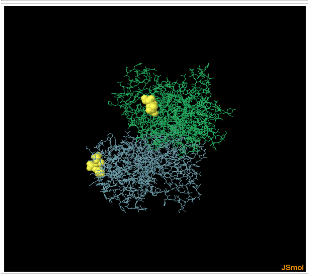

10	A:N152, A:A153, A:N156, A:Y157, A:N159, A:K160, A:Y161, A:K164	8	0.828	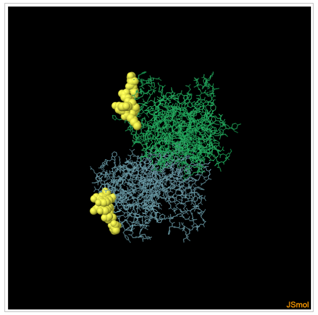
11	B:Y303, B:N332, B:S333, B:T334, B:Q335	5	0.814	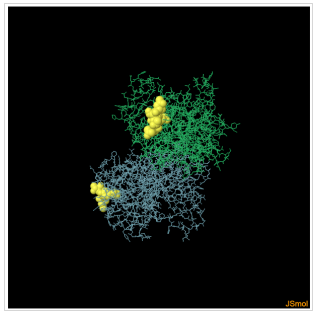
12	B:K394, B:Y397, B:D398	3	0.81	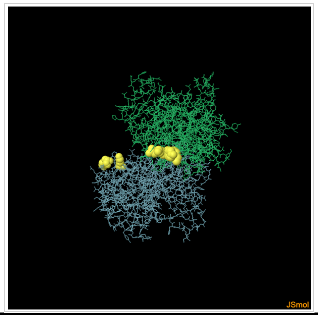

a: related to *M. bovis* HB0801-P1, b: related to P150 and a,b: related to both (*M. bovis* HB0801-P1, and P150).

Formatted: Line spacing: Double, Tab stops: Not at 12.89 cm
